# Diet-Related and Gut-Derived Metabolites and Health Outcomes: A Scoping Review

**DOI:** 10.3390/metabo12121261

**Published:** 2022-12-14

**Authors:** Yuanxi Jia, Xuhao Yang, Lisa M. Wilson, Noel T. Mueller, Cynthia L. Sears, Glenn J. Treisman, Karen A. Robinson

**Affiliations:** 1 Shenzhen Institute of Advanced Technology, Chinese Academy of Sciences, Shenzhen 518055, China; 2 Department of Neurology and Developmental Medicine, Kennedy Krieger Institute, Johns Hopkins University, Baltimore, MD 21205, USA; 3 Department of Health Policy and Management, Bloomberg School of Public Health, Johns Hopkins University, Baltimore, MD 21205, USA; 4 Department of Epidemiology, Bloomberg School of Public Health, Johns Hopkins University, Baltimore, MD 21205, USA; 5 Department of Medicine, Johns Hopkins University School of Medicine, Baltimore, MD 21205, USA

**Keywords:** scoping review, metabolites, microbiome, health, evidence map

## Abstract

We conducted a scoping review to map available evidence about the health impact of gut microbiota-derived metabolites. We searched PubMed and Embase for studies that assessed the health impact of ten metabolites on any health condition: deoxycholate or deoxycholic acid (DCA), lithocholate or lithocholic acid (LCA), glycolithocholate or glycolithocholic acid, glycodeoxycholate or glycodeoxycholic acid, tryptamine, putrescine, d-alanine, urolithins, N-acetylmannosamine, and phenylacetylglutamine. We identified 352 eligible studies with 168,072 participants. Most (326, 92.6%) were case–control studies, followed by cohort studies (14, 4.0%), clinical trials (8, 2.3%), and cross-sectional studies (6, 1.7%). Most studies assessed the following associations: DCA on hepatobiliary disorders (64 studies, 7976 participants), colorectal cancer (19 studies, 7461 participants), and other digestive disorders (27 studies, 2463 participants); LCA on hepatobiliary disorders (34 studies, 4297 participants), colorectal cancers (14 studies, 4955 participants), and other digestive disorders (26 studies, 2117 participants); putrescine on colorectal cancers (16 studies, 94,399 participants) and cancers excluding colorectal and hepatobiliary cancers (42 studies, 4250 participants). There is a need to conduct more prospective studies, including clinical trials. Moreover, we identified metabolites and conditions for which systemic reviews are warranted to characterize the direction and magnitude of metabolite-disease associations.

## 1. Introduction

The human gastrointestinal tract hosts a diverse collection of bacteria, fungi, viruses, archaea, and protozoa. These interconnected microorganisms in the gut produce an extremely diverse reservoir of metabolites from exogenous dietary components or endogenous compounds generated by these microorganisms and the host. These metabolites have diverse effects on host physiology and hemostasis and are detectable in a wide range of biological tissues, including feces, urine, serum, liver, and cerebrospinal fluid [1,2,3]. The disruption of the gut environment may lead to disturbance of the host-microbiota homeostasis, which has been associated with various health conditions [4].

Evidence has been accumulating on the key role played by gut-derived metabolites in health maintenance and disease pathogenesis. To date, the health impact of some metabolites has been synthesized in systematic reviews, such as trimethylamine-n-oxide on diabetes [5] and cardiovascular disorders [6,7,8], and daidzein on diabetes [9,10] and prostate cancer [11]. However, their potential health impact remains unclear for most gut microbial-derived metabolites. Further, what types of evidence exist to support such hypotheses is also unclear. This kind of knowledge is needed to help inform research agendas and determine future research direction.

Evidence mapping is a tool used to systematically identify, organize and summarize the scientific evidence on a broad subject [12,13]. An evidence map summarizes the characteristics of existing literature and determines the level of evidence to identify research gaps as well as research areas where systematic reviews would be worthwhile. Therefore, scoping reviews can be a cost-effective methodology to facilitate evidence-based decision-making about research. This scoping review aims to identify where there is sufficient evidence to conduct systematic reviews to assess the health impact of metabolites for various health conditions using evidence mapping.

## 2. Materials and Methods

### 2.1. Scope of the Review

We developed a protocol for the scoping review and registered it on the Open Science Framework (osf.io/s3etb). Our review was informed by current guidance on conducting and reporting scoping reviews [14,15,16]. During the prioritization phase, we conducted preliminary searches and solicited input from the experts on the Gut Microbiome Committee of the Institute for the Advancement of Food and Nutrition Sciences to determine the metabolites to include in this scoping review. We started with 85 metabolites included in a commercial database maintained by Metabolon, Inc (Durham, NC, USA). and a workshop report developed by the National Institute of Standards and Technology, the BioCollective, and the North America Branch of the International Life Sciences Institute [17,18]. Searches then focused on identifying and classifying metabolites by whether there were human or animal studies or systematic reviews about health impact available (see Table S1. With input from the experts, we identified metabolites that had been studied in humans yet had not been synthesized in a current systematic review. This prioritization process identified ten metabolites: deoxycholate or deoxycholic acid (DCA), lithocholate or lithocholic acid (LCA), glycolithocholate or glycolithocholic acid (GLCA), glycodeoxycholate or glycodeoxycholic acid (GDCA), tryptamine, putrescine, d-alanine, urolithins, N-acetylmannosamine (ManNAc), and phenylacetylglutamine (PAG).

Eligible studies assessed the health impact of oral intake of eligible metabolites or assessed the association of health conditions with the concentration of metabolites in blood, urine, or feces. Any health condition was eligible for this scoping review. We included case–control studies, cohort studies, and clinical trials that recruited at least five participants. We considered a study as a case–control study if it recruited people with the condition and controls separately and assessed the association between the concentration of metabolites in their blood, urine, or feces and health outcomes. Only articles published in English-language journals were included. Reviews, abstracts, and letters were excluded. There was no limit on the date of publication.

### 2.2. Literature Search

We searched PubMed and Embase for eligible studies through August 2021. (The search strategies are provided in Table S2.) We used PICO Portal for literature screening [19]. PICO Portal uses artificial intelligence and machine learning to order citations with those most likely to be eligible presented first and to predict the number of eligible studies in the remaining dataset. We predefined that we would end the manual screening process when at least 95% of eligible studies were identified, per the machine learning prediction. The title and abstract screening were conducted by two authors independently until the system predicted that fewer than 150 eligible citations remained, at which point one author screened 600 more of the machine-prioritized citations. Two authors conducted full-text screening independently, and conflicts were resolved by consensus.

### 2.3. Data Abstraction and Analysis

For each eligible study, we extracted the following data items: study design, study population, sample size, intervention or exposure, and health conditions. We modified categories from the International Classification of Diseases 11th Revision to classify the health conditions [20]. The health conditions assessed by no more than five studies were combined into the “Other” category. The health conditions included cancers, cardiovascular disorders, dermatological disorders, digestive disorders, diabetes/impaired glucose metabolism, metabolic disorders, mental health, neurological disorders, renal disorders, respiratory disorders, and others. Because most included studies were expected to assess digestive disorders and cancers, we used more detailed categories for these two types of conditions: cancers were divided into hepatobiliary cancers, colorectal cancers, and other cancers, while digestive disorders were divided into hepatobiliary disorders, inflammatory bowel disease, and other digestive disorders. Studies on special populations were also recorded, such as infants, children, and pregnant women. The data abstraction was conducted by one author and checked by another. We described the characteristics of included studies and plotted two bubble plots for evidence mapping using R (version 4.1.2).

## 3. Results

**Figure 1:**
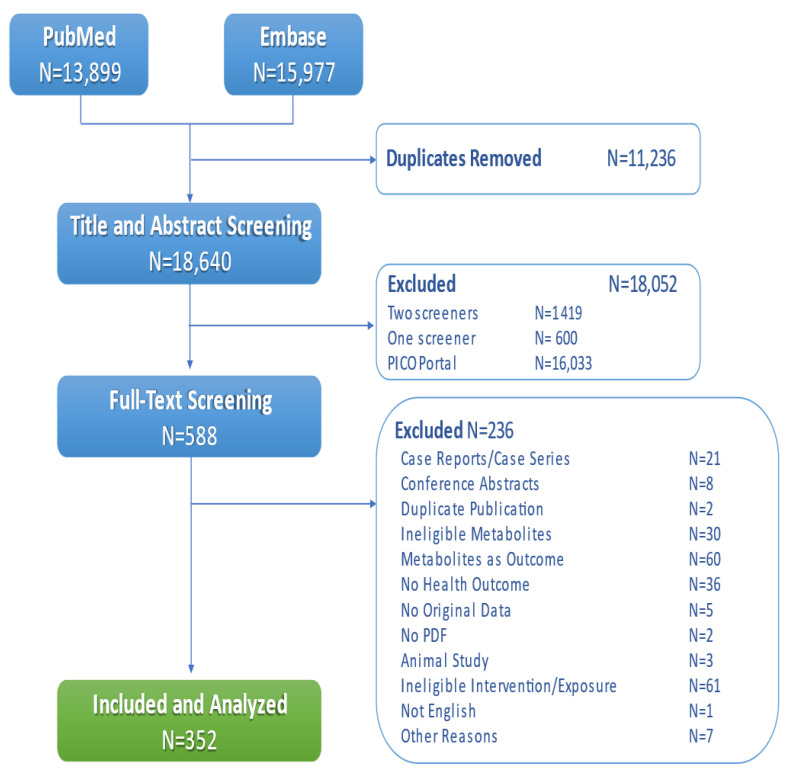
Selection of Included Studies.

The search retrieved 18,640 unique records from PubMed and Embase, and we identified 352 eligible studies. The results of the search and screening are shown in Figure 1, and the 352 included studies are listed in Table S3.

### 3.1. Study Characteristics

Most (326, 92.6%) included studies were case–control studies, followed by cohort studies (14, 4.0%), clinical trials (8, 2.3%), and cross-sectional studies (6, 1.7%). The 352 studies recruited 168,076 participants in total. Cohort studies recruited the most participants (109,075, 64.9%), followed by case–control studies (61,553, 36.6%), cross-sectional studies (2125, 1.3%), and clinical trials (306, 0.2%). Cohort studies also reported the largest median sample size (880.5), followed by cross-sectional studies (381.5), case–control studies (64), and clinical trials (31.5).

**Figure 2:**
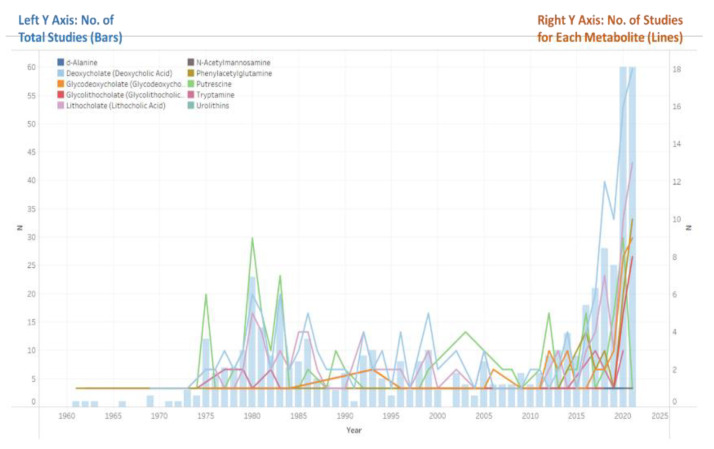
Publishing Year of Included Studies. Note: A study may be counted multiple times if it assesses multiple metabolites.

In Figure 2, we plotted the number of studies against the year of publication. The total number of studies is represented by bars, while the number of studies for each metabolite is represented by lines. The *Y*-axis on the left indicates the number of total studies, while the *Y*-axis on the right indicates the number of studies for each metabolite. The figure shows that the number of published studies increased to the first peak between 1980 and 1983, decreased and stayed stable, and then reached a second peak between 2020 and 2021. The trend of studies assessing putrescine deviated from other metabolites as the number of studies published dropped to only one study in 2021 from nine in 2020.

### 3.2. Metabolites

We identified eligible studies for all ten metabolites of interest (Table 1). Almost half (166, 47.2%) assessed the association between DCA and health outcomes, followed by LCA (111, 31.5%) and putrescine (100, 28.4%). Only three studies assessed ManNAC and d-Alanine, and two assessed urolithins. Studies assessing putrescine recruited the most patients (103,272, 61.4%), followed by DCA (33,231, 19.8%) and PAG (23,931, 14.2%). The discrepancy was mainly driven by a cohort study that recruited more than 80,000 participants to assess the association between putrescine and the risk of colorectal cancer.

### 3.3. Health Outcomes

A variety of health outcomes were assessed by the included studies (Table 2 and Table S3). A total of 80 studies assessed the association between metabolites and hepatobiliary disorders (22.7%), followed by cancers excluding colorectal cancer and hepatobiliary cancer (61, 17.3%), and other digestive disorders (41, 11.6%). However, most participants (100,977, 60.1%) were recruited for studies on colorectal cancer, followed by cardiovascular disorders (16,579, 9.9%) and diabetes or impaired glucose metabolism (11,348, 6.8%). A detailed description of the health outcomes can be found in Table S3.

Most (340, 96.6%) included studies assessed the association between the blood concentration of metabolites and health outcomes, including 212 (60.2%) on blood concentration, 63 (17.5%) on feces concentration, and 89 (24.7%) on urine concentration. Twelve (3.3%) studies assessed the impact of oral intake of metabolites on health outcomes, including eight clinical trials (six on DCA and two on ManNAc) and four cohort studies (all on putrescine).

### 3.4. Participants

Most (322, 91.5%) studies were conducted among adults, while 30 (8.5%) studies were focused on special populations. The 30 studies of special populations included 21 (6.0%) among children, 11 (3.1%) among pregnant women, and 6 (1.7%) among infants. All but one of the studies among pregnant women assessed DCA.

### 3.5. Evidence Maps and Research Gaps

**Figure 3:**
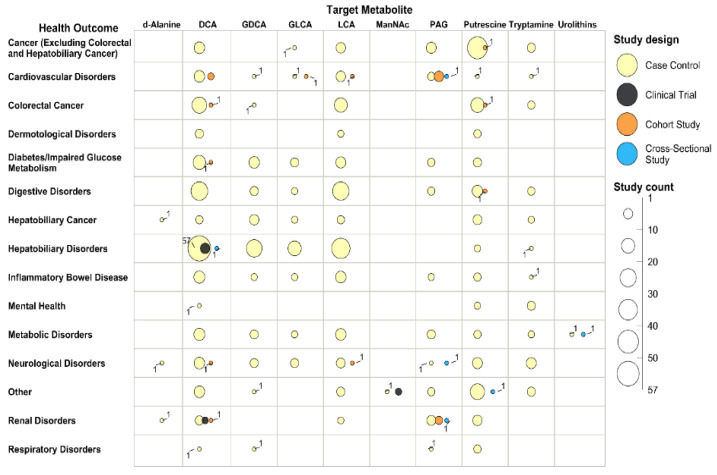
Bubble Plot for Relationships or Patterns between Metabolites, Health Outcomes, and Study Design with Bubble Size Relative to the Number of Studies. Abbreviations: DCA: Deoxycholate (Deoxycholic Acid); LCA: Lithocholate (Lithocholic Acid); GDCA: Glycodeoxycholate (Glycodeoxycholic Acid); PAG: Phenylacetylglutamine; GDCA: Glycolithocholate (Glycolithocholic Acid); ManNAc: N-Acetylmannosamine.

**Figure 4:**
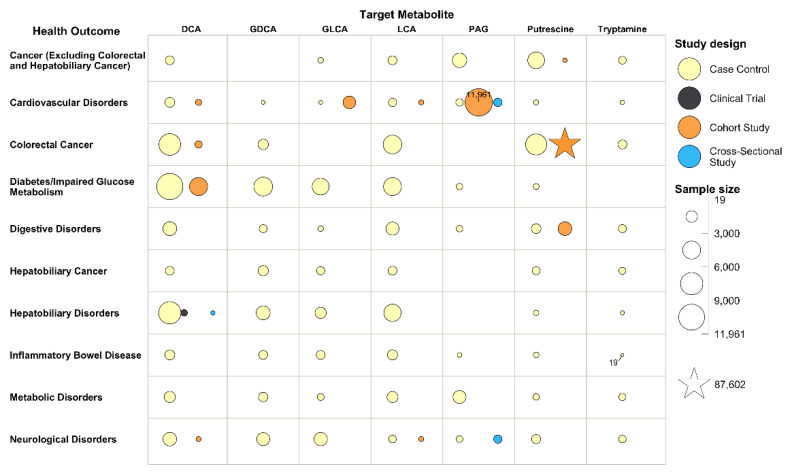
Bubble Plot for Relationships or Pattern between Metabolites, Health Outcomes, and Study Design with Bubble Size Relative to Number of Participants. Abbreviations: DCA: Deoxycholate (Deoxycholic Acid); LCA: Lithocholate (Lithocholic Acid); GDCA: Glycodeoxycholate (Glycodeoxycholic Acid); PAG: Phenylacetylglutamine; GDCA: Glycolithocholate (Glycolithocholic Acid); ManNAc: N-Acetylmannosamine.

We created two bubble plots to identify relationships or patterns among the metabolites, health outcomes, and study designs (Figure 3 and Figure 4). A study was included multiple times if multiple metabolites or health outcomes were reported. The bubble plots were plotted in a two-dimensional grid according to metabolites and health outcomes. Each bubble represents a type of study design, and the size of the bubble is relative to the number of studies (Figure 3) or the number of participants (Figure 4).

The bubble plots show that most studies assessed the following associations: DCA on hepatobiliary disorders (64 studies, 7976 participants), DCA on colorectal cancer (19 studies, 7461 participants), and DCA on digestive disorders excluding hepatobiliary disorders (27 studies, 2463 participants); LCA on hepatobiliary disorders (34 studies, 4297 participants), LCA on colorectal cancers (14 studies, 4955 participants), and LCA on digestive disorders excluding hepatobiliary disorders (26 studies, 2117 participants); putrescine on colorectal cancers (16 studies, 94,399 participants) and cancers excluding colorectal and hepatobiliary cancers (42 studies, 4250 participants).

The bubble plots also highlight three types of research gaps: (1) the studies published are heavily focused on some metabolites, such as DCA, LCA, and putrescine, while very few studies assessed d-Alanine, ManNAc, or urolithins; (2) the studies are heavily focused on health outcomes related to the digestive system, such as hepatobiliary disorders, hepatobiliary cancers, colorectal cancer, and other digestive disorders, while much fewer assessed other health outcomes; and, (3) most included studies were case–control studies.

## 4. Discussion

We identified and mapped 352 studies that assessed the association between ten metabolites and health outcomes to identify areas with sufficient evidence for systematic reviews and areas representing evidence gaps. Although we identified studies for all ten metabolites and for a variety of health outcomes, most studies assessed DCA, LCA, and putrescine as the target metabolites, and digestive system-related disorders and cancers as the target health outcomes, and most were case–control studies. Evidence derived from clinical trials or cohort studies is generally stronger than evidence from case–control or cross-sectional studies [21]. Therefore, there is generally weak evidence on the health impact of these ten metabolites, and future studies are warranted.

### 4.1. Suggestions for Systematic Reviews

The evidence mapping suggests that the evidence from observational studies, mainly case–control studies, on several associations may permit future systematic reviews, namely: the association between DCA and hepatobiliary disorders, DCA and colorectal cancers, DCA and other digestive disorders, LCA and hepatobiliary disorders, LCA and other digestive disorders, LCA and colorectal cancers, putrescine and colorectal cancer, and putrescine and cancers excluding colorectal cancers and hepatobiliary cancers. We did not identify any existing systematic reviews on those associations; therefore, systematic reviews in these areas would be feasible and worthwhile. We did not identify many studies on other associations; researchers may need to wait for more evidence before conducting systematic reviews.

Most included studies assessed the association between the concentration of metabolites in blood, urine, or feces with health outcomes using case–control or cross-sectional design. In those studies, the temporal relationship between exposure and outcome is usually unclear [22]. In other words, it is challenging to determine whether the alternation of concentration of metabolites leads to health outcomes or vice versa. We only identified 13 studies that directly assessed the health impact of oral intake of metabolites that may be of particular interest to researchers. These studies included six clinical trials assessing the health impact of DCA [23,24,25,26,27,28], two clinical trials assessing ManNAc [29,30], one case–control study assessing PAG [31], two cohort studies, and one case–control study assessing putrescine [32,33,34].

### 4.2. Limitations

There were several limitations to our scoping review. First, we did not conduct an exhaustive literature search nor attempt to identify all eligible studies from literature screening. However, with the help of artificial intelligence employed in the screening tool, we are confident that we were able to identify the most eligible studies, and the inclusion of any missing studies would not change our conclusion. Second, we did not conduct a risk of bias assessment on included studies. Thus we could not rule out the possibility that the quality of identified studies is low, potentially limiting the value of systematic review. Third, we did not abstract the outcomes of clinical trials in detail. It was possible that the specific outcomes assessed in included studies were not of interest to researchers performing a systematic review or decision makers using those reviews [35].

## 5. Conclusions

We identified 352 studies assessing the association between ten gut microbiota-derived metabolites and human health, most of which were focused on the health impact of DCA/LCA on digestive system-related disorders and colorectal cancer, as well as putrescine and cancers. Systemic reviews of these metabolites would be useful to characterize the direction and magnitude of metabolite-disease associations and, ultimately, inform decisions, including those about future studies.

## Figures and Tables

**Table 1:** Distribution of Metabolites.

Metabolite	No. of Studies (%)	Sample Size (%)
DCA	166 (47.2)	33,231 (19.8)
LCA	111 (31.5)	18,591 (11.1)
Putrescine	100 (28.4)	103,272 (61.4)
GDCA	48 (13.6)	12,340 (7.3)
PAG	36 (10.2)	23,931 (14.2)
GLCA	30 (8.5)	10,377 (6.2)
Tryptamine	26 (7.4)	2422 (1.4)
ManNAc	3 (0.9)	140 (0.1)
d-Alanine	3 (0.9)	281 (0.2)
Urolithins	2 (0.6)	857 (0.5)
Total	352 (100.0)	168,076 (100.0)

Abbreviations: DCA: Deoxycholate (Deoxycholic Acid); LCA: Lithocholate (Lithocholic Acid); GDCA: Glycodeoxycholate (Glycodeoxycholic Acid); PAG: Phenylacetylglutamine; GDCA: Glycolithocholate (Glycolithocholic Acid); ManNAc: N-Acetylmannosamine.

**Table 2:** Distribution of Health Outcomes.

Health Outcomes	No. of Studies (%)	Sample Size (%)
Hepatobiliary Disorders	80 (22.7)	9786 (5.8)
Cancer (Excluding Colorectal and Hepatobiliary Cancer)	61 (17.3)	7801 (4.6)
Digestive Disorders	41 (11.6)	5891 (3.5)
Other	35 (9.9)	2743 (1.6)
Colorectal Cancer	35 (9.9)	100,977 (60.1)
Neurological Disorders	27 (7.7)	5317 (3.2)
Cardiovascular Disorders	24 (6.8)	16,579 (9.9)
Renal Disorders	23 (6.5)	7804 (4.6)
Diabetes/Impaired Glucose Metabolism	21 (6.0)	11,348 (6.8)
Metabolic Disorders	20 (5.7)	4900 (2.9)
Inflammatory Bowel Disease	15 (4.3)	1379 (0.8)
Hepatobiliary Cancer	14 (4.0)	1906 (1.1)
Mental Health	7 (2.0)	598 (0.4)
Dermatological Disorders	7 (2.0)	410 (0.2)
Respiratory Disorders	6 (1.7)	645 (0.4)
Total	352 (100.0)	168,076 (100.0)

## Supplementary Materials

The following supporting information can be downloaded at: https://www.mdpi.com/article/10.3390/metabo12121261/s1, Table S1: Results of Preliminary Searching: Category of Metabolites According to available Evidence; Table S2: Search Strategy; Table S3: Characteristics of Included Studies
