# Supplementary material for: Diet-Related and Gut-Derived Metabolites and Health Outcomes: A Scoping Review
Source: Metabolites. 2022 Dec 14;12(12):1261. doi: 10.3390/metabo12121261 (PMC9782760; doi:10.3390/metabo12121261)
Supplement: Supplementary file 1 [file metabolites-12-01261-s001.zip › metabolites-2042972-supplementary.pdf]

Table S1 Results of Preliminary Searching: Category of Metabolites According to available Evidence

**Category of Metabolites**	**No. of Systematic Reviews Found**	**No. of Human Studies Found**	**No. of Animal Studies Found**
**Multiple Systematic Reviews Available**			
Ursodeoxycholate	16	-	-
Trimethylamine-n-oxide	18	-	-
Betaine	7	-	-
Daidzein/Genistein	25	-	-
Creatine/Creatinine	22	-	-
Riboflavin	9	-	-
Pyridoxine	34	-	-
Folate	190	-	-
**No or One Systematic Review Available, At Least One Human Study Available**			
Cholate	0	3	-
Deoxycholate	0	2	-
Lithocholate	0	1	4
Ketolithocholate	0	1	-
Tauroursodeoxycholate	0	1	-
Dimethylglycine	0	2	-
Phenylacetate	0	2	-
P-cresol sulfate	0	1	-
3-(4-hydroxyphenyl)lactate	0	2	-
Phenylacetylglutamine	0	1	-
Phenylacetylglycine	0	1	-
4-hydroxycinnamate	0	1	-

Indolelactate	0	1	-
N-acetyltryptophan	0	3	-
3-indoxyl sulfate	0	3	-
Indolepropionate	0	2	-
Hippurate	0	2	-
2-hydroxyhippurate	0	1	-
3-hydroxyhippurate	0	1	-
Xylose	0	2	-
Lactate	0	1	-
Formate	0	4	-
Lyso-PC	1	-	-
Monacylglycerol	0	1	1
Putrescine	0	1	7
Spermidine	0	4	-
**Only Animal Studies Available**			
Dehydrocholate	0	0	1
Hyocholate	0	0	2
4-ethylphenylsulfate	0	0	1
3-hydroxybenzoate	0	0	1
4-hydroxybenzoate	0	0	1
3,4-dihydroxybenzoate	0	0	3
P-hydroxybenzaldehyde	0	0	1
Vitexin	0	0	2
Succinate	0	0	1
Valerate	0	0	1
Isovalerate	0	0	3

Lyso-PE	0	0	1
Spermine	0	0	7
Cyclic Dipeptides	0	0	2
**No Human or Animal Studies Available**			
Glycodeoxycholate	0	0	0
Ketodeoxycholate	0	0	0
Glycolithocholate sulfate	0	0	0
Taurolithocholate	0	0	0
Taurolithocholate sulfate	0	0	0
Diketolithocholate	0	0	0
Glycocholenate sulfate	0	0	0
Taurocholenate sulfate	0	0	0
Glycoursodeoxycholate	0	0	0
Phenyllactate	0	0	0
4-hydroxyphenylpyruvate	0	0	0
4-hydroxyphenylacetate	0	0	0
3-hydroxyphenylacetate	0	0	0
3,4-dihydroxyphenylacetate	0	0	0
2-(4-hydroxyphenyl)propionate	0	0	0
3-(3-hydroxyphenyl)propionate	0	0	0
3-(4-hydroxyphenyl)propionate	0	0	0
3-phenylpropionate	0	0	0
Phenol sulfate	0	0	0
Indoleacetate	0	0	0
Indole-3-carboxylic acid	0	0	0
Skatol	0	0	0

Indoleacetylglutamine	0	0	0
4-hydroxyhippurate	0	0	0
2,4,6-trihydroxybenzoate	0	0	0
Methyl-4-hydroxybenzoate	0	0	0
3-(2-hydroxyphenyl)propionate	0	0	0
Ketoisovalerate	0	0	0
Methylpropionate	0	0	0
Cadaverine	0	0	0
Urobilinogen	0	0	0

'-' indicates when no search was conducted. For instance, no need to search for human or animal studies when systematic reviews are available.

Table S2 Search Strategy

PubMed	
**#1 Name/Synonyms of Metabolites**	Deoxycholic Acid[tiab] OR Desoxycholic Acid[tiab] OR Deoxycholate[tiab] OR Kybella[tiab] OR Cholanoic Acid[tiab]<br>OR<br>Putrescine[tiab] OR 1,4-Diaminobutane[tiab] OR 1,4 Diaminobutane[tiab] OR Tetramethylenediamine[tiab] OR 1,4-Butanediamine[tiab] OR 1,4 Butanediamine[tiab]<br>OR<br>Lithocholic Acid[tiab] OR Lithocholate[tiab]<br>OR<br>Tryptamine[tiab] OR Tryptamines[tiab]<br>OR<br>D-Alanine[tiab] OR D Alanine[tiab]<br>OR<br>Urolithins[tiab] OR Urolithin[tiab]<br>OR<br>Glycodeoxycholate[tiab] OR Glycodeoxycholic Acid[tiab] OR Deoxycholylglycine[tiab]<br>OR<br>N-Acetylmannosamine[tiab] OR 2-acetamido-2-deoxy-D-mannose[tiab] OR N-acetyl-D-mannosamine[tiab]<br>OR<br>Phenylacetylglutamine[tiab]<br>OR<br>Glycolithocholic Acid[tiab] OR Glycolithocholate[tiab] OR lithocholylglycine[tiab]
**#2 Language Restriction**	English[la]
**#3 Publication Type Restriction**	Review[pt]
**#4 Study Subject**	Animals[mh] NOT humans[mh]
**#5 Final Strategy**	#1 AND #2 NOT #3 NOT #4<br>Search yielded 13,899 records on Aug 25, 2021.
**Embase**	

**#1 Name/Synonyms of Metabolites**	('Deoxycholic Acid' OR 'Desoxycholic Acid' OR 'Deoxycholate' OR 'Kybella' OR 'Cholanoic Acid'<br>OR<br>Putrescine OR '1,4-Diaminobutane' OR '1,4 Diaminobutane' OR Tetramethylenediamine OR '1,4-Butanediamine' OR '1,4 Butanediamine'<br>OR<br>'Lithocholic Acid' OR Lithocholate<br>OR<br>Tryptamine OR Tryptamines<br>OR<br>'D-Alanine' OR 'D Alanine'<br>OR<br>Urolithins OR Urolithin<br>OR<br>Glycodeoxycholate OR 'Glycodeoxycholic Acid' OR Deoxycholylglycine<br>OR<br>'N-Acetylmannosamine' OR '2-acetamido-2-deoxy-D-mannose' OR 'N-acetyl-D-mannosamine'<br>OR<br>Phenylacetylglutamine<br>OR<br>'Glycolithocholic Acid' OR Glycolithocholate OR lithocholylglycine):ti,ab
**#2 Language Restriction**	English:la
**#3 Publication Type Restriction**	Review/it
**#4 Study Subject**	[Animals]/lim NOT [Humans]/lim
**#5 Final Strategy**	#1 AND #2 NOT #3 NOT #4<br>Search yielded 15,977 records on Aug 25, 2021.

Table S3 Characteristics of Included Studies

Title (Reference Number)	Year	First Author	Journal	Metabolite	Exposure Type	Study Design	Population	Health Outcome Details	Health Outcome Classification	Sample Size
Altered polyamine excretion in Duchenne muscular dystrophy.	1981	D H Russell	Neurology	Putrescine	Urine Concentration	Case Control	Children	Duchenne Muscular Dystrophy	Neurological Disorders	308
Evidence for defective primary bile acid secretion in children with progressive familial intrahepatic cholestasis (Byler disease).	1994	E Jacquemin	European journal of pediatrics	DCA	Blood Concentration	Case Control	Children	Familial Intrahepatic Cholestasis	Hepatobiliary Disorders	15
Evidence for defective primary bile acid secretion in children with progressive familial intrahepatic cholestasis (Byler disease).	1994	E Jacquemin	European journal of pediatrics	LCA	Blood Concentration	Case Control	Children	Familial Intrahepatic Cholestasis	Hepatobiliary Disorders	15
Hyperserotonemia and amine metabolites in autistic and retarded children.	1977	H G Hanley	Archives of general psychiatry	Tryptamine	Urine Concentration	Case Control	Children	Autistic Retarded	Neurological Disorders	81
Serum bile acid composition in patients with cystic fibrosis.	1985	K D Setchell	Clinica chimica acta; international journal of clinical chemistry	DCA	Blood Concentration	Case Control	Children	Cystic Fibrosis Involving Pancreas	Digestive Disorders	14
Serum bile acid composition in patients with cystic fibrosis.	1985	K D Setchell	Clinica chimica acta; international journal of clinical chemistry	LCA	Blood Concentration	Case Control	Children	Cystic Fibrosis Involving Pancreas	Digestive Disorders	14
Altered bile acid metabolism in childhood functional constipation: inactivation of secretory bile acids by sulfation in a subset of patients.	2008	Alan F Hofmann	Journal of pediatric gastroenterology and nutrition	DCA	Feces Concentration	Case Control	Children	Constipation	Digestive Disorders	207
Altered bile acid metabolism in childhood functional constipation: inactivation of secretory bile acids by sulfation in a subset of patients.	2008	Alan F Hofmann	Journal of pediatric gastroenterology and nutrition	LCA	Feces Concentration	Case Control	Children	Constipation	Digestive Disorders	207
Serum and urine polyamines in normal and in short children.	1979	D Rudman	The Journal of clinical investigation	Putrescine	Blood Concentration	Case Control	Children	Short due to Growth Hormone Deficiency Turner'S Syndrome	Other	162
Serum and urine polyamines in normal and in short children.	1979	D Rudman	The Journal of clinical investigation	Putrescine	Urine Concentration	Case Control	Children	Short due to Growth Hormone Deficiency Turner'S Syndrome	Other	162
Genetic Polymorphisms, Mediterranean Diet and Microbiota-Associated Urolithin Metabotypes can Predict Obesity in Childhood-Adolescence.	2020	Adrián Cortés-Martín	Scientific reports	Urolithins	Urine Concentration	Case Control	Children	Obesity	Metabolic Disorders	415

Suppressed hepatic bile acid signalling despite elevated production of primary and secondary bile acids in NAFLD.	2018	Na Jiao	Gut	DCA	Blood Concentration	Case Control	Children	Non-Alcoholic Fatty Liver Disease	Hepatobiliary Disorders	27
Trace amines and Tourette's syndrome	1993	Baker, G. B.	Neurochemical Research	Tryptamine	Urine Concentration	Case Control	Children	Tourette's Syndrome	Neurological Disorders	82
Indole Tryptophan Metabolism and Cytokine S100B in Children with Attention-Deficit/Hyperactivity Disorder: Daily Fluctuations, Responses to Methylphenidate, and Interrelationship with Depressive Symptomatology.	2020	Luisa Fernández-López	Journal of child and adolescent psychopharmacology	Tryptamine	Blood Concentration	Case Control	Children	Attention-Deficit/Hyperactivity Disorder<br>Depressive Symptoms	Mental Disorders	179
Polyamines are increased in obese children and are related to markers of oxidative/nitrosative stress and angiogenesis.	2011	Pilar Codoñer-Franch	The Journal of clinical endocrinology and metabolism	Putrescine	Blood Concentration	Case Control	Children	Obesity	Metabolic Disorders	102
Changes in urine polyamines in childhood leukemias	1981	Garnica, A.	Annals of Clinical and Laboratory Science	Putrescine	Urine Concentration	Case Control	Children	Hematological Malignancies<br>Solid Tumors	Cancer (Excluding Colorectal and Hepatobiliary Cancer)	36
Urinary Metabolites Enable Differential Diagnosis and Therapeutic Monitoring of Pediatric Inflammatory Bowel Disease.	2021	Mai Yamamoto	Metabolites	PAG	Urine Concentration	Case Control	Children	Crohn's Disease<br>Ulcerative Colitis	Inflammatory Bowel Disease	26
Urinary Metabolic Phenotyping Reveals Differences in the Metabolic Status of Healthy and Inflammatory Bowel Disease (IBD) Children in Relation to Growth and Disease Activity.	2016	Francois-Pierre Martin	International journal of molecular sciences	PAG	Urine Concentration	Case Control	Children	Crohn's Disease<br>Ulcerative Colitis	Inflammatory Bowel Disease	48
Decreased secondary faecal bile acids in children with ulcerative colitis and Clostridioides difficile infection.	2021	Sarah Rotondo-Trivette	Alimentary pharmacology & therapeutics	DCA	Feces Concentration	Case Control	Children	Ulcerative Colitis	Inflammatory Bowel Disease	78
Decreased secondary faecal bile acids in children with ulcerative colitis and Clostridioides difficile infection.	2021	Sarah Rotondo-Trivette	Alimentary pharmacology & therapeutics	LCA	Feces Concentration	Case Control	Children	Ulcerative Colitis	Inflammatory Bowel Disease	78
Environmental Enteric Dysfunction Is Associated With Altered Bile Acid Metabolism.	2017	Richard D Semba	Journal of pediatric gastroenterology and nutrition	GDCA	Blood Concentration	Case Control	Children	Environmental Enteric Dysfunction	Digestive Disorders	313
Environmental Enteric Dysfunction Is Associated With Altered Bile Acid Metabolism.	2017	Richard D Semba	Journal of pediatric gastroenterology and nutrition	LCA	Blood Concentration	Case Control	Children	Environmental Enteric Dysfunction	Digestive Disorders	313
Environmental Enteric Dysfunction Is Associated With Altered Bile Acid Metabolism.	2017	Richard D Semba	Journal of pediatric gastroenterology and nutrition	DCA	Blood Concentration	Case Control	Children	Environmental Enteric Dysfunction	Digestive Disorders	313
Serum bile acid level and fatty acid composition in Chinese children with non-alcoholic fatty liver disease.	2017	Li Ping Lu	Journal of digestive diseases	DCA	Blood Concentration	Case Control	Children	Non-Alcoholic Fatty Liver	Hepatobiliary Disorders	76

Serum bile acid level and fatty acid composition in Chinese children with non-alcoholic fatty liver disease.	2017	Li Ping Lu	Journal of digestive diseases	GDCA	Blood Concentration	Case Control	Children	Non-Alcoholic Fatty Liver	Hepatobiliary Disorders	76
Serum bile acid level and fatty acid composition in Chinese children with non-alcoholic fatty liver disease.	2017	Li Ping Lu	Journal of digestive diseases	LCA	Blood Concentration	Case Control	Children	Non-Alcoholic Fatty Liver	Hepatobiliary Disorders	76
Serum bile acid level and fatty acid composition in Chinese children with non-alcoholic fatty liver disease.	2017	Li Ping Lu	Journal of digestive diseases	GLCA	Blood Concentration	Case Control	Children	Non-Alcoholic Fatty Liver	Hepatobiliary Disorders	76
Excretion of polyamines by children with Beckwith's syndrome.	1980	G B Barlow	Archives of disease in childhood	Putrescine	Urine Concentration	Case Control	Children	Beckwith's Syndrome	Other	17
Altered polyamine metabolism in cystic fibrosis.	1979	D H Russell	Pediatric research	Putrescine	Urine Concentration	Case Control	Children	Cystic Fibrosis, Unclassified	Other	31
Sulphated lithocholic acid conjugates in serum from children with hepatic and intestinal diseases.	1985	F Kuipers	Scandinavian journal of gastroenterology	LCA	Blood Concentration	Case Control	Children	Acute Lymphatic Leukaemia<br>Hepatic Disorders<br>Intestinal Disorders	Hepatobiliary Disorders	279
Sulphated lithocholic acid conjugates in serum from children with hepatic and intestinal diseases.	1985	F Kuipers	Scandinavian journal of gastroenterology	LCA	Blood Concentration	Case Control	Children	Acute Lymphatic Leukaemia<br>Hepatic Disorders<br>Intestinal Disorders	Digestive Disorders	279
Sulphated lithocholic acid conjugates in serum from children with hepatic and intestinal diseases.	1985	F Kuipers	Scandinavian journal of gastroenterology	LCA	Blood Concentration	Case Control	Children	Acute Lymphatic Leukaemia<br>Hepatic Disorders<br>Intestinal Disorders	Cancer (Excluding Colorectal and Hepatobiliary Cancer)	279
Fecal polyamine concentration in children with and without nutrient malabsorption.	1997	P Forget	Journal of pediatric gastroenterology and nutrition	Putrescine	Feces Concentration	Case Control	Children	Malabsorption<br>Poor Growth	Metabolic Disorders	33
Studies of the free faecal amines of infants with gastroenteritis and of healthy infants.	1986	K E Murray	Gut	Tryptamine	Feces Concentration	Case Control	Infants	Gastroenteritis	Digestive Disorders	57
Studies of the free faecal amines of infants with gastroenteritis and of healthy infants.	1986	K E Murray	Gut	Putrescine	Feces Concentration	Case Control	Infants	Gastroenteritis	Digestive Disorders	57
Gut Microbiota Dysbiosis Is Associated with Altered Bile Acid Metabolism in Infantile Cholestasis.	2019	Yizhong Wang	mSystems	DCA	Feces Concentration	Case Control	Infants	Cholestasis<br>Elevated AST And ALT	Hepatobiliary Disorders	196
Anemia in infancy is associated with alterations in systemic metabolism and microbial structure and function in a sex-specific manner: an observational study.	2018	Shannon McClorry	The American journal of clinical nutrition	Putrescine	Feces Concentration	Cross-Sectional	Infants	Anemia	Other	95
Pathologic versus physiologic cholestasis: elevated serum concentration of a secondary	1981	W F Balistreri	The Journal of pediatrics	LCA	Blood Concentration	Case Control	Infants	Cholestasis	Hepatobiliary Disorders	236

bile acid in the presence of hepatobiliary disease.										
Urinary polyamines in preterm infants.	1981	M Perlman	Clinica chimica acta; international journal of clinical chemistry	Putrescine	Urine Concentration	Case Control	Infants	Preterm	Other	35
Serum bile acid fractions in neonates on total parenteral nutrition is lithocholic acid responsible for the occurrence of cholestasis?	1992	A Kubota	Asia Pacific journal of clinical nutrition	LCA	Blood Concentration	Case Control	Infants	Intrahepatic Cholestasis	Hepatobiliary Disorders	25
Serum bile acid fractions in neonates on total parenteral nutrition is lithocholic acid responsible for the occurrence of cholestasis?	1992	A Kubota	Asia Pacific journal of clinical nutrition	DCA	Blood Concentration	Case Control	Infants	Intrahepatic Cholestasis	Hepatobiliary Disorders	25
Comparative measurements of urinary polyamines in early morning and 24-hour urine specimens.	1983	K Shinpo	Clinica chimica acta; international journal of clinical chemistry	Putrescine	Urine Concentration	Case Control	Adults	Blood Cancer<br>Solid Cancer	Cancer (Excluding Colorectal and Hepatobiliary Cancer)	78
High performance liquid chromatography-mass spectrometry for metabonomics: potential biomarkers for acute deterioration of liver function in chronic hepatitis B.	2006	Jun Yang	Journal of proteome research	GDCA	Blood Concentration	Case Control	Adults	Chronic Hepatitis B	Hepatobiliary Disorders	87
Altered interactions of tryptophan metabolites in first-episode neuroleptic-naive patients with schizophrenia.	2010	J K Yao	Molecular psychiatry	Tryptamine	Blood Concentration	Case Control	Adults	Schizophrenia	Mental Disorders	55
Increased cholesterol and decreased fluidity of red cell membranes (spur cell anemia) in progressive intrahepatic cholestasis.	1981	W F Balistreri	Pediatrics	LCA	Blood Concentration	Case Control	Adults	Intrahepatic Cholestasis	Hepatobiliary Disorders	18
Increased cholesterol and decreased fluidity of red cell membranes (spur cell anemia) in progressive intrahepatic cholestasis.	1981	W F Balistreri	Pediatrics	DCA	Blood Concentration	Case Control	Adults	Intrahepatic Cholestasis	Hepatobiliary Disorders	18
The relationship between microbiota and polyamine concentration in the human intestine: a pilot study.	2007	Mitsuharu Matsumoto	Microbiology and immunology	Putrescine	Feces Concentration	Case Control	Adults	Multiple Cerebral Infarction<br>Left Femur Neck Fracture<br>Hydrocephalus<br>Epilepsy<br>Cerebral Hemorrhage<br>Schizophrenia	Mental Disorders	24
The relationship between microbiota and polyamine concentration in the human intestine: a pilot study.	2007	Mitsuharu Matsumoto	Microbiology and immunology	Putrescine	Feces Concentration	Case Control	Adults	Multiple Cerebral Infarction<br>Left Femur Neck Fracture	Other	24

								Hydrocephalus<br>Epilepsy<br>Cerebral Hemorrhage<br>Schizophrenia		
The relationship between microbiota and polyamine concentration in the human intestine: a pilot study.	2007	Mitsuharu Matsumoto	Microbiology and immunology	Putrescine	Feces Concentration	Case Control	Adults	Multiple Cerebral Infarction<br>Left Femur Neck Fracture<br>Hydrocephalus<br>Epilepsy<br>Cerebral Hemorrhage<br>Schizophrenia	Neurological Disorders	24
Polyamines in colorectal cancer. Evaluation of polyamine concentrations in the colon tissue, serum, and urine of 50 patients with colorectal cancer.	1990	C Löser	Cancer	Putrescine	Urine Concentration	Case Control	Adults	Colorectal Cancer<br>Nonmalignant Gastrointestinal Diseases	Digestive Disorders	120
Polyamines in colorectal cancer. Evaluation of polyamine concentrations in the colon tissue, serum, and urine of 50 patients with colorectal cancer.	1990	C Löser	Cancer	Putrescine	Blood Concentration	Case Control	Adults	Colorectal Cancer<br>Nonmalignant Gastrointestinal Diseases	Colorectal Cancer	120
Polyamines in colorectal cancer. Evaluation of polyamine concentrations in the colon tissue, serum, and urine of 50 patients with colorectal cancer.	1990	C Löser	Cancer	Putrescine	Urine Concentration	Case Control	Adults	Colorectal Cancer<br>Nonmalignant Gastrointestinal Diseases	Colorectal Cancer	120
Polyamines in colorectal cancer. Evaluation of polyamine concentrations in the colon tissue, serum, and urine of 50 patients with colorectal cancer.	1990	C Löser	Cancer	Putrescine	Blood Concentration	Case Control	Adults	Colorectal Cancer<br>Nonmalignant Gastrointestinal Diseases	Digestive Disorders	120
Lithocholic acid, a bacterial metabolite reduces breast cancer cell proliferation and aggressiveness.	2018	Edit Mikó	Biochimica et biophysica acta. Bioenergetics	LCA	Blood Concentration	Case Control	Adults	Breast Cancer	Cancer (Excluding Colorectal and Hepatobiliary Cancer)	112
Lithocholic acid, a bacterial metabolite reduces breast cancer cell proliferation and aggressiveness.	2018	Edit Mikó	Biochimica et biophysica acta. Bioenergetics	LCA	Feces Concentration	Case Control	Adults	Breast Cancer	Cancer (Excluding Colorectal and Hepatobiliary Cancer)	112
The clinical value of urinary polyamine analyses in cancer patients.	1980	G Milano	Oncodevelopmental biology and medicine : the journal of the International Society for	Putrescine	Urine Concentration	Case Control	Adults	Cancer	Cancer (Excluding Colorectal and	207

			Oncodevelopmental Biology and Medicine						Hepatobiliary Cancer)	
Urinary polyamine excretion patterns in patients with epithelial ovarian cancer.	1989	F Lawton	Gynecologic and obstetric investigation	Putrescine	Urine Concentration	Case Control	Adults	Epithelial Ovarian Cancer	Cancer (Excluding Colorectal and Hepatobiliary Cancer)	100
Differential diagnostic value in hepatobiliary disease of serum conjugated bile acid concentrations and some routine liver tests assessed by discriminant analysis	1983	K Linnet	Clinica chimica acta; international journal of clinical chemistry	GDCA	Blood Concentration	Case Control	Adults	Hepatic Cirrhosis<br>Acute Infectious Hepatitis	Hepatobiliary Disorders	59
Vitamin E deficiency in adults with chronic liver disease.	1985	R J Sokol	The American journal of clinical nutrition	LCA	Blood Concentration	Case Control	Adults	Primary Biliary Cirrhosis<br>Other Chronic Liver Diseases	Hepatobiliary Disorders	82
The Relation between Polyphenols and Body Composition in US Hispanics/Latinos: Results from the Hispanic Community Health Study/Study of Latinos (HCHS/SOL) Study of Latinos Nutrition and Physical Activity Assessment Study (SOLNAS).	2017	Nour Makarem	Current developments in nutrition	Urolithins	Urine Concentration	Cross-Sectional	Adults	Body Mass Index	Metabolic Disorders	442
Peripheral serum metabolomic profiles inform central cognitive impairment.	2020	Jingye Wang	Scientific reports	GLCA	Blood Concentration	Case Control	Adults	Mild Cognitive Impairment<br>Alzheimer’s Disease	Neurological Disorders	566
Gut-Derived Metabolite Phenylacetylglutamine and White Matter Hyperintensities in Patients With Acute Ischemic Stroke.	2021	Fang Yu	Frontiers in aging neuroscience	PAG	Blood Concentration	Cross-Sectional	Adults	White Matter Hyperintensity Burden in Acute Ischemic Stroke	Neurological Disorders	595
Fasting and postprandial serum concentrations of glycine- and taurine-conjugated bile acids in Crohn's disease.	1983	K Linnet	Scandinavian journal of gastroenterology	DCA	Blood Concentration	Case Control	Adults	Crohn's Disease	Inflammatory Bowel Disease	34
Changes in blood polyamine levels in human acute pancreatitis.	2009	Hai-Tao Jin	Scandinavian journal of gastroenterology	Putrescine	Blood Concentration	Case Control	Adults	Acute Pancreatitis	Digestive Disorders	48
Bile acid quantification of 20 plasma metabolites identifies lithocholic acid as a putative biomarker in Alzheimer's disease.	2018	Josef Marksteiner	Metabolomics : Official journal of the Metabolomic Society	LCA	Blood Concentration	Case Control	Adults	Mild Cognitive Impairment<br>Alzheimer'S Disease	Neurological Disorders	80
Bile acid quantification of 20 plasma metabolites identifies lithocholic acid as a putative biomarker in Alzheimer's disease.	2018	Josef Marksteiner	Metabolomics : Official journal of the Metabolomic Society	DCA	Blood Concentration	Case Control	Adults	Mild Cognitive Impairment<br>Alzheimer'S Disease	Neurological Disorders	80

Bile acid quantification of 20 plasma metabolites identifies lithocholic acid as a putative biomarker in Alzheimer's disease.	2018	Josef Marksteiner	Metabolomics : Official journal of the Metabolomic Society	GDCA	Blood Concentration	Case Control	Adults	Mild Cognitive Impairment Alzheimer'S Disease	Neurological Disorders	80
Bile acid quantification of 20 plasma metabolites identifies lithocholic acid as a putative biomarker in Alzheimer's disease.	2018	Josef Marksteiner	Metabolomics : Official journal of the Metabolomic Society	GLCA	Blood Concentration	Case Control	Adults	Mild Cognitive Impairment Alzheimer'S Disease	Neurological Disorders	80
Polyamine compartmentalization in various human disease states.	1978	K D Cooper	Clinica chimica acta; international journal of clinical chemistry	Putrescine	Blood Concentration	Case Control	Adults	Psoriasis Hereditary Elliptocytosis Chronic Lymphocytic Leukemia Lung Cancer Non-Hodgkin's Lymphoma Sickle Cell Anemia with Mild Psoriasis Progeria	Cancer (Excluding Colorectal and Hepatobiliary Cancer)	31
Polyamine compartmentalization in various human disease states.	1978	K D Cooper	Clinica chimica acta; international journal of clinical chemistry	Putrescine	Blood Concentration	Case Control	Adults	Psoriasis Hereditary Elliptocytosis Chronic Lymphocytic Leukemia Lung Cancer Non-Hodgkin's Lymphoma Sickle Cell Anemia with Mild Psoriasis Progeria	Other	31
Polyamine compartmentalization in various human disease states.	1978	K D Cooper	Clinica chimica acta; international journal of clinical chemistry	Putrescine	Blood Concentration	Case Control	Adults	Psoriasis Hereditary Elliptocytosis Chronic Lymphocytic Leukemia Lung Cancer Non-Hodgkin's Lymphoma Sickle Cell Anemia with Mild Psoriasis Progeria	Dermotological Disorders	31
An analysis of lipoproteins, bile acids, and red cell membranes associated with target cells and spur cells in patients with liver disease.	1972	R A Cooper	The Journal of clinical investigation	DCA	Blood Concentration	Case Control	Adults	Liver Cirrhosis	Hepatobiliary Disorders	59

Bile acid metabolism in heterozygous familial hypercholesterolaemia: a study comparing affected and unaffected siblings of four kindreds.	1988	B Angelin	European journal of clinical investigation	DCA	Blood Concentration	Case Control	Adults	Familial Hypercholesterolaemia	Hepatobiliary Disorders	21
Biogenic amines derived from tryptophan in systemic and cutaneous scleroderma.	1979	A Stachów	Acta dermato-venereologica	Tryptamine	Urine Concentration	Case Control	Adults	Systemic Scleroderma	Other	64
Mechanism for the transit-induced increase in colonic deoxycholic acid formation in cholesterol cholelithiasis.	2000	L A Thomas	Gastroenterology	DCA	Blood Concentration	Case Control	Adults	Cholesterol Gallbladder Stones	Hepatobiliary Disorders	40
Pathological changes in platelet histamine oxidases in atopic eczema.	1993	R Kiehl	Mediators of inflammation	Putrescine	Blood Concentration	Case Control	Adults	Atopic Eczema	Dermotological Disorders	34
Amine metabolite profile of normal and uremic urine using gas chromatography--mass spectrometry.	1982	T Ohki	Journal of chromatography	Putrescine	Urine Concentration	Case Control	Adults	Uremia	Renal Disorders	16
Polyamines as biological markers in malignant lymphomas.	1982	A Thyss	European journal of cancer & clinical oncology	Putrescine	Urine Concentration	Cohort Study	Adults	Lymphomas Disorders	Cancer (Excluding Colorectal and Hepatobiliary Cancer)	67
Urinary bile acid excretion in correlation to liver histopathology in cystic fibrosis.	1980	B Arborgh	Scandinavian journal of gastroenterology	LCA	Urine Concentration	Case Control	Adults	Cystic Fibrosis	Other	44
Urinary bile acid excretion in correlation to liver histopathology in cystic fibrosis.	1980	B Arborgh	Scandinavian journal of gastroenterology	DCA	Urine Concentration	Case Control	Adults	Cystic Fibrosis	Other	44
Differentiated quantification of human bile acids in serum by high-performance liquid chromatography-tandem mass spectrometry.	2005	Ines Burkard	Journal of chromatography. B, Analytical technologies in the biomedical and life sciences	GDCA	Blood Concentration	Case Control	Adults	Cholestasis	Hepatobiliary Disorders	23
Differentiated quantification of human bile acids in serum by high-performance liquid chromatography-tandem mass spectrometry.	2005	Ines Burkard	Journal of chromatography. B, Analytical technologies in the biomedical and life sciences	LCA	Blood Concentration	Case Control	Adults	Cholestasis	Hepatobiliary Disorders	23
Differentiated quantification of human bile acids in serum by high-performance liquid chromatography-tandem mass spectrometry.	2005	Ines Burkard	Journal of chromatography. B, Analytical technologies in the biomedical and life sciences	GLCA	Blood Concentration	Case Control	Adults	Cholestasis	Hepatobiliary Disorders	23
Differentiated quantification of human bile acids in serum by high-performance liquid chromatography-tandem mass spectrometry.	2005	Ines Burkard	Journal of chromatography. B, Analytical technologies in the biomedical and life sciences	DCA	Blood Concentration	Case Control	Adults	Cholestasis	Hepatobiliary Disorders	23
Development of an enzyme-linked immunosorbent assay for fecal bile acid.	2004	Masaru Matsumoto	Rinsho byori. The Japanese journal of clinical pathology	LCA	Feces Concentration	Case Control	Adults	Colon Cancer	Colorectal Cancer	115
Development of an enzyme-linked immunosorbent assay for fecal bile acid.	2004	Masaru Matsumoto	Rinsho byori. The Japanese journal of clinical pathology	DCA	Feces Concentration	Case Control	Adults	Colon Cancer	Colorectal Cancer	115
Serum bile acids in cholestasis of pregnancy.	1977	T Laatikainen	Obstetrics and gynecology	DCA	Blood Concentration	Case Control	Adults	Cholestasis Of Pregnancy	Hepatobiliary Disorders	126

Plasma sample based analysis of gastric cancer progression using targeted metabolomics.	2017	Sergio Lario	Scientific reports	Tryptamine	Blood Concentration	Case Control	Adults	Gastritis<br>Gastric Cancer<br>Gastric Adenocarcinoma	Digestive Disorders	80
Plasma sample based analysis of gastric cancer progression using targeted metabolomics.	2017	Sergio Lario	Scientific reports	Tryptamine	Blood Concentration	Case Control	Adults	Gastritis<br>Gastric Cancer<br>Gastric Adenocarcinoma	Cancer (Excluding Colorectal and Hepatobiliary Cancer)	80
Plasma sample based analysis of gastric cancer progression using targeted metabolomics.	2017	Sergio Lario	Scientific reports	PAG	Blood Concentration	Case Control	Adults	Gastritis<br>Gastric Cancer<br>Gastric Adenocarcinoma	Cancer (Excluding Colorectal and Hepatobiliary Cancer)	80
Plasma sample based analysis of gastric cancer progression using targeted metabolomics.	2017	Sergio Lario	Scientific reports	PAG	Blood Concentration	Case Control	Adults	Gastritis<br>Gastric Cancer<br>Gastric Adenocarcinoma	Digestive Disorders	80
Safety, pharmacokinetics and sialic acid production after oral administration of N-acetylmannosamine (ManNAc) to subjects with GNE myopathy.	2017	Xin Xu	Molecular genetics and metabolism	N-Acetylmannosamine	Oral Intake	Clinical Trial	Adults	GNE Myopathy	Other	22
Frequency-pulsed electron-capture gas-liquid chromatographic studies of chemical changes in sera of patients with schistosomiasis.	1985	J B Brooks	Journal of chromatography	Putrescine	Blood Concentration	Case Control	Adults	Schistosoma Mansoni Infections<br>S. Haematobium Infections	Other	57
Metabolomic Endotype of Asthma.	2015	Suzy A A Comhair	Journal of immunology (Baltimore, Md. : 1950)	GDCA	Blood Concentration	Case Control	Adults	Asthma	Respiratory Disorders	30
Serum bile acid profiling reflects enterohepatic detoxification state and intestinal barrier function in inflammatory bowel disease.	2009	Carsten Gnewuch	World journal of gastroenterology	LCA	Blood Concentration	Case Control	Adults	Crohn'S Disease<br>Ulcerative Colitis	Inflammatory Bowel Disease	668
Serum bile acid profiling reflects enterohepatic detoxification state and intestinal barrier function in inflammatory bowel disease.	2009	Carsten Gnewuch	World journal of gastroenterology	GDCA	Blood Concentration	Case Control	Adults	Crohn'S Disease<br>Ulcerative Colitis	Inflammatory Bowel Disease	668
Serum bile acid profiling reflects enterohepatic detoxification state and intestinal barrier function in inflammatory bowel disease.	2009	Carsten Gnewuch	World journal of gastroenterology	GLCA	Blood Concentration	Case Control	Adults	Crohn'S Disease<br>Ulcerative Colitis	Inflammatory Bowel Disease	668
Serum bile acid profiling reflects enterohepatic detoxification state and intestinal barrier function in inflammatory bowel disease.	2009	Carsten Gnewuch	World journal of gastroenterology	DCA	Blood Concentration	Case Control	Adults	Crohn'S Disease<br>Ulcerative Colitis	Inflammatory Bowel Disease	668

Sulfation and renal excretion of bile salts in patients with cirrhosis of the liver.	1975	A Stiehl	Gastroenterology	LCA	Urine Concentration	Case Control	Adults	Liver Cirrhosis	Hepatobiliary Disorders	19
Sulfation and renal excretion of bile salts in patients with cirrhosis of the liver.	1975	A Stiehl	Gastroenterology	DCA	Urine Concentration	Case Control	Adults	Liver Cirrhosis	Hepatobiliary Disorders	19
Serum bile acid profile in thyroid dysfunction and effect of medical treatment.	1987	T Kosuge	Clinical science (London, England : 1979)	LCA	Blood Concentration	Case Control	Adults	Thyroid Dysfunction	Other	57
Serum bile acid profile in thyroid dysfunction and effect of medical treatment.	1987	T Kosuge	Clinical science (London, England : 1979)	DCA	Blood Concentration	Case Control	Adults	Thyroid Dysfunction	Other	57
Bile Acid Deficiency in a Subgroup of Patients With Irritable Bowel Syndrome With Constipation Based on Biomarkers in Serum and Fecal Samples.	2018	Priya Vijayvargiya	Clinical gastroenterology and hepatology : the official clinical practice journal of the American Gastroenterological Association	DCA	Feces Concentration	Case Control	Adults	Irritable Bowel Syndrome	Digestive Disorders	229
Diminished bile acids excretion is a risk factor for coronary artery disease: 20-year follow up and long-term outcome.	2018	Gideon Charach	Therapeutic advances in gastroenterology	LCA	Feces Concentration	Case Control	Adults	Coronary Artery Disease	Cardiovascular Disorders	67
Diminished bile acids excretion is a risk factor for coronary artery disease: 20-year follow up and long-term outcome.	2018	Gideon Charach	Therapeutic advances in gastroenterology	DCA	Feces Concentration	Case Control	Adults	Coronary Artery Disease	Cardiovascular Disorders	67
Attenuation of bile acid-mediated FXR and PXR activation in patients with Crohn's disease.	2020	Aze Wilson	Scientific reports	LCA	Blood Concentration	Case Control	Adults	Crohn's Disease	Inflammatory Bowel Disease	145
Further evidence for the use of polyamines as biochemical markers for malignant tumors.	1982	Y Horn	Cancer research	Putrescine	Urine Concentration	Case Control	Adults	Bladder Cancer<br>Breast Cancer<br>Stomach Cancer<br>Colon Cancer<br>Rectum Cancer<br>Prostate Cancer<br>Testes Cancer<br>Female Genital Cancer<br>Lung Cancer<br>Head And Neck Cancer<br>Miscellaneous Cancer<br>Metastatic Unknowncarcinoma Of Origin	Colorectal Cancer	192
Further evidence for the use of polyamines as biochemical markers for malignant tumors.	1982	Y Horn	Cancer research	Putrescine	Urine Concentration	Case Control	Adults	Bladder Cancer<br>Breast Cancer<br>Stomach Cancer<br>Colon Cancer<br>Rectum Cancer<br>Prostate Cancer<br>Testes Cancer	Cancer (Excluding Colorectal and Hepatobiliary Cancer)	192

								Female Genital Cancer<br>Lung Cancer<br>Head And Neck Cancer<br>Miscellaneous Cancer<br>Metastatic<br>Unknowncarcinoma<br>Of Origin		
A preliminary study of polyamines in the bone-marrow plasma of adult patients with leukemia.	1980	K Nishioka	Clinica chimica acta; international journal of clinical chemistry	Putrescine	Blood Concentration	Case Control	Adults	Acute Leukemia	Cancer (Excluding Colorectal and Hepatobiliary Cancer)	28
Biological markers in breast carcinoma. I. Incidence of abnormalities of CEA, HCG, three polyamines, and three minor nucleosides.	1975	D C Tormey	Cancer	Putrescine	Urine Concentration	Case Control	Adults	Breast Cancer	Cancer (Excluding Colorectal and Hepatobiliary Cancer)	108
Polyamines in colorectal cancer--a clinical and experimental approach.	1983	R Carachi	Journal of clinical pathology	Putrescine	Urine Concentration	Case Control	Adults	Colorectal Cancer	Colorectal Cancer	20
Elevation of serum polyamines in malignant lymphomas and acute myeloid leukemia.	1980	A V Hospattankar	International journal of cancer	Putrescine	Blood Concentration	Case Control	Adults	Hodgkin's Disease	Cancer (Excluding Colorectal and Hepatobiliary Cancer)	6
Presence of bile acid metabolites in serum, urine, and faeces in cirrhosis.	1992	G Jönsson	Scandinavian journal of clinical and laboratory investigation	DCA	Urine Concentration	Case Control	Adults	Liver Cirrhosis	Hepatobiliary Disorders	10
Presence of bile acid metabolites in serum, urine, and faeces in cirrhosis.	1992	G Jönsson	Scandinavian journal of clinical and laboratory investigation	LCA	Blood Concentration	Case Control	Adults	Liver Cirrhosis	Hepatobiliary Disorders	10
Presence of bile acid metabolites in serum, urine, and faeces in cirrhosis.	1992	G Jönsson	Scandinavian journal of clinical and laboratory investigation	LCA	Feces Concentration	Case Control	Adults	Liver Cirrhosis	Hepatobiliary Disorders	10
Presence of bile acid metabolites in serum, urine, and faeces in cirrhosis.	1992	G Jönsson	Scandinavian journal of clinical and laboratory investigation	DCA	Blood Concentration	Case Control	Adults	Liver Cirrhosis	Hepatobiliary Disorders	10
Presence of bile acid metabolites in serum, urine, and faeces in cirrhosis.	1992	G Jönsson	Scandinavian journal of clinical and laboratory investigation	DCA	Feces Concentration	Case Control	Adults	Liver Cirrhosis	Hepatobiliary Disorders	10
Presence of bile acid metabolites in serum, urine, and faeces in cirrhosis.	1992	G Jönsson	Scandinavian journal of clinical and laboratory investigation	LCA	Urine Concentration	Case Control	Adults	Liver Cirrhosis	Hepatobiliary Disorders	10
Bile acids in serum and bile of patients with cholesterol gallstone.	1998	Tian-Quan Han	World journal of gastroenterology	DCA	Blood Concentration	Case Control	Adults	Cholesterol Gallstone	Hepatobiliary Disorders	407
Prominent accumulation in hemodialysis patients of solutes normally cleared by tubular secretion.	2014	Tammy L Sirich	Journal of the American Society of Nephrology : JASN	PAG	Blood Concentration	Case Control	Adults	Hemodialysis	Renal Disorders	41

Faecal unconjugated bile acids in patients with colorectal cancer or polyps.	1992	C H Imray	Gut	DCA	Feces Concentration	Case Control	Adults	Colorectal Cancer Colorectal Polyps	Digestive Disorders	33
Faecal unconjugated bile acids in patients with colorectal cancer or polyps.	1992	C H Imray	Gut	DCA	Feces Concentration	Case Control	Adults	Colorectal Cancer Colorectal Polyps	Colorectal Cancer	33
Faecal unconjugated bile acids in patients with colorectal cancer or polyps.	1992	C H Imray	Gut	LCA	Feces Concentration	Case Control	Adults	Colorectal Cancer Colorectal Polyps	Colorectal Cancer	33
Faecal unconjugated bile acids in patients with colorectal cancer or polyps.	1992	C H Imray	Gut	LCA	Feces Concentration	Case Control	Adults	Colorectal Cancer Colorectal Polyps	Digestive Disorders	33
Polyamine concentrations in pancreatic tissue, serum, and urine of patients with pancreatic cancer.	1990	C Löser	Pancreas	Putrescine	Blood Concentration	Case Control	Adults	Pancreatic Cancer Nonmalignant Gastrointestinal Diseases	Cancer (Excluding Colorectal and Hepatobiliary Cancer)	90
Polyamine concentrations in pancreatic tissue, serum, and urine of patients with pancreatic cancer.	1990	C Löser	Pancreas	Putrescine	Urine Concentration	Case Control	Adults	Pancreatic Cancer Nonmalignant Gastrointestinal Diseases	Cancer (Excluding Colorectal and Hepatobiliary Cancer)	90
Polyamine concentrations in pancreatic tissue, serum, and urine of patients with pancreatic cancer.	1990	C Löser	Pancreas	Putrescine	Urine Concentration	Case Control	Adults	Pancreatic Cancer Nonmalignant Gastrointestinal Diseases	Digestive Disorders	90
Polyamine concentrations in pancreatic tissue, serum, and urine of patients with pancreatic cancer.	1990	C Löser	Pancreas	Putrescine	Blood Concentration	Case Control	Adults	Pancreatic Cancer Nonmalignant Gastrointestinal Diseases	Digestive Disorders	90
Serum bile acids in the diagnosis of hepatobiliary disease.	1977	C R Pennington	Gut	DCA	Blood Concentration	Case Control	Adults	Hepatobiliary Disorders	Hepatobiliary Disorders	87
Bile acid concentrations, cytotoxicity, and pH of fecal water from patients with colorectal adenomas.	1999	T M de Kok	Digestive diseases and sciences	LCA	Feces Concentration	Case Control	Adults	Colorectal Adenoma	Digestive Disorders	64
Bile acid concentrations, cytotoxicity, and pH of fecal water from patients with colorectal adenomas.	1999	T M de Kok	Digestive diseases and sciences	DCA	Feces Concentration	Case Control	Adults	Colorectal Adenoma	Digestive Disorders	64
SERUM METABOLOMIC PATTERNS IN PATIENTS WITH AUTOIMMUNE THYROID DISEASE.	2020	Jia Liu	Endocrine practice : official journal of the American College of Endocrinology and the American Association of Clinical Endocrinologists	GDCA	Blood Concentration	Case Control	Adults	Hyperthyroidism Hypothyroidism	Other	140
SERUM METABOLOMIC PATTERNS IN PATIENTS WITH AUTOIMMUNE THYROID DISEASE.	2020	Jia Liu	Endocrine practice : official journal of the American College	DCA	Blood Concentration	Case Control	Adults	Hyperthyroidism Hypothyroidism	Other	140

			of Endocrinology and the American Association of Clinical Endocrinologists							
Quantification of biogenic amines in human plasma based on the derivatization with N-hydroxy-succinimidyl fluorescein-O-acetate by high-performance liquid chromatography.	2008	Ying-Hua Deng	Journal of separation science	Putrescine	Blood Concentration	Case Control	Adults	Lung Cancer<br>Rectum Cancer<br>Cervical cancer<br>B-cell lymphoma	Cancer (Excluding Colorectal and Hepatobiliary Cancer)	7
Quantification of biogenic amines in human plasma based on the derivatization with N-hydroxy-succinimidyl fluorescein-O-acetate by high-performance liquid chromatography.	2008	Ying-Hua Deng	Journal of separation science	Putrescine	Blood Concentration	Case Control	Adults	Lung Cancer<br>Rectum Cancer<br>Cervical cancer<br>B-cell lymphoma	Colorectal Cancer	7
Comparison of fecal microbiota and polyamine concentration in adult patients with intractable atopic dermatitis and healthy adults.	2007	Mitsuharu Matsumoto	Microbiology and immunology	Putrescine	Feces Concentration	Case Control	Adults	Intractable Atopic Dermatitis	Other	24
Analysis of polyamines as carbamoyl derivatives in urine and serum by liquid chromatography-tandem mass spectrometry.	2008	Jeong Ah Byun	Biomedical chromatography : BMC	Putrescine	Blood Concentration	Case Control	Adults	Breast Cancer	Cancer (Excluding Colorectal and Hepatobiliary Cancer)	60
Analysis of polyamines as carbamoyl derivatives in urine and serum by liquid chromatography-tandem mass spectrometry.	2008	Jeong Ah Byun	Biomedical chromatography : BMC	Putrescine	Urine Concentration	Case Control	Adults	Breast Cancer	Cancer (Excluding Colorectal and Hepatobiliary Cancer)	60
Serum metabolomic profiling in acute alcoholic hepatitis identifies multiple dysregulated pathways.	2014	Vikrant Rachakonda	PloS one	GDCA	Blood Concentration	Case Control	Adults	Acute Alcoholic Hepatitis<br>Alcoholic Cirrhosis	Hepatobiliary Disorders	50
Serum metabolomic profiling in acute alcoholic hepatitis identifies multiple dysregulated pathways.	2014	Vikrant Rachakonda	PloS one	DCA	Blood Concentration	Case Control	Adults	Acute Alcoholic Hepatitis<br>Alcoholic Cirrhosis	Hepatobiliary Disorders	50
A prospective study of serum bile acid concentrations and colorectal cancer risk in post-menopausal women on the island of Guernsey.	2002	V Costarelli	British journal of cancer	DCA	Blood Concentration	Case Control	Adults	Colorectal Cancer	Colorectal Cancer	3680
A prospective study of serum bile acid concentrations and colorectal cancer risk in post-menopausal women on the island of Guernsey.	2002	V Costarelli	British journal of cancer	LCA	Blood Concentration	Case Control	Adults	Colorectal Cancer	Colorectal Cancer	3680

Aberrant lipid metabolism in hepatocellular carcinoma revealed by plasma metabolomics and lipid profiling.	2011	Andrew D Patterson	Cancer research	GDCA	Blood Concentration	Case Control	Adults	Hepatocellular Carcinoma	Hepatobiliary Cancer	26
Determination of polyamines in human urine by precolumn derivatization with benzoyl chloride and high-performance liquid chromatography coupled with Q-time-of-flight mass spectrometry.	2011	Ran Liu	Talanta	Putrescine	Urine Concentration	Case Control	Adults	Cancer	Cancer (Excluding Colorectal and Hepatobiliary Cancer)	25
Bile Acid Changes Associated With Liver Fibrosis and Steatosis in the Mexican-American Population of South Texas.	2020	Suet Ying Kwan	Hepatology communications	LCA	Blood Concentration	Case Control	Adults	Liver Fibrosis<br>Liver Steatosis	Hepatobiliary Disorders	390
Bile Acid Changes Associated With Liver Fibrosis and Steatosis in the Mexican-American Population of South Texas.	2020	Suet Ying Kwan	Hepatology communications	DCA	Blood Concentration	Case Control	Adults	Liver Fibrosis<br>Liver Steatosis	Hepatobiliary Disorders	390
Fasting plasma chenodeoxycholic acid and cholic acid concentrations are inversely correlated with insulin sensitivity in adults.	2011	Bertrand Cariou	Nutrition & metabolism	DCA	Blood Concentration	Case Control	Adults	Diabetes<br>Insulin Sensitivity<br>Non-Diabetic<br>Abdominally Obesity	Metabolic Disorders	56
Fasting plasma chenodeoxycholic acid and cholic acid concentrations are inversely correlated with insulin sensitivity in adults.	2011	Bertrand Cariou	Nutrition & metabolism	DCA	Blood Concentration	Case Control	Adults	Diabetes<br>Insulin Sensitivity<br>Non-Diabetic<br>Abdominally Obesity	Diabetes/Impaired Glucose Metabolism	56
Altered urinary profiles of polyamines and endogenous steroids in patients with benign cervical disease and cervical cancer.	2003	Seon Hwa Lee	Cancer letters	Putrescine	Urine Concentration	Case Control	Adults	Cervical Cancer	Cancer (Excluding Colorectal and Hepatobiliary Cancer)	61
Age-related abnormalities of circulating polyamines and diamine oxidase activity in cystic fibrosis heterozygotes and homozygotes.	1980	S B Baylin	Pediatric research	Putrescine	Blood Concentration	Case Control	Adults	Cystic Fibrosis	Other	129
Serum bile acids and ursodeoxycholic acid treatment in cystic fibrosis-related liver disease.	1996	S M O'Brien	European journal of gastroenterology & hepatology	DCA	Blood Concentration	Case Control	Adults	Cystic Fibrosis-Related Liver Disease<br>Cystic Fibrosis without Liver Disease	Hepatobiliary Disorders	43
Serum bile acids and ursodeoxycholic acid treatment in cystic fibrosis-related liver disease.	1996	S M O'Brien	European journal of gastroenterology & hepatology	DCA	Blood Concentration	Case Control	Adults	Cystic Fibrosis-Related Liver Disease<br>Cystic Fibrosis without Liver Disease	Other	43
Urine PAG determination in patients with hyperphenylalaninemia	2021	Andrade, F.	Journal of Clinical Medicine	PAG	Urine Concentration	Case Control	Adults	Mild Hyperphenylalaninemi	Metabolic Disorders	68

								a Phenylketonuria		
Serum metabolic signatures of chronic limb-threatening ischemia in patients with peripheral artery disease	2020	Azab, S. M.	Journal of Clinical Medicine	PAG	Blood Concentration	Case Control	Adults	Peripheral Artery Disease	Cardiovascular Disorders	58
Metabolomic biomarkers in the diagnosis of non-alcoholic fatty liver disease	2019	Chashmniam, S.	Hepatitis Monthly	DCA	Blood Concentration	Case Control	Adults	Non-Alcoholic Fatty Liver Disease	Hepatobiliary Disorders	73
Plasma-metabolite-based machine learning is a promising diagnostic approach for esophageal squamous cell carcinoma investigation	2021	Chen, Z.	Journal of Pharmaceutical Analysis	DCA	Blood Concentration	Case Control	Adults	Esophageal Squamous Cell Carcinoma	Cancer (Excluding Colorectal and Hepatobiliary Cancer)	140
Dietary polyamines intake and risk of colorectal cancer: A case-control study	2020	Huang, C. Y.	Nutrients	Putrescine	Oral Intake	Case Control	Adults	Colorectal Cancer	Colorectal Cancer	5040
Neuroendocrine neoplasms: Identification of novel metabolic circuits of potential diagnostic utility	2021	Jiménez, B.	Cancers	PAG	Urine Concentration	Case Control	Adults	Neuroendocrine Neoplasms	Cancer (Excluding Colorectal and Hepatobiliary Cancer)	54
Altered plasma levels of arginine metabolites in depression.	2020	Arisoy Ozden	Journal of psychiatric research	Putrescine	Blood Concentration	Case Control	Adults	Major Depressive Disorder	Mental Disorders	104
Decreased plasmatic spermidine and increased spermine in mild cognitive impairment and alzheimer's disease patients	2019	Joaquim, H. P. G.	Revista de Psiquiatria Clinica	Putrescine	Blood Concentration	Case Control	Adults	Mild Cognitive Impairment Alzheimer'S Disease	Neurological Disorders	79
Glycodeoxycholic acid levels as prognostic biomarker in acetaminophen-induced acute liver failure patients.	2014	Benjamin L Woolbright	Toxicological sciences : an official journal of the Society of Toxicology	GDCA	Blood Concentration	Case Control	Adults	Acetaminophen-Induced Acute Liver Failure	Hepatobiliary Disorders	90
Microbiomics, Metabolomics, Predicted Metagenomics, and Hepatic Steatosis in a Population-Based Study of 1,355 Adults.	2021	Louise J M Alferink	Hepatology (Baltimore, Md.)	DCA	Blood Concentration	Case Control	Adults	Hepatic Steatosis	Hepatobiliary Disorders	1355
Characteristic of metabolic status in heart failure and its impact in outcome perspective	2020	Tang, H. Y.	Metabolites	PAG	Blood Concentration	Case Control	Adults	Heart Failure	Cardiovascular Disorders	61
Compositional and functional adaptations of intestinal microbiota and related metabolites in ckd patients receiving dietary protein restriction	2020	Wu, I. W.	Nutrients	d-Alanine	Blood Concentration	Case Control	Adults	Chronic Kidney Disorders	Renal Disorders	77
Altered gut microbial metabolites in amnestic mild cognitive impairment and alzheimer's disease: Signals in host–microbe interplay	2021	Wu, L.	Nutrients	GDCA	Feces Concentration	Case Control	Adults	Amnestic Mild Cognitive Impairment Alzheimer's Disease	Neurological Disorders	77

Altered gut microbial metabolites in amnestic mild cognitive impairment and alzheimer’s disease: Signals in host–microbe interplay	2021	Wu, L.	Nutrients	DCA	Feces Concentration	Case Control	Adults	Amnestic Mild Cognitive Impairment Alzheimer's Disease	Neurological Disorders	77
Altered gut microbial metabolites in amnestic mild cognitive impairment and alzheimer’s disease: Signals in host–microbe interplay	2021	Wu, L.	Nutrients	LCA	Feces Concentration	Case Control	Adults	Amnestic Mild Cognitive Impairment Alzheimer's Disease	Neurological Disorders	77
Altered gut microbial metabolites in amnestic mild cognitive impairment and alzheimer’s disease: Signals in host–microbe interplay	2021	Wu, L.	Nutrients	GLCA	Feces Concentration	Case Control	Adults	Amnestic Mild Cognitive Impairment Alzheimer's Disease	Neurological Disorders	77
Reduced bile acid excretion is an independent risk factor for stroke and mortality: A prospective follow-up study.	2020	Gideon Charach	Atherosclerosis	LCA	Feces Concentration	Cohort Study	Adults	Stroke Coronary Artery Disease	Neurological Disorders	103
Reduced bile acid excretion is an independent risk factor for stroke and mortality: A prospective follow-up study.	2020	Gideon Charach	Atherosclerosis	DCA	Feces Concentration	Cohort Study	Adults	Stroke Coronary Artery Disease	Cardiovascular Disorders	103
Reduced bile acid excretion is an independent risk factor for stroke and mortality: A prospective follow-up study.	2020	Gideon Charach	Atherosclerosis	LCA	Feces Concentration	Cohort Study	Adults	Stroke Coronary Artery Disease	Cardiovascular Disorders	103
Reduced bile acid excretion is an independent risk factor for stroke and mortality: A prospective follow-up study.	2020	Gideon Charach	Atherosclerosis	DCA	Feces Concentration	Cohort Study	Adults	Stroke Coronary Artery Disease	Neurological Disorders	103
Untargeted metabolomics approach (UPLC-Q-TOF-MS) explores the biomarkers of serum and urine in overweight/obese young men	2018	Yu, H. T.	Asia Pacific journal of clinical nutrition	PAG	Urine Concentration	Case Control	Adults	Obesity	Metabolic Disorders	71
Circulating amino acids and amino acid-related metabolites and risk of breast cancer among predominantly premenopausal women.	2021	Oana A Zeleznik	NPJ breast cancer	PAG	Blood Concentration	Case Control	Adults	Breast Cancer	Cancer (Excluding Colorectal and Hepatobiliary Cancer)	2114
Metabolomic study of a diagnostic model for the metabolites of stool fat.	2013	Choong Hwan Lee	The Korean journal of gastroenterology = Taehan Sohwagi Hakhoe chi	LCA	Feces Concentration	Case Control	Adults	Bowel Habit Change	Digestive Disorders	52
Gut microbial diversity is associated with lower arterial stiffness in women.	2018	Cristina Menni	European heart journal	PAG	Blood Concentration	Cross-Sectional	Adults	Carotid-femoral Pulse Wave Velocity	Cardiovascular Disorders	617
Malodorous biogenic amines in Escherichia coli-caused urinary tract infections in women-a metabolomics approach.	2020	Scarlett Puebla-Barragan	Scientific reports	Putrescine	Urine Concentration	Case Control	Adults	Urinary Tract Infections	Other	25
Holistic metabonomic profiling of urine affords potential early diagnosis for bladder and kidney cancers	2013	Huang, Z.	Metabolomics	PAG	Urine Concentration	Case Control	Adults	Bladder Cancer Kidney Cancer	Cancer (Excluding Colorectal and	68

									Hepatobiliary Cancer)	
The gut microbiota-related metabolite PAG associates with increased risk of incident coronary artery disease.	2020	Filip Ottosson	Journal of hypertension	PAG	Blood Concentration	Cohort Study	Adults	Coronary Artery Disease	Cardiovascular Disorders	4242
Free Levels of Selected Organic Solutes and Cardiovascular Morbidity and Mortality in Hemodialysis Patients: Results from the Retained Organic Solutes and Clinical Outcomes (ROSCO) Investigators.	2015	Tariq Shafi	PloS one	PAG	Blood Concentration	Cohort Study	Adults	Cardiovascular Mortality And Morbidity Dialysis	Cardiovascular Disorders	394
Free Levels of Selected Organic Solutes and Cardiovascular Morbidity and Mortality in Hemodialysis Patients: Results from the Retained Organic Solutes and Clinical Outcomes (ROSCO) Investigators.	2015	Tariq Shafi	PloS one	PAG	Blood Concentration	Cohort Study	Adults	Cardiovascular Mortality And Morbidity Dialysis	Renal Disorders	394
Fecal Metabolites Were Altered, Identified as Biomarkers and Correlated With Disease Activity in Patients With Systemic Lupus Erythematosus in a GC-MS-Based Metabolomics Study.	2020	Ren Yan	Frontiers in immunology	DCA	Feces Concentration	Case Control	Adults	Systemic Lupus Erythematosus	Other	59
Fecal Metabolites Were Altered, Identified as Biomarkers and Correlated With Disease Activity in Patients With Systemic Lupus Erythematosus in a GC-MS-Based Metabolomics Study.	2020	Ren Yan	Frontiers in immunology	Putrescine	Feces Concentration	Case Control	Adults	Systemic Lupus Erythematosus	Other	59
Increased mortality of acute respiratory distress syndrome was associated with high levels of plasma phenylalanine.	2020	Jing Xu	Respiratory research	PAG	Blood Concentration	Case Control	Adults	Acute Respiratory Distress Syndrome	Respiratory Disorders	70
Serum metabolomics in oral leukoplakia and oral squamous cell carcinoma	2017	Sridharan, G.	Journal of Cancer Research and Therapeutics	Putrescine	Blood Concentration	Case Control	Adults	Oral Leukoplakia Oral Squamous Cell Carcinoma	Cancer (Excluding Colorectal and Hepatobiliary Cancer)	62
Analysis of eight bile acids in urine of gastric cancer patients based on covalent organic framework enrichment coupled with liquid chromatography-tandem mass spectrometry.	2021	Jinxiu Lyu	Journal of chromatography. A	LCA	Urine Concentration	Case Control	Adults	Gastric Cancer	Cancer (Excluding Colorectal and Hepatobiliary Cancer)	108
Analysis of eight bile acids in urine of gastric cancer patients based on covalent organic	2021	Jinxiu Lyu	Journal of chromatography. A	DCA	Urine Concentration	Case Control	Adults	Gastric Cancer	Cancer (Excluding Colorectal and	108

framework enrichment coupled with liquid chromatography-tandem mass spectrometry.									Hepatobiliary Cancer)	
Safety and efficacy of N-acetylmannosamine (ManNAc) in patients with GNE myopathy: an open-label phase 2 study.	2021	Nuria Carrillo	Genetics in medicine : official journal of the American College of Medical Genetics	N-Acetylmannosamine	Oral Intake	Clinical Trial	Adults	GNE Myopathy	Other	12
Simultaneous HPLC determination of free, conjugated, and sulfated bile acids	1993	Nichols, J. H.	Journal of Liquid Chromatography	DCA	Urine Concentration	Case Control	Adults	Hepatitis And Biliary Obstruction	Hepatobiliary Disorders	8
Simultaneous HPLC determination of free, conjugated, and sulfated bile acids	1993	Nichols, J. H.	Journal of Liquid Chromatography	GDCA	Urine Concentration	Case Control	Adults	Hepatitis And Biliary Obstruction	Hepatobiliary Disorders	8
Simultaneous HPLC determination of free, conjugated, and sulfated bile acids	1993	Nichols, J. H.	Journal of Liquid Chromatography	LCA	Urine Concentration	Case Control	Adults	Hepatitis And Biliary Obstruction	Hepatobiliary Disorders	8
Simultaneous HPLC determination of free, conjugated, and sulfated bile acids	1993	Nichols, J. H.	Journal of Liquid Chromatography	GLCA	Urine Concentration	Case Control	Adults	Hepatitis And Biliary Obstruction	Hepatobiliary Disorders	8
Increase of deoxycholate in supersaturated bile of patients with cholesterol gallstone disease and its correlation with de novo syntheses of cholesterol and bile acids in liver, gallbladder emptying, and small intestinal transit	1995	Shoda, J.	Hepatology	DCA	Blood Concentration	Case Control	Adults	Cholesterol Stones Brown Pigment Stones Gallbladder Stones	Hepatobiliary Disorders	100
Gut microbiome composition and serum metabolome profile among individuals with spinal cord injury and normal glucose tolerance or prediabetes/type 2 diabetes.	2021	Jia Li	Archives of physical medicine and rehabilitation	PAG	Blood Concentration	Case Control	Adults	Prediabetes in Spinal Cord Injury	Other	25
Gut microbiome composition and serum metabolome profile among individuals with spinal cord injury and normal glucose tolerance or prediabetes/type 2 diabetes.	2021	Jia Li	Archives of physical medicine and rehabilitation	PAG	Blood Concentration	Case Control	Adults	Prediabetes in Spinal Cord Injury	Diabetes/Impaired Glucose Metabolism	25
Tryptamine levels are low in plasma of chronic migraine and chronic tension-type headache.	2014	Giovanni D'Andrea	Neurological sciences : official journal of the Italian Neurological Society and of the Italian Society of Clinical Neurophysiology	Tryptamine	Blood Concentration	Case Control	Adults	Chronic Migraine Chronic Tension-Type Headache	Neurological Disorders	123
Type 2 Diabetes Is Associated with a Different Pattern of Serum Polyamines: A Case⁻Control Study from the PREDIMED-Plus Trial.	2019	Jose C Fernandez-Garcia	Journal of clinical medicine	Putrescine	Blood Concentration	Case Control	Adults	Type 2 Diabetes Metabolic Syndrome	Diabetes/Impaired Glucose Metabolism	114
Type 2 Diabetes Is Associated with a Different Pattern of Serum Polyamines: A Case⁻Control Study from the PREDIMED-Plus Trial.	2019	Jose C Fernandez-Garcia	Journal of clinical medicine	Putrescine	Blood Concentration	Case Control	Adults	Type 2 Diabetes Metabolic Syndrome	Metabolic Disorders	114
Histopathologic changes in serum bile acid fractions in pressure ulcer patients	2005	Kanoh, M.	Hepato-Gastroenterology	DCA	Blood Concentration	Case Control	Adults	Pressure Ulcers	Dermotological Disorders	50
Histopathologic changes in serum bile acid fractions in pressure ulcer patients	2005	Kanoh, M.	Hepato-Gastroenterology	LCA	Blood Concentration	Case Control	Adults	Pressure Ulcers	Dermotological Disorders	50

Pressurized CEC coupled with QTOF-MS for urinary metabolomics.	2014	Qian Wu	Electrophoresis	PAG	Urine Concentration	Case Control	Adults	Lung Cancer	Cancer (Excluding Colorectal and Hepatobiliary Cancer)	24
Metabolomic study of lipids in serum for biomarker discovery in Alzheimer's disease using direct infusion mass spectrometry.	2014	R González-Domínguez	Journal of pharmaceutical and biomedical analysis	Putrescine	Blood Concentration	Case Control	Adults	Alzheimer's Disease	Neurological Disorders	40
Development and validation of a rapid, selective, and sensitive LC-MS/MS method for simultaneous determination of D- and L-amino acids in human serum: application to the study of hepatocellular carcinoma.	2018	Minlu Han	Analytical and bioanalytical chemistry	d-Alanine	Blood Concentration	Case Control	Adults	Hepatocellular Carcinoma	Hepatobiliary Cancer	60
Metabonomics study of intestinal fistulas based on ultraperformance liquid chromatography coupled with Q-TOF mass spectrometry (UPLC/Q-TOF MS)	2006	Yin, P.	Journal of Proteome Research	GDCA	Blood Concentration	Case Control	Adults	Intestinal Fistula	Digestive Disorders	57
Simultaneous determination of twelve biogenic amines in human urine as potential biomarkers of inflammatory bowel diseases by capillary electrophoresis - tandem mass spectrometry.	2020	Katarína Maráková	Journal of pharmaceutical and biomedical analysis	Putrescine	Urine Concentration	Case Control	Adults	Inflammatory Bowel Diseases	Inflammatory Bowel Disease	19
Simultaneous determination of twelve biogenic amines in human urine as potential biomarkers of inflammatory bowel diseases by capillary electrophoresis - tandem mass spectrometry.	2020	Katarína Maráková	Journal of pharmaceutical and biomedical analysis	Tryptamine	Urine Concentration	Case Control	Adults	Inflammatory Bowel Diseases	Inflammatory Bowel Disease	19
A Cardiovascular Disease-Linked Gut Microbial Metabolite Acts via Adrenergic Receptors.	2020	Ina Nemet	Cell	PAG	Blood Concentration	Cohort Study	Adults	Cardiovascular Disease Incident Major Adverse Cardiovascular Events	Cardiovascular Disorders	5162
Investigation of novel metabolites potentially involved in the pathogenesis of coronary heart disease using a UHPLC-QTOF/MS-based metabolomics approach.	2017	Yiping Li	Scientific reports	LCA	Blood Concentration	Case Control	Adults	Coronary Heart Disease	Cardiovascular Disorders	300
Metabolite quantification of faecal extracts from colorectal cancer patients and healthy controls.	2018	Gwénaëlle Le Gall	Oncotarget	DCA	Feces Concentration	Case Control	Adults	Colorectal Cancer	Colorectal Cancer	40
Rapid and sensitive determination of five amine biomarkers in plasma samples from stroke patients by MEKC with precolumn derivatization	2012	Peng, L.	Chromatographia	Putrescine	Blood Concentration	Case Control	Adults	Stroke	Cardiovascular Disorders	106

Conjugated and unconjufated serum bile acid levels in patients with ttepatobiliary diseases	1969	Makino, I.	Gastroenterology (New York, N.Y. 1943)	DCA	Blood Concentration	Case Control	Adults	Hepatobiliary Disorders	Hepatobiliary Disorders	101
Measurement of putrescine, spermidine, and spermine in physiological fluids by use of an amino acid analyzer	1973	Marton, L. J.	Clinical Chemistry	Putrescine	Blood Concentration	Case Control	Adults	Mediastinal Choriocarcinoma<br>Pancreatic Carcinoma<br>Malignant Teratoma<br>Breast Cancer<br>Lymphoma<br>Hodgkin's Disease<br>Anaplastic Carcinoma<br>Lung Cancer<br>Testicular Embryonal Carcinoma<br>Acute Nonlymphocytic Leukemia	Cancer (Excluding Colorectal and Hepatobiliary Cancer)	28
Measurement of putrescine, spermidine, and spermine in physiological fluids by use of an amino acid analyzer	1973	Marton, L. J.	Clinical Chemistry	Putrescine	Urine Concentration	Case Control	Adults	Mediastinal Choriocarcinoma<br>Pancreatic Carcinoma<br>Malignant Teratoma<br>Breast Cancer<br>Lymphoma<br>Hodgkin's Disease<br>Anaplastic Carcinoma<br>Lung Cancer<br>Testicular Embryonal Carcinoma<br>Acute Nonlymphocytic Leukemia	Cancer (Excluding Colorectal and Hepatobiliary Cancer)	28
Simple, sensitive assay of polyamines by high-performance liquid chromatography with electrochemical detection after post-column reaction with immobilized polyamine oxidase	1989	Maruta, K.	Clinical Chemistry	Putrescine	Urine Concentration	Case Control	Adults	Blood Cancer<br>Solid Cancer	Cancer (Excluding Colorectal and Hepatobiliary Cancer)	170
Platelet monoamine oxidase activity and substrate preferences in schizophrenic patients	1974	Meltzer, H. Y.	RES.COMMUN.CHEM.PATH.PHA RMACOL.	Tryptamine	Blood Concentration	Case Control	Adults	Schizophrenia	Neurological Disorders	37
Dietary polyamine intake and colorectal cancer risk in postmenopausal women.	2015	Ashley J Vargas	The American journal of clinical nutrition	Putrescine	Oral Intake	Cohort Study	Adults	Colorectal Cancer	Colorectal Cancer	87602
Narcolepsy and idiopathic hypersomnia: Biogenic amines and related compounds in CSF	1982	Montplaisir, J.	Neurology	Tryptamine	Blood Concentration	Case Control	Adults	Narcolepsy<br>Diopathic Hypersomnia	Neurological Disorders	33
Diet, fecal bile acids, and neutral sterols in carcinoma of the colon	1979	Moskovitz, M.	Digestive Diseases and Sciences	LCA	Feces Concentration	Case Control	Adults	Colonic Adenocarcinoma	Colorectal Cancer	54

Diet, fecal bile acids, and neutral sterols in carcinoma of the colon	1979	Moskovitz, M.	Digestive Diseases and Sciences	LCA	Feces Concentration	Case Control	Adults	Colonic Adenocarcinoma	Cancer (Excluding Colorectal and Hepatobiliary Cancer)	54
Diet, fecal bile acids, and neutral sterols in carcinoma of the colon	1979	Moskovitz, M.	Digestive Diseases and Sciences	DCA	Feces Concentration	Case Control	Adults	Colonic Adenocarcinoma	Colorectal Cancer	54
Diet, fecal bile acids, and neutral sterols in carcinoma of the colon	1979	Moskovitz, M.	Digestive Diseases and Sciences	DCA	Feces Concentration	Case Control	Adults	Colonic Adenocarcinoma	Cancer (Excluding Colorectal and Hepatobiliary Cancer)	54
Elevation of putrescine and spermidine in sera of patients with solid tumors	1974	Nishioka, K.	Clinica Chimica Acta	Putrescine	Blood Concentration	Case Control	Adults	Colon Cancer<br>Rectum Cancer<br>Sarcoma Cancer<br>Melanoma Cancer<br>Breast Cancer<br>Hodgkin’s Cancer<br>Kidney Cancer	Colorectal Cancer	50
Elevation of putrescine and spermidine in sera of patients with solid tumors	1974	Nishioka, K.	Clinica Chimica Acta	Putrescine	Blood Concentration	Case Control	Adults	Colon Cancer<br>Rectum Cancer<br>Sarcoma Cancer<br>Melanoma Cancer<br>Breast Cancer<br>Hodgkin’s Cancer<br>Kidney Cancer	Cancer (Excluding Colorectal and Hepatobiliary Cancer)	50
Serum bile acids in patients with viral hepatitis	1978	Pennington, C. R.	Scandinavian Journal of Gastroenterology	DCA	Blood Concentration	Case Control	Adults	Viral Hepatitis	Hepatobiliary Disorders	18
Plasma Metabolome and Lipidome Associations with Type 2 Diabetes and Diabetic Nephropathy.	2021	Yan Ming Tan	Metabolites	PAG	Blood Concentration	Case Control	Adults	Type 2 Diabetes<br>Diabetic Nephropathy	Diabetes/Impaire d Glucose Metabolism	90
Plasma Metabolome and Lipidome Associations with Type 2 Diabetes and Diabetic Nephropathy.	2021	Yan Ming Tan	Metabolites	PAG	Blood Concentration	Case Control	Adults	Type 2 Diabetes<br>Diabetic Nephropathy	Renal Disorders	90
Aromatic amines and Parkinson's disease	1969	Smith, I.	Nature	Tryptamine	Urine Concentration	Case Control	Adults	Parkinson'S Disease	Neurological Disorders	34
Catabolism of cholesterol in hypercholesterolemia and its relationship to plasma triglycerides	1973	Sodhi, H. S.	Clinica Chimica Acta	DCA	Feces Concentration	Case Control	Adults	Hypercholesterolemia<br>Hypertriglyceridemia	Metabolic Disorders	16

Catabolism of cholesterol in hypercholesterolemia and its relationship to plasma triglycerides	1973	Sodhi, H. S.	Clinica Chimica Acta	LCA	Feces Concentration	Case Control	Adults	Hypercholesterolemia Hypertriglyceridemia	Metabolic Disorders	16
Excretion of tryptophan metabolites in rheumatoid arthritis	1966	Spiera, H.	N. Y.Arthr. Rheum.	Tryptamine	Urine Concentration	Case Control	Adults	Rheumatoid Arthritis	Other	54
Bile acids associate with specific gut microbiota, low-level alcohol consumption and liver fibrosis in patients with non-alcoholic fatty liver disease.	2020	Leon A Adams	Liver international : official journal of the International Association for the Study of the Liver	GDCA	Blood Concentration	Case Control	Adults	Non-Alcoholic Fatty Liver Disease	Hepatobiliary Disorders	122
Bile acids associate with specific gut microbiota, low-level alcohol consumption and liver fibrosis in patients with non-alcoholic fatty liver disease.	2020	Leon A Adams	Liver international : official journal of the International Association for the Study of the Liver	LCA	Blood Concentration	Case Control	Adults	Non-Alcoholic Fatty Liver Disease	Hepatobiliary Disorders	122
Bile acids associate with specific gut microbiota, low-level alcohol consumption and liver fibrosis in patients with non-alcoholic fatty liver disease.	2020	Leon A Adams	Liver international : official journal of the International Association for the Study of the Liver	DCA	Blood Concentration	Case Control	Adults	Non-Alcoholic Fatty Liver Disease	Hepatobiliary Disorders	122
Altered bile acid profile associates with cognitive impairment in Alzheimer's disease-An emerging role for gut microbiome.	2019	Siamak MahmoudianDehkordi	Alzheimer's & dementia : the journal of the Alzheimer's Association	DCA	Blood Concentration	Case Control	Adults	Cognitive Impairment Alzheimer's Disease	Neurological Disorders	1464
Altered bile acid profile associates with cognitive impairment in Alzheimer's disease-An emerging role for gut microbiome.	2019	Siamak MahmoudianDehkordi	Alzheimer's & dementia : the journal of the Alzheimer's Association	GLCA	Blood Concentration	Case Control	Adults	Cognitive Impairment Alzheimer's Disease	Neurological Disorders	1464
Altered bile acid profile associates with cognitive impairment in Alzheimer's disease-An emerging role for gut microbiome.	2019	Siamak MahmoudianDehkordi	Alzheimer's & dementia : the journal of the Alzheimer's Association	GDCA	Blood Concentration	Case Control	Adults	Cognitive Impairment Alzheimer's Disease	Neurological Disorders	1464
Polyamine concentrations in red cells and urine of patients with chronic renal failure	1980	Swendseid, M. E.	Life Sciences	Putrescine	Urine Concentration	Case Control	Adults	Renal Failure	Renal Disorders	30
Abnormalities of bile acids in serum and bile from patients with myotonic muscular dystrophy	1982	Tanaka, K.	Clinical Science	DCA	Blood Concentration	Case Control	Adults	Myotonic Muscular Dystrophy	Neurological Disorders	29
Abnormalities of bile acids in serum and bile from patients with myotonic muscular dystrophy	1982	Tanaka, K.	Clinical Science	LCA	Blood Concentration	Case Control	Adults	Myotonic Muscular Dystrophy	Neurological Disorders	29
Rapid identification and quantitation of urinary metabolites of phenylalanine in phenylketonuria by gas chromatography	1971	Vavich, J. M.	Journal of Laboratory and Clinical Medicine	PAG	Urine Concentration	Case Control	Adults	Phenylketonuria	Metabolic Disorders	25
Urinary excretion of polyamines by patients with advanced malignancy	1975	Waalkes, T. P.	CANCER CHEMOTHER.REP.	Putrescine	Urine Concentration	Case Control	Adults	Solid Tumor Malignancy	Cancer (Excluding Colorectal and	557

									Hepatobiliary Cancer)	
Primary bile acid kinetics in patients with primary biliary cirrhosis and in normal subjects	1979	Williams, C. N.	Clinical and Investigative Medicine	DCA	Blood Concentration	Case Control	Adults	Primary Biliary Cirrhosis	Hepatobiliary Disorders	22
Urinary excretion of free and acetylated polyamines in hepatocellular carcinoma	1998	Antoniello, S.	International Journal of Biological Markers	Putrescine	Urine Concentration	Case Control	Adults	Hepatocellular Carcinoma<br>Liver Cirrhosis	Hepatobiliary Disorders	76
Urinary excretion of free and acetylated polyamines in hepatocellular carcinoma	1998	Antoniello, S.	International Journal of Biological Markers	Putrescine	Urine Concentration	Case Control	Adults	Hepatocellular Carcinoma<br>Liver Cirrhosis	Hepatobiliary Cancer	76
GC-MS measurement of spermidine and putrescine in serum of elderly subjects: intriguing association between spermidine and homoarginine.	2020	Erik Hanff	Amino acids	Putrescine	Blood Concentration	Case Control	Adults	Helicobacter Pylori Infection	Digestive Disorders	27
Serum and urine concentrations of diamines and polyamines in patients with uterine, ovarian, breast or rectal cancer	1999	Bandyopadhyay , M.	Medical Science Research	Putrescine	Urine Concentration	Case Control	Adults	Uterine Cancer<br>Ovarian Cancer<br>Breast Cancer<br>Rectum Cancer	Cancer (Excluding Colorectal and Hepatobiliary Cancer)	30
Serum and urine concentrations of diamines and polyamines in patients with uterine, ovarian, breast or rectal cancer	1999	Bandyopadhyay , M.	Medical Science Research	Putrescine	Urine Concentration	Case Control	Adults	Uterine Cancer<br>Ovarian Cancer<br>Breast Cancer<br>Rectum Cancer	Colorectal Cancer	30
Serum and urine concentrations of diamines and polyamines in patients with uterine, ovarian, breast or rectal cancer	1999	Bandyopadhyay , M.	Medical Science Research	Putrescine	Blood Concentration	Case Control	Adults	Uterine Cancer<br>Ovarian Cancer<br>Breast Cancer<br>Rectum Cancer	Cancer (Excluding Colorectal and Hepatobiliary Cancer)	30
Serum and urine concentrations of diamines and polyamines in patients with uterine, ovarian, breast or rectal cancer	1999	Bandyopadhyay , M.	Medical Science Research	Putrescine	Blood Concentration	Case Control	Adults	Uterine Cancer<br>Ovarian Cancer<br>Breast Cancer<br>Rectum Cancer	Colorectal Cancer	30
Diet-Related Metabolites Associated with Cognitive Decline Revealed by Untargeted Metabolomics in a Prospective Cohort.	2019	Dorrain Yanwen Low	Molecular nutrition & food research	GDCA	Blood Concentration	Case Control	Adults	Cognitive Decline	Neurological Disorders	418
A pilot study of fecal bile acid and microbiota profiles in inflammatory bowel disease and primary sclerosing cholangitis.	2019	Byron P Vaughn	Clinical and experimental gastroenterology	DCA	Feces Concentration	Case Control	Adults	Inflammatory Bowel Disease<br>Primary Sclerosing Cholangitis	Inflammatory Bowel Disease	23

A pilot study of fecal bile acid and microbiota profiles in inflammatory bowel disease and primary sclerosing cholangitis.	2019	Byron P Vaughn	Clinical and experimental gastroenterology	LCA	Feces Concentration	Case Control	Adults	Inflammatory Bowel Disease<br>Primary Sclerosing Cholangitis	Inflammatory Bowel Disease	23
Colonic mucosal proliferation is related to serum deoxycholic acid levels	1999	Ochsenkühn, T.	Cancer	DCA	Blood Concentration	Case Control	Adults	Colorectal Adenoma	Digestive Disorders	19
Colonic mucosal proliferation is related to serum deoxycholic acid levels	1999	Ochsenkühn, T.	Cancer	LCA	Blood Concentration	Case Control	Adults	Colorectal Adenoma	Digestive Disorders	19
Recurrent Clostridium difficile infection associates with distinct bile acid and microbiome profiles.	2016	J R Allegretti	Alimentary pharmacology & therapeutics	DCA	Blood Concentration	Case Control	Adults	Clostridium Difficile Infection	Digestive Disorders	60
Recurrent Clostridium difficile infection associates with distinct bile acid and microbiome profiles.	2016	J R Allegretti	Alimentary pharmacology & therapeutics	DCA	Feces Concentration	Case Control	Adults	Clostridium Difficile Infection	Digestive Disorders	60
Recurrent Clostridium difficile infection associates with distinct bile acid and microbiome profiles.	2016	J R Allegretti	Alimentary pharmacology & therapeutics	LCA	Blood Concentration	Case Control	Adults	Clostridium Difficile Infection	Digestive Disorders	60
Recurrent Clostridium difficile infection associates with distinct bile acid and microbiome profiles.	2016	J R Allegretti	Alimentary pharmacology & therapeutics	LCA	Feces Concentration	Case Control	Adults	Clostridium Difficile Infection	Digestive Disorders	60
Indole production in hartnup disease	1963	Asatoor, A. M.	Lancet	Tryptamine	Feces Concentration	Case Control	Adults	Hartnup Disease	Metabolic Disorders	27
A measurement of individual bile acids in serum by high performance liquid chromatography for clinical diagnostic information of hepatobiliary diseases	1980	Baba, S.	Kobe Journal of Medical Sciences	DCA	Blood Concentration	Case Control	Adults	Hepatitis<br>Cholestasis	Hepatobiliary Disorders	84
A measurement of individual bile acids in serum by high performance liquid chromatography for clinical diagnostic information of hepatobiliary diseases	1980	Baba, S.	Kobe Journal of Medical Sciences	LCA	Blood Concentration	Case Control	Adults	Hepatitis<br>Cholestasis	Hepatobiliary Disorders	84
A measurement of individual bile acids in serum by high performance liquid chromatography for clinical diagnostic information of hepatobiliary diseases	1980	Baba, S.	Kobe Journal of Medical Sciences	GDCA	Blood Concentration	Case Control	Adults	Hepatitis<br>Cholestasis	Hepatobiliary Disorders	84
A measurement of individual bile acids in serum by high performance liquid chromatography for clinical diagnostic information of hepatobiliary diseases	1980	Baba, S.	Kobe Journal of Medical Sciences	GLCA	Blood Concentration	Case Control	Adults	Hepatitis<br>Cholestasis	Hepatobiliary Disorders	84
Age related abnormalities of circulating polyamines and diamine oxidase activity in cystic fibrosis heterozygotes and homozygotes	1980	Baylin, S. B.	Pediatric Research	Putrescine	Blood Concentration	Case Control	Adults	Cystic Fibrosis	Other	190

Bile acid feeding and hepatic sterol metabolism: Effect of deoxycholic acid	1980	Carulli, N.	Gastroenterology	DCA	Oral Intake	Clinical Trial	Adults	Cholesterol Gallstones	Hepatobiliary Disorders	18
Plasma polyamine levels in patients with liver insufficiency	1981	Desser, H.	Journal of Clinical Chemistry and Clinical Biochemistry	Putrescine	Blood Concentration	Case Control	Adults	Liver Disease	Hepatobiliary Disorders	64
Hepatotoxic effect of bile acids in inflammatory bowel disease	1980	Dew, M. J.	Gastroenterology	DCA	Blood Concentration	Case Control	Adults	Inflammatory Bowel Disease	Inflammatory Bowel Disease	13
Hepatotoxic effect of bile acids in inflammatory bowel disease	1980	Dew, M. J.	Gastroenterology	LCA	Blood Concentration	Case Control	Adults	Inflammatory Bowel Disease	Inflammatory Bowel Disease	13
Use of microextraction by packed sorbents and gas chromatography-mass spectrometry for the determination of polyamines and related compounds in urine.	2016	Ana María Casas Ferreira	Journal of chromatography. A	Putrescine	Urine Concentration	Case Control	Adults	Lung Cancer<br>Lung Cancer<br>Hepatocellular Carcinoma	Hepatobiliary Cancer	11
Use of microextraction by packed sorbents and gas chromatography-mass spectrometry for the determination of polyamines and related compounds in urine.	2016	Ana María Casas Ferreira	Journal of chromatography. A	Putrescine	Urine Concentration	Case Control	Adults	Lung Cancer<br>Lung Cancer<br>Hepatocellular Carcinoma	Cancer (Excluding Colorectal and Hepatobiliary Cancer)	11
The diagnostic value of fasting individual serum bile acids in anicteric alcoholic liver disease: Relation to liver morphology	1985	Einarsson, K.	Hepatology	DCA	Blood Concentration	Case Control	Adults	Fatty Liver Disease<br>Alcoholic Liver Cirrhosis	Hepatobiliary Disorders	46
Conjugated bile acids in gallbladder bile and serum as potential biomarkers for cholesterol polyps and adenomatous polyps.	2016	Mei-Fen Zhao	The International journal of biological markers	GDCA	Blood Concentration	Case Control	Adults	Cholesterol Polyps<br>Adenomatous Polyps<br>Gallstones	Hepatobiliary Disorders	47
Prediagnostic concentrations of circulating bile acids and hepatocellular carcinoma risk: REVEAL-HBV and HCV studies.	2020	Jessica L Petrick	International journal of cancer	GDCA	Blood Concentration	Case Control	Adults	Hepatocellular Carcinoma<br>Hepatitis B/C	Hepatobiliary Disorders	538
Prediagnostic concentrations of circulating bile acids and hepatocellular carcinoma risk: REVEAL-HBV and HCV studies.	2020	Jessica L Petrick	International journal of cancer	GDCA	Blood Concentration	Case Control	Adults	Hepatocellular Carcinoma<br>Hepatitis B/C	Hepatobiliary Cancer	538
Prediagnostic concentrations of circulating bile acids and hepatocellular carcinoma risk: REVEAL-HBV and HCV studies.	2020	Jessica L Petrick	International journal of cancer	LCA	Blood Concentration	Case Control	Adults	Hepatocellular Carcinoma<br>Hepatitis B/C	Hepatobiliary Disorders	538
Prediagnostic concentrations of circulating bile acids and hepatocellular carcinoma risk: REVEAL-HBV and HCV studies.	2020	Jessica L Petrick	International journal of cancer	LCA	Blood Concentration	Case Control	Adults	Hepatocellular Carcinoma<br>Hepatitis B/C	Hepatobiliary Cancer	538
Prediagnostic concentrations of circulating bile acids and hepatocellular carcinoma risk: REVEAL-HBV and HCV studies.	2020	Jessica L Petrick	International journal of cancer	DCA	Blood Concentration	Case Control	Adults	Hepatocellular Carcinoma<br>Hepatitis B/C	Hepatobiliary Disorders	538
Prediagnostic concentrations of circulating bile acids and hepatocellular carcinoma risk: REVEAL-HBV and HCV studies.	2020	Jessica L Petrick	International journal of cancer	DCA	Blood Concentration	Case Control	Adults	Hepatocellular Carcinoma<br>Hepatitis B/C	Hepatobiliary Cancer	538

Prediagnostic concentrations of circulating bile acids and hepatocellular carcinoma risk: REVEAL-HBV and HCV studies.	2020	Jessica L Petrick	International journal of cancer	GLCA	Blood Concentration	Case Control	Adults	Hepatocellular Carcinoma<br>Hepatitis B/C	Hepatobiliary Disorders	538
Prediagnostic concentrations of circulating bile acids and hepatocellular carcinoma risk: REVEAL-HBV and HCV studies.	2020	Jessica L Petrick	International journal of cancer	GLCA	Blood Concentration	Case Control	Adults	Hepatocellular Carcinoma<br>Hepatitis B/C	Hepatobiliary Cancer	538
Conjugated amino acids in plasma of patients with uremia	1961	Frimpter, G. W.	Journal of Clinical Investigation	PAG	Blood Concentration	Case Control	Adults	Chronic Renal Insufficiency	Renal Disorders	15
Non-targeted metabolomics combined with genetic analyses identifies bile acid synthesis and phospholipid metabolism as being associated with incident type 2 diabetes.	2016	Tove Fall	Diabetologia	DCA	Blood Concentration	Case Control	Adults	Impaired Fasting Glucose<br>Diabetes	Diabetes/Impaired Glucose Metabolism	4593
Non-targeted metabolomics combined with genetic analyses identifies bile acid synthesis and phospholipid metabolism as being associated with incident type 2 diabetes.	2016	Tove Fall	Diabetologia	DCA	Blood Concentration	Cohort Study	Adults	Incidence of Diabetes	Diabetes/Impaired Glucose Metabolism	4593
Urinary metabolic signatures of human adiposity.	2015	Paul Elliott	Science translational medicine	PAG	Urine Concentration	Case Control	Adults	Adiposity	Metabolic Disorders	1880
Integrated metagenome and metabolome analyses of blood pressure studies in early postmenopausal Chinese women.	2021	Hui-Min Liu	Journal of hypertension	PAG	Blood Concentration	Cohort Study	Adults	Blood Pressure in Early Postmenopausal Women	Cardiovascular Disorders	402
Altered metabolism of bile acids correlates with clinical parameters and the gut microbiota in patients with diarrhea-predominant irritable bowel syndrome.	2020	Wei Wei	World journal of gastroenterology	DCA	Feces Concentration	Case Control	Adults	Irritable Bowel Syndrome	Digestive Disorders	83
Altered metabolism of bile acids correlates with clinical parameters and the gut microbiota in patients with diarrhea-predominant irritable bowel syndrome.	2020	Wei Wei	World journal of gastroenterology	GDCA	Feces Concentration	Case Control	Adults	Irritable Bowel Syndrome	Digestive Disorders	83
Altered metabolism of bile acids correlates with clinical parameters and the gut microbiota in patients with diarrhea-predominant irritable bowel syndrome.	2020	Wei Wei	World journal of gastroenterology	LCA	Feces Concentration	Case Control	Adults	Irritable Bowel Syndrome	Digestive Disorders	83
Altered metabolism of bile acids correlates with clinical parameters and the gut microbiota in patients with diarrhea-predominant irritable bowel syndrome.	2020	Wei Wei	World journal of gastroenterology	GLCA	Feces Concentration	Case Control	Adults	Irritable Bowel Syndrome	Digestive Disorders	83
Clinical significance of serum spermine in breast cancer.	1988	Inamdar, N. A.	Tumori	Putrescine	Blood Concentration	Case Control	Adults	Breast Cancer<br>Advanced Breast Cancer<br>Benign Disease	Cancer (Excluding Colorectal and	150

									Hepatobiliary Cancer)	
Increased uptake of oxidized LDL by macrophages from type 2 diabetics is inhibited by polyamines.	2016	Francisco L Balderas	Biomedicine & pharmacotherapy = Biomedecine & pharmacotherapie	Putrescine	Blood Concentration	Case Control	Adults	Diabetics	Diabetes/Impaired Glucose Metabolism	12
Studies on the metabolism of aromatic amines in relation to altered thyroid function in man.	1962	Levine, R. J.	Journal of Clinical Endocrinology	Tryptamine	Urine Concentration	Case Control	Adults	Thyrotoxic Hypothyroid	Other	14
Gut microbiota and metabolite alterations associated with reduced bone mineral density or bone metabolic indexes in postmenopausal osteoporosis.	2020	Jianquan He	Aging	N-Acetylmannosamine	Feces Concentration	Case Control	Adults	Osteoporosis Osteopenia	Other	106
Metabolic products of the intestinal microbiome and extremes of atherosclerosis.	2018	Chrysi Bogiatzi	Atherosclerosis	PAG	Blood Concentration	Case Control	Adults	Carotid Atherosclerosis	Cardiovascular Disorders	316
Metabolic products of the intestinal microbiome and extremes of atherosclerosis.	2018	Chrysi Bogiatzi	Atherosclerosis	PAG	Oral Intake	Case Control	Adults	Carotid Atherosclerosis	Cardiovascular Disorders	316
Microbiota Composition and Metabolism Are Associated With Gut Function in Parkinson's Disease.	2020	Mihai S Cirstea	Movement disorders : official journal of the Movement Disorder Society	PAG	Feces Concentration	Case Control	Adults	Parkinson'S Disease	Neurological Disorders	300
Microbiota Composition and Metabolism Are Associated With Gut Function in Parkinson's Disease.	2020	Mihai S Cirstea	Movement disorders : official journal of the Movement Disorder Society	DCA	Feces Concentration	Case Control	Adults	Parkinson'S Disease	Neurological Disorders	300
Determination of polyamine metabolome in plasma and urine by ultrahigh performance liquid chromatography-tandem mass spectrometry method: application to identify potential markers for human hepatic cancer.	2013	Ran Liu	Analytica chimica acta	Putrescine	Urine Concentration	Case Control	Adults	Hepatic Cancer	Hepatobiliary Cancer	40
Determination of polyamine metabolome in plasma and urine by ultrahigh performance liquid chromatography-tandem mass spectrometry method: application to identify potential markers for human hepatic cancer.	2013	Ran Liu	Analytica chimica acta	Putrescine	Blood Concentration	Case Control	Adults	Hepatic Cancer	Hepatobiliary Cancer	40
Immunosuppression in cervical cancer with special reference to arginase activity.	2014	Astrid M Bedoya	Gynecologic oncology	Putrescine	Blood Concentration	Case Control	Adults	Cervical Cancer Low-Grade Squamous Intraepithelial Lesion	Other	207
Immunosuppression in cervical cancer with special reference to arginase activity.	2014	Astrid M Bedoya	Gynecologic oncology	Putrescine	Blood Concentration	Case Control	Adults	Cervical Cancer Low-Grade Squamous Intraepithelial Lesion	Cancer (Excluding Colorectal and Hepatobiliary Cancer)	207

Metabolomic evaluation of the response to endocrine therapy in patients with prostate cancer.	2014	Gang Huang	European journal of pharmacology	DCA	Blood Concentration	Case Control	Adults	Prostate Cancer	Cancer (Excluding Colorectal and Hepatobiliary Cancer)	72
NASH-related increases in plasma bile acid levels depend on insulin resistance.	2021	Guillaume Grzych	JHEP reports : innovation in hepatology	GDCA	Blood Concentration	Case Control	Adults	Type 2 Diabetes Non-Alcoholic Steatohepatitis Obesity	Metabolic Disorders	219
NASH-related increases in plasma bile acid levels depend on insulin resistance.	2021	Guillaume Grzych	JHEP reports : innovation in hepatology	GDCA	Blood Concentration	Case Control	Adults	Type 2 Diabetes Non-Alcoholic Steatohepatitis Obesity	Diabetes/Impaire d Glucose Metabolism	219
NASH-related increases in plasma bile acid levels depend on insulin resistance.	2021	Guillaume Grzych	JHEP reports : innovation in hepatology	GLCA	Blood Concentration	Case Control	Adults	Type 2 Diabetes Non-Alcoholic Steatohepatitis Obesity	Metabolic Disorders	219
NASH-related increases in plasma bile acid levels depend on insulin resistance.	2021	Guillaume Grzych	JHEP reports : innovation in hepatology	LCA	Blood Concentration	Case Control	Adults	Type 2 Diabetes Non-Alcoholic Steatohepatitis Obesity	Hepatobiliary Disorders	219
NASH-related increases in plasma bile acid levels depend on insulin resistance.	2021	Guillaume Grzych	JHEP reports : innovation in hepatology	LCA	Blood Concentration	Case Control	Adults	Type 2 Diabetes Non-Alcoholic Steatohepatitis Obesity	Metabolic Disorders	219
NASH-related increases in plasma bile acid levels depend on insulin resistance.	2021	Guillaume Grzych	JHEP reports : innovation in hepatology	LCA	Blood Concentration	Case Control	Adults	Type 2 Diabetes Non-Alcoholic Steatohepatitis Obesity	Diabetes/Impaire d Glucose Metabolism	219
NASH-related increases in plasma bile acid levels depend on insulin resistance.	2021	Guillaume Grzych	JHEP reports : innovation in hepatology	GDCA	Blood Concentration	Case Control	Adults	Type 2 Diabetes Non-Alcoholic Steatohepatitis Obesity	Hepatobiliary Disorders	219
NASH-related increases in plasma bile acid levels depend on insulin resistance.	2021	Guillaume Grzych	JHEP reports : innovation in hepatology	DCA	Blood Concentration	Case Control	Adults	Type 2 Diabetes Non-Alcoholic Steatohepatitis Obesity	Metabolic Disorders	219
NASH-related increases in plasma bile acid levels depend on insulin resistance.	2021	Guillaume Grzych	JHEP reports : innovation in hepatology	DCA	Blood Concentration	Case Control	Adults	Type 2 Diabetes Non-Alcoholic Steatohepatitis Obesity	Hepatobiliary Disorders	219

NASH-related increases in plasma bile acid levels depend on insulin resistance.	2021	Guillaume Grzych	JHEP reports : innovation in hepatology	DCA	Blood Concentration	Case Control	Adults	Type 2 Diabetes<br>Non-Alcoholic Steatohepatitis<br>Obesity	Diabetes/Impaired Glucose Metabolism	219
NASH-related increases in plasma bile acid levels depend on insulin resistance.	2021	Guillaume Grzych	JHEP reports : innovation in hepatology	GLCA	Blood Concentration	Case Control	Adults	Type 2 Diabetes<br>Non-Alcoholic Steatohepatitis<br>Obesity	Diabetes/Impaired Glucose Metabolism	219
NASH-related increases in plasma bile acid levels depend on insulin resistance.	2021	Guillaume Grzych	JHEP reports : innovation in hepatology	GLCA	Blood Concentration	Case Control	Adults	Type 2 Diabetes<br>Non-Alcoholic Steatohepatitis<br>Obesity	Hepatobiliary Disorders	219
Utilization of metabolomics to identify serum biomarkers for hepatocellular carcinoma in patients with liver cirrhosis.	2012	Habtom W Ressom	Analytica chimica acta	GDCA	Blood Concentration	Case Control	Adults	Hepatocellular Carcinoma<br>Liver Cirrhosis	Hepatobiliary Disorders	262
Utilization of metabolomics to identify serum biomarkers for hepatocellular carcinoma in patients with liver cirrhosis.	2012	Habtom W Ressom	Analytica chimica acta	GDCA	Blood Concentration	Case Control	Adults	Hepatocellular Carcinoma<br>Liver Cirrhosis	Hepatobiliary Cancer	262
Polyamines analysis by HPLC and their application as tumor markers.	2012	Shanji Fu	Frontiers in bioscience (Elite edition)	Putrescine	Blood Concentration	Case Control	Adults	Esophageal Cancer<br>Lung Cancer<br>Gastric Cancer<br>Breast Cancer<br>Ovarian Cancer<br>Liver Cancer<br>Lymphoma<br>Colorectal Cancer<br>Pancreas Cancer<br>Pharyneal Cancer<br>Glioma	Colorectal Cancer	11
Polyamines analysis by HPLC and their application as tumor markers.	2012	Shanji Fu	Frontiers in bioscience (Elite edition)	Putrescine	Blood Concentration	Case Control	Adults	Esophageal Cancer<br>Lung Cancer<br>Gastric Cancer<br>Breast Cancer<br>Ovarian Cancer<br>Liver Cancer<br>Lymphoma<br>Colorectal Cancer<br>Pancreas Cancer<br>Pharyneal Cancer<br>Glioma	Cancer (Excluding Colorectal and Hepatobiliary Cancer)	11
A dysregulated bile acid-gut microbiota axis contributes to obesity susceptibility.	2020	Meilin Wei	EBioMedicine	GDCA	Blood Concentration	Case Control	Adults	Metabolically Unhealthy	Metabolic Disorders	183

A dysregulated bile acid-gut microbiota axis contributes to obesity susceptibility.	2020	Meilin Wei	EBioMedicine	LCA	Blood Concentration	Case Control	Adults	Metabolically Unhealthy	Metabolic Disorders	183
A dysregulated bile acid-gut microbiota axis contributes to obesity susceptibility.	2020	Meilin Wei	EBioMedicine	DCA	Blood Concentration	Case Control	Adults	Metabolically Unhealthy	Metabolic Disorders	183
Metabolomics-based multidimensional network biomarkers for diabetic retinopathy identification in patients with type 2 diabetes mellitus.	2021	Jingjing Zuo	BMJ open diabetes research & care	PAG	Blood Concentration	Case Control	Adults	Diabetic Retinopathy Diabete without Diabetic Retinopathy	Other	138
Metabolomics-based multidimensional network biomarkers for diabetic retinopathy identification in patients with type 2 diabetes mellitus.	2021	Jingjing Zuo	BMJ open diabetes research & care	PAG	Blood Concentration	Case Control	Adults	Diabetic Retinopathy Diabete without Diabetic Retinopathy	Diabetes/Impaired Glucose Metabolism	138
Polyamines serum levels in episodic and chronic migraine.	2021	Luana Lionetto	Expert review of neurotherapeutics	Putrescine	Blood Concentration	Case Control	Adults	Migraine	Neurological Disorders	150
Bile Acids and Microbiome Among Individuals With Irritable Bowel Syndrome and Healthy Volunteers.	2021	Kendra J Kamp	Biological research for nursing	GDCA	Feces Concentration	Case Control	Adults	Irritable Bowel Syndrome	Digestive Disorders	73
Bile Acids and Microbiome Among Individuals With Irritable Bowel Syndrome and Healthy Volunteers.	2021	Kendra J Kamp	Biological research for nursing	DCA	Feces Concentration	Case Control	Adults	Irritable Bowel Syndrome	Digestive Disorders	73
Bile Acids and Microbiome Among Individuals With Irritable Bowel Syndrome and Healthy Volunteers.	2021	Kendra J Kamp	Biological research for nursing	LCA	Feces Concentration	Case Control	Adults	Irritable Bowel Syndrome	Digestive Disorders	73
Bile Acids and Microbiome Among Individuals With Irritable Bowel Syndrome and Healthy Volunteers.	2021	Kendra J Kamp	Biological research for nursing	GLCA	Feces Concentration	Case Control	Adults	Irritable Bowel Syndrome	Digestive Disorders	73
Colonic Transit and Bile Acid Synthesis or Excretion in Patients With Irritable Bowel Syndrome-Diarrhea Without Bile Acid Malabsorption.	2017	Cédric Peleman	Clinical gastroenterology and hepatology : the official clinical practice journal of the American Gastroenterological Association	DCA	Feces Concentration	Case Control	Adults	Irritable Bowel Syndrome	Digestive Disorders	146
Colonic Transit and Bile Acid Synthesis or Excretion in Patients With Irritable Bowel Syndrome-Diarrhea Without Bile Acid Malabsorption.	2017	Cédric Peleman	Clinical gastroenterology and hepatology : the official clinical practice journal of the American Gastroenterological Association	LCA	Feces Concentration	Case Control	Adults	Irritable Bowel Syndrome	Digestive Disorders	146
Lowered fasting chenodeoxycholic acid correlated with the decrease of fibroblast growth factor 19 in Chinese subjects with impaired fasting glucose.	2017	Jing Zhang	Scientific reports	DCA	Blood Concentration	Case Control	Adults	Impaired Glucose Tolerance	Diabetes/Impaired Glucose Metabolism	245
Machine-learning facilitates selection of a novel diagnostic panel of metabolites for the detection of heart failure.	2020	M Marcinkiewicz-Siemion	Scientific reports	DCA	Blood Concentration	Case Control	Adults	Chronic Heart Failure	Cardiovascular Disorders	106

Human insulin resistance is associated with increased plasma levels of 12α-hydroxylated bile acids.	2013	Rebecca A Haeusler	Diabetes	GDCA	Blood Concentration	Case Control	Adults	Insulin Sensitivity	Diabetes/Impaired Glucose Metabolism	230
Human insulin resistance is associated with increased plasma levels of 12α-hydroxylated bile acids.	2013	Rebecca A Haeusler	Diabetes	LCA	Blood Concentration	Case Control	Adults	Insulin Sensitivity	Diabetes/Impaired Glucose Metabolism	230
Human insulin resistance is associated with increased plasma levels of 12α-hydroxylated bile acids.	2013	Rebecca A Haeusler	Diabetes	DCA	Blood Concentration	Case Control	Adults	Insulin Sensitivity	Diabetes/Impaired Glucose Metabolism	230
Metabolomic profiling for second primary lung cancer: A pilot case-control study.	2021	Jacqueline V Aredo	Lung cancer (Amsterdam, Netherlands)	PAG	Blood Concentration	Case Control	Adults	Lung Cancer	Cancer (Excluding Colorectal and Hepatobiliary Cancer)	164
Metabolomic profiling for second primary lung cancer: A pilot case-control study.	2021	Jacqueline V Aredo	Lung cancer (Amsterdam, Netherlands)	GLCA	Blood Concentration	Case Control	Adults	Lung Cancer	Cancer (Excluding Colorectal and Hepatobiliary Cancer)	164
Altered profiles of fecal bile acids correlate with gut microbiota and inflammatory responses in patients with ulcerative colitis.	2021	Zhen-Huan Yang	World journal of gastroenterology	GDCA	Feces Concentration	Case Control	Adults	Ulcerative Colitis	Inflammatory Bowel Disease	55
Altered profiles of fecal bile acids correlate with gut microbiota and inflammatory responses in patients with ulcerative colitis.	2021	Zhen-Huan Yang	World journal of gastroenterology	LCA	Feces Concentration	Case Control	Adults	Ulcerative Colitis	Inflammatory Bowel Disease	55
Altered profiles of fecal bile acids correlate with gut microbiota and inflammatory responses in patients with ulcerative colitis.	2021	Zhen-Huan Yang	World journal of gastroenterology	DCA	Feces Concentration	Case Control	Adults	Ulcerative Colitis	Inflammatory Bowel Disease	55
Altered profiles of fecal bile acids correlate with gut microbiota and inflammatory responses in patients with ulcerative colitis.	2021	Zhen-Huan Yang	World journal of gastroenterology	GLCA	Feces Concentration	Case Control	Adults	Ulcerative Colitis	Inflammatory Bowel Disease	55
Global urinary metabolic profiling of the osteonecrosis of the femoral head based on UPLC-QTOF/MS.	2019	Gang Yang	Metabolomics : Official journal of the Metabolomic Society	PAG	Urine Concentration	Case Control	Adults	Osteonecrosis Of Femoral Head	Other	52
Global urinary metabolic profiling of the osteonecrosis of the femoral head based on UPLC-QTOF/MS.	2019	Gang Yang	Metabolomics : Official journal of the Metabolomic Society	DCA	Urine Concentration	Case Control	Adults	Osteonecrosis Of Femoral Head	Other	52
The membrane bile acid receptor TGR5 drives cell growth and migration via activation of the JAK2/STAT3 signaling pathway in non-small cell lung cancer.	2018	Xueqing Liu	Cancer letters	DCA	Blood Concentration	Case Control	Adults	Non-Small Cell Lung Cancer	Cancer (Excluding Colorectal and	86

									Hepatobiliary Cancer)	
The membrane bile acid receptor TGR5 drives cell growth and migration via activation of the JAK2/STAT3 signaling pathway in non-small cell lung cancer.	2018	Xueqing Liu	Cancer letters	LCA	Blood Concentration	Case Control	Adults	Non-Small Cell Lung Cancer	Cancer (Excluding Colorectal and Hepatobiliary Cancer)	86
Prediagnostic Plasma Bile Acid Levels and Colon Cancer Risk: A Prospective Study.	2020	Tilman Kühn	Journal of the National Cancer Institute	DCA	Blood Concentration	Case Control	Adults	Colon Cancer	Colorectal Cancer	1138
Prediagnostic Plasma Bile Acid Levels and Colon Cancer Risk: A Prospective Study.	2020	Tilman Kühn	Journal of the National Cancer Institute	GDCA	Blood Concentration	Case Control	Adults	Colon Cancer	Colorectal Cancer	1138
Alterations in the metabolism of phospholipids, bile acids and branched-chain amino acids predicts development of type 2 diabetes in black South African women: a prospective cohort study.	2019	Yingxu Zeng	Metabolism: clinical and experimental	DCA	Blood Concentration	Case Control	Adults	Type 2 Diabetes Impaired Glucose Tolerance	Diabetes/Impaired Glucose Metabolism	75
Alterations in the metabolism of phospholipids, bile acids and branched-chain amino acids predicts development of type 2 diabetes in black South African women: a prospective cohort study.	2019	Yingxu Zeng	Metabolism: clinical and experimental	GDCA	Blood Concentration	Case Control	Adults	Type 2 Diabetes Impaired Glucose Tolerance	Diabetes/Impaired Glucose Metabolism	75
LC-MS based serum metabolomics for identification of hepatocellular carcinoma biomarkers in Egyptian cohort.	2012	Jun Feng Xiao	Journal of proteome research	GDCA	Blood Concentration	Case Control	Adults	Hepatocellular Carcinoma Liver Cirrhosis	Hepatobiliary Disorders	89
LC-MS based serum metabolomics for identification of hepatocellular carcinoma biomarkers in Egyptian cohort.	2012	Jun Feng Xiao	Journal of proteome research	GDCA	Blood Concentration	Case Control	Adults	Hepatocellular Carcinoma Liver Cirrhosis	Hepatobiliary Cancer	89
Serum bile acids in cystic fibrosis patients-glycodeoxycholic acid as a potential marker of liver disease.	2021	Sławomira Drzymała-Czyż	Digestive and liver disease : official journal of the Italian Society of Gastroenterology and the Italian Association for the Study of the Liver	GDCA	Blood Concentration	Case Control	Adults	Cystic fibrosis (Exhibiting: Liver Cirrhosis, Other Liver Disease, or No Liver Disease)	Hepatobiliary Disorders	100
Serum bile acids in cystic fibrosis patients-glycodeoxycholic acid as a potential marker of liver disease.	2021	Sławomira Drzymała-Czyż	Digestive and liver disease : official journal of the Italian Society of Gastroenterology and the Italian Association for the Study of the Liver	LCA	Blood Concentration	Case Control	Adults	Cystic fibrosis (Exhibiting: Liver Cirrhosis, Other Liver Disease, or No Liver Disease)	Hepatobiliary Disorders	100
Serum bile acids in cystic fibrosis patients-glycodeoxycholic acid as a potential marker of liver disease.	2021	Sławomira Drzymała-Czyż	Digestive and liver disease : official journal of the Italian Society of Gastroenterology and	DCA	Blood Concentration	Case Control	Adults	Cystic fibrosis (Exhibiting: Liver Cirrhosis, Other Liver	Hepatobiliary Disorders	100

			the Italian Association for the Study of the Liver					Disease, or No Liver Disease)		
Oxidative stress markers, secondary bile acids and sulfated bile acids classify the clinical liver injury type: Promising diagnostic biomarkers for cholestasis.	2016	Noriko Masubuchi	Chemico-biological interactions	LCA	Blood Concentration	Case Control	Adults	Hepatocellular Injury Cholestasis	Hepatobiliary Disorders	311
Oxidative stress markers, secondary bile acids and sulfated bile acids classify the clinical liver injury type: Promising diagnostic biomarkers for cholestasis.	2016	Noriko Masubuchi	Chemico-biological interactions	DCA	Blood Concentration	Case Control	Adults	Hepatocellular Injury Cholestasis	Hepatobiliary Disorders	311
Microbiome and Metabolome Profiles Associated With Different Types of Short Bowel Syndrome: Implications for Treatment.	2020	Eva Budinska	JPEN. Journal of parenteral and enteral nutrition	DCA	Feces Concentration	Case Control	Adults	Short Bowel Syndrome	Other	69
Microbiome and Metabolome Profiles Associated With Different Types of Short Bowel Syndrome: Implications for Treatment.	2020	Eva Budinska	JPEN. Journal of parenteral and enteral nutrition	LCA	Feces Concentration	Case Control	Adults	Short Bowel Syndrome	Other	69
Plasma bile acid changes in type 2 diabetes correlated with insulin secretion in two-step hyperglycemic clamp.	2018	Shujie Wang	Journal of diabetes	LCA	Blood Concentration	Case Control	Adults	Type 2 Diabetes Mellitus	Diabetes/Impaire d Glucose Metabolism	44
Urinary profiling of tryptophan and its related metabolites in patients with metabolic syndrome by liquid chromatography-electrospray ionization/mass spectrometry.	2017	Ji Sun Oh	Analytical and bioanalytical chemistry	Tryptamine	Urine Concentration	Case Control	Adults	Metabolic Syndrome	Metabolic Disorders	345
Targeted metabolomics study of serum bile acid profile in patients with end-stage renal disease undergoing hemodialysis.	2019	Rong Li	PeerJ	DCA	Blood Concentration	Case Control	Adults	End-Stage Renal Disease	Renal Disorders	107
Assessment of serum bile acid profiles as biomarkers of liver injury and liver disease in humans.	2018	Lina Luo	PloS one	DCA	Blood Concentration	Case Control	Adults	Hepatic Injury Liver Cirrhosis	Hepatobiliary Disorders	645
Plasma bile acids are associated with energy expenditure and thyroid function in humans.	2012	Johann Ockenga	The Journal of clinical endocrinology and metabolism	DCA	Blood Concentration	Case Control	Adults	Cirrhosis	Hepatobiliary Disorders	18
Plasma bile acids are associated with energy expenditure and thyroid function in humans.	2012	Johann Ockenga	The Journal of clinical endocrinology and metabolism	GDCA	Blood Concentration	Case Control	Adults	Cirrhosis	Hepatobiliary Disorders	18
Altered Polyamine Profiles in Colorectal Cancer.	2018	Markus K Venäläinen	Anticancer research	Putrescine	Urine Concentration	Case Control	Adults	Colorectal Cancer Colorectal Adenomas Inflammatory Bowel Disease	Digestive Disorders	116
Altered Polyamine Profiles in Colorectal Cancer.	2018	Markus K Venäläinen	Anticancer research	Putrescine	Urine Concentration	Case Control	Adults	Colorectal Cancer Colorectal Adenomas Inflammatory Bowel Disease	Inflammatory Bowel Disease	116

Altered Polyamine Profiles in Colorectal Cancer.	2018	Markus K Venäläinen	Anticancer research	Putrescine	Urine Concentration	Case Control	Adults	Colorectal Cancer Colorectal Adenomas Inflammatory Bowel Disease	Colorectal Cancer	116
Detection of Gut Dysbiosis due to Reduced Clostridium Subcluster XIVa Using the Fecal or Serum Bile Acid Profile.	2018	Masashi Murakami	Inflammatory bowel diseases	DCA	Blood Concentration	Case Control	Adults	Crohn'S Disease Ulcerative Colitis	Inflammatory Bowel Disease	56
Bile Acids Elevated in Chronic Periaortitis Could Activate Farnesoid-X-Receptor to Suppress IL-6 Production by Macrophages.	2021	Shan Cao	Frontiers in immunology	DCA	Blood Concentration	Case Control	Adults	Chronic Periaortitis Takayasu’s Arteritis	Cardiovascular Disorders	38
Bile Acids Elevated in Chronic Periaortitis Could Activate Farnesoid-X-Receptor to Suppress IL-6 Production by Macrophages.	2021	Shan Cao	Frontiers in immunology	GDCA	Blood Concentration	Case Control	Adults	Chronic Periaortitis Takayasu’s Arteritis	Cardiovascular Disorders	38
Bile Acids Elevated in Chronic Periaortitis Could Activate Farnesoid-X-Receptor to Suppress IL-6 Production by Macrophages.	2021	Shan Cao	Frontiers in immunology	LCA	Blood Concentration	Case Control	Adults	Chronic Periaortitis Takayasu’s Arteritis	Cardiovascular Disorders	38
Bile Acids Elevated in Chronic Periaortitis Could Activate Farnesoid-X-Receptor to Suppress IL-6 Production by Macrophages.	2021	Shan Cao	Frontiers in immunology	GLCA	Blood Concentration	Case Control	Adults	Chronic Periaortitis Takayasu’s Arteritis	Cardiovascular Disorders	38
Modulation of the fecal bile acid profile by gut microbiota in cirrhosis.	2013	Genta Kakiyama	Journal of hepatology	GDCA	Blood Concentration	Case Control	Adults	Liver Cirrhosis	Hepatobiliary Disorders	61
Modulation of the fecal bile acid profile by gut microbiota in cirrhosis.	2013	Genta Kakiyama	Journal of hepatology	DCA	Blood Concentration	Case Control	Adults	Liver Cirrhosis	Hepatobiliary Disorders	61
Modulation of the fecal bile acid profile by gut microbiota in cirrhosis.	2013	Genta Kakiyama	Journal of hepatology	DCA	Feces Concentration	Case Control	Adults	Liver Cirrhosis	Hepatobiliary Disorders	61
Modulation of the fecal bile acid profile by gut microbiota in cirrhosis.	2013	Genta Kakiyama	Journal of hepatology	LCA	Blood Concentration	Case Control	Adults	Liver Cirrhosis	Hepatobiliary Disorders	61
Modulation of the fecal bile acid profile by gut microbiota in cirrhosis.	2013	Genta Kakiyama	Journal of hepatology	LCA	Feces Concentration	Case Control	Adults	Liver Cirrhosis	Hepatobiliary Disorders	61
Modulation of the fecal bile acid profile by gut microbiota in cirrhosis.	2013	Genta Kakiyama	Journal of hepatology	GLCA	Blood Concentration	Case Control	Adults	Liver Cirrhosis	Hepatobiliary Disorders	61
Gut Microbiota-Derived Inflammation-Related Serum Metabolites as Potential Biomarkers for Major Depressive Disorder.	2021	Shunjie Bai	Journal of inflammation research	DCA	Blood Concentration	Case Control	Adults	Major Depressive Disorder	Mental Disorders	120
Determination of polyamines in human plasma by high-performance liquid chromatography coupled with Q-TOF mass spectrometry.	2012	Ran Liu	Journal of mass spectrometry : JMS	Putrescine	Blood Concentration	Case Control	Adults	Cancer	Cancer (Excluding Colorectal and Hepatobiliary Cancer)	28
Abnormalities in gut microbiota and serum metabolites in hemodialysis patients with mild	2020	Bin Zhu	Psychopharmacology	Putrescine	Blood Concentration	Case Control	Adults	Mild Cognitive Decline	Neurological Disorders	106

cognitive decline: a single-center observational study.										
Metagenomic and metabolomic analyses reveal distinct stage-specific phenotypes of the gut microbiota in colorectal cancer.	2019	Shinichi Yachida	Nature medicine	DCA	Feces Concentration	Case Control	Adults	Multiple Polypoid Adenomas Colorectal Cancer	Digestive Disorders	406
Metagenomic and metabolomic analyses reveal distinct stage-specific phenotypes of the gut microbiota in colorectal cancer.	2019	Shinichi Yachida	Nature medicine	DCA	Feces Concentration	Case Control	Adults	Multiple Polypoid Adenomas Colorectal Cancer	Colorectal Cancer	406
Metagenomic and metabolomic analyses reveal distinct stage-specific phenotypes of the gut microbiota in colorectal cancer.	2019	Shinichi Yachida	Nature medicine	Tryptamine	Feces Concentration	Case Control	Adults	Multiple Polypoid Adenomas Colorectal Cancer	Digestive Disorders	406
Metagenomic and metabolomic analyses reveal distinct stage-specific phenotypes of the gut microbiota in colorectal cancer.	2019	Shinichi Yachida	Nature medicine	Tryptamine	Feces Concentration	Case Control	Adults	Multiple Polypoid Adenomas Colorectal Cancer	Colorectal Cancer	406
Metagenomic and metabolomic analyses reveal distinct stage-specific phenotypes of the gut microbiota in colorectal cancer.	2019	Shinichi Yachida	Nature medicine	Putrescine	Feces Concentration	Case Control	Adults	Multiple Polypoid Adenomas Colorectal Cancer	Digestive Disorders	406
Metagenomic and metabolomic analyses reveal distinct stage-specific phenotypes of the gut microbiota in colorectal cancer.	2019	Shinichi Yachida	Nature medicine	Putrescine	Feces Concentration	Case Control	Adults	Multiple Polypoid Adenomas Colorectal Cancer	Colorectal Cancer	406
Comprehensive bile acid profiling in hereditary intrahepatic cholestasis: Genetic and clinical correlations.	2018	Teng Liu	Liver international : official journal of the International Association for the Study of the Liver	DCA	Blood Concentration	Case Control	Adults	Cholestasis	Hepatobiliary Disorders	64
Comprehensive bile acid profiling in hereditary intrahepatic cholestasis: Genetic and clinical correlations.	2018	Teng Liu	Liver international : official journal of the International Association for the Study of the Liver	LCA	Blood Concentration	Case Control	Adults	Cholestasis	Hepatobiliary Disorders	64
The Associations between Circulating Bile Acids and the Muscle Volume in Patients with Non-alcoholic Fatty Liver Disease (NAFLD).	2017	Yoshinao Kobayashi	Internal medicine (Tokyo, Japan)	DCA	Blood Concentration	Cross-Sectional	Adults	Skeletal Muscle Volume in Non-alcoholic Fatty Liver Disease	Hepatobiliary Disorders	55
A blood-based, 7-metabolite signature for the early diagnosis of Alzheimer's disease.	2015	Javier Olazarán	Journal of Alzheimer's disease : JAD	LCA	Blood Concentration	Case Control	Adults	Mnestic Mild Cognitive Impairment Alzheimer’s Disease	Neurological Disorders	251
A blood-based, 7-metabolite signature for the early diagnosis of Alzheimer's disease.	2015	Javier Olazarán	Journal of Alzheimer's disease : JAD	DCA	Blood Concentration	Case Control	Adults	Mnestic Mild Cognitive Impairment Alzheimer’s Disease	Neurological Disorders	251
Urinary Polyamines: A Pilot Study on Their Roles as Prostate Cancer Detection Biomarkers.	2016	Tik-Hung Tsoi	PloS one	Putrescine	Urine Concentration	Case Control	Adults	Prostate Cancer Benign Prostatic Hyperplasia	Other	165

Urinary Polyamines: A Pilot Study on Their Roles as Prostate Cancer Detection Biomarkers.	2016	Tik-Hung Tsoi	PloS one	Putrescine	Urine Concentration	Case Control	Adults	Prostate Cancer Benign Prostatic Hyperplasia	Cancer (Excluding Colorectal and Hepatobiliary Cancer)	165
Circulating microbiota-derived metabolites: a "liquid biopsy?	2020	Gemma Aragonès	International journal of obesity (2005)	GDCA	Blood Concentration	Case Control	Adults	Morbid Obesity	Metabolic Disorders	111
Circulating microbiota-derived metabolites: a "liquid biopsy?	2020	Gemma Aragonès	International journal of obesity (2005)	LCA	Blood Concentration	Case Control	Adults	Morbid Obesity	Metabolic Disorders	111
Circulating microbiota-derived metabolites: a "liquid biopsy?	2020	Gemma Aragonès	International journal of obesity (2005)	DCA	Blood Concentration	Case Control	Adults	Morbid Obesity	Metabolic Disorders	111
Circulating microbiota-derived metabolites: a "liquid biopsy?	2020	Gemma Aragonès	International journal of obesity (2005)	GLCA	Blood Concentration	Case Control	Adults	Morbid Obesity	Metabolic Disorders	111
Serum metabolic signatures of subclinical atherosclerosis in patients with type 2 diabetes mellitus: a preliminary study.	2021	Jiaorong Su	Acta diabetologica	DCA	Blood Concentration	Case Control	Adults	Subclinical Atherosclerosis Type 2 Diabetes Mellitus	Diabetes/Impaired Glucose Metabolism	462
Serum metabolic signatures of subclinical atherosclerosis in patients with type 2 diabetes mellitus: a preliminary study.	2021	Jiaorong Su	Acta diabetologica	DCA	Blood Concentration	Case Control	Adults	Subclinical Atherosclerosis Type 2 Diabetes Mellitus	Cardiovascular Disorders	462
DCA-TGR5 signaling activation alleviates inflammatory response and improves cardiac function in myocardial infarction.	2021	Jiaxing Wang	Journal of molecular and cellular cardiology	DCA	Blood Concentration	Case Control	Adults	Myocardial Infarction	Cardiovascular Disorders	102
Derivatization enhanced separation and sensitivity of long chain-free fatty acids: Application to asthma using targeted and non-targeted liquid chromatography-mass spectrometry approach.	2017	Xiqing Bian	Analytica chimica acta	DCA	Blood Concentration	Case Control	Adults	Asthma	Respiratory Disorders	30
Bile and fat excretion are biomarkers of clinically significant diarrhoea and constipation in irritable bowel syndrome.	2019	Priya Vijayvargiya	Alimentary pharmacology & therapeutics	LCA	Feces Concentration	Case Control	Adults	Irritable Bowel Syndrome	Digestive Disorders	110
Bile and fat excretion are biomarkers of clinically significant diarrhoea and constipation in irritable bowel syndrome.	2019	Priya Vijayvargiya	Alimentary pharmacology & therapeutics	DCA	Feces Concentration	Case Control	Adults	Irritable Bowel Syndrome	Digestive Disorders	110
Bile Acids and Dysbiosis in Non-Alcoholic Fatty Liver Disease.	2016	Marialena Mouzaki	PloS one	LCA	Feces Concentration	Case Control	Adults	Non-Alcoholic Fatty Liver Disease	Hepatobiliary Disorders	53
Bile Acids and Dysbiosis in Non-Alcoholic Fatty Liver Disease.	2016	Marialena Mouzaki	PloS one	DCA	Feces Concentration	Case Control	Adults	Non-Alcoholic Fatty Liver Disease	Hepatobiliary Disorders	53
Bile Acids and Dysbiosis in Non-Alcoholic Fatty Liver Disease.	2016	Marialena Mouzaki	PloS one	GLCA	Feces Concentration	Case Control	Adults	Non-Alcoholic Fatty Liver Disease	Hepatobiliary Disorders	53

Tryptophan Metabolism, Inflammation, and Oxidative Stress in Patients with Neurovascular Disease.	2020	Martin Hajsl	Metabolites	Tryptamine	Blood Concentration	Case Control	Adults	Significant Carotid Artery Stenosis<br>Acute Ischemic Stroke	Cardiovascular Disorders	68
Polyamine Metabolites Profiling for Characterization of Lung and Liver Cancer Using an LC-Tandem MS Method with Multiple Statistical Data Mining Strategies: Discovering Potential Cancer Biomarkers in Human Plasma and Urine.	2016	Huarong Xu	Molecules (Basel, Switzerland)	Putrescine	Urine Concentration	Case Control	Adults	Lung Cancer<br>Liver Cancer	Cancer (Excluding Colorectal and Hepatobiliary Cancer)	150
Polyamine Metabolites Profiling for Characterization of Lung and Liver Cancer Using an LC-Tandem MS Method with Multiple Statistical Data Mining Strategies: Discovering Potential Cancer Biomarkers in Human Plasma and Urine.	2016	Huarong Xu	Molecules (Basel, Switzerland)	Putrescine	Blood Concentration	Case Control	Adults	Lung Cancer<br>Liver Cancer	Hepatobiliary Cancer	150
Polyamine Metabolites Profiling for Characterization of Lung and Liver Cancer Using an LC-Tandem MS Method with Multiple Statistical Data Mining Strategies: Discovering Potential Cancer Biomarkers in Human Plasma and Urine.	2016	Huarong Xu	Molecules (Basel, Switzerland)	Putrescine	Urine Concentration	Case Control	Adults	Lung Cancer<br>Liver Cancer	Hepatobiliary Cancer	150
Polyamine Metabolites Profiling for Characterization of Lung and Liver Cancer Using an LC-Tandem MS Method with Multiple Statistical Data Mining Strategies: Discovering Potential Cancer Biomarkers in Human Plasma and Urine.	2016	Huarong Xu	Molecules (Basel, Switzerland)	Putrescine	Blood Concentration	Case Control	Adults	Lung Cancer<br>Liver Cancer	Cancer (Excluding Colorectal and Hepatobiliary Cancer)	150
Dietary polyamine intake and risk of colorectal adenomatous polyps.	2012	Ashley J Vargas	The American journal of clinical nutrition	Putrescine	Oral Intake	Cohort Study	Adults	Colorectal Adenomatous Polyps	Digestive Disorders	2328
Serum polyamine metabolic profile in autoimmune thyroid disease patients.	2019	Jing Song	Clinical endocrinology	Putrescine	Blood Concentration	Case Control	Adults	Autoimmune Thyroid Disease	Other	136
Association of Serum Bile Acids Profile and Pathway Dysregulation With the Risk of Developing Diabetes Among Normoglycemic Chinese Adults: Findings From the 4C Study.	2021	Jieli Lu	Diabetes care	LCA	Blood Concentration	Case Control	Adults	Diabetes	Diabetes/Impaire d Glucose Metabolism	3414
Association of Serum Bile Acids Profile and Pathway Dysregulation With the Risk of Developing Diabetes Among Normoglycemic Chinese Adults: Findings From the 4C Study.	2021	Jieli Lu	Diabetes care	DCA	Blood Concentration	Case Control	Adults	Diabetes	Diabetes/Impaire d Glucose Metabolism	3414

Association of Serum Bile Acids Profile and Pathway Dysregulation With the Risk of Developing Diabetes Among Normoglycemic Chinese Adults: Findings From the 4C Study.	2021	Jieli Lu	Diabetes care	GLCA	Blood Concentration	Case Control	Adults	Diabetes	Diabetes/Impaire d Glucose Metabolism	3414
Association of Serum Bile Acids Profile and Pathway Dysregulation With the Risk of Developing Diabetes Among Normoglycemic Chinese Adults: Findings From the 4C Study.	2021	Jieli Lu	Diabetes care	GDCA	Blood Concentration	Case Control	Adults	Diabetes	Diabetes/Impaire d Glucose Metabolism	3414
Distinct urinary metabolic profile of human colorectal cancer.	2012	Yu Cheng	Journal of proteome research	Putrescine	Urine Concentration	Case Control	Adults	Colorectal Cancer	Colorectal Cancer	204
Characteristics of fecal metabolic profiles in patients with irritable bowel syndrome with predominant diarrhea investigated using.	2020	Jae Soung Lee	Neurogastroenterology and motility : the official journal of the European Gastrointestinal Motility Society	Putrescine	Feces Concentration	Case Control	Adults	Irritable Bowel Syndrome	Digestive Disorders	51
Metabolite Profiles in Sepsis: Developing Prognostic Tools Based on the Type of Infection.	2016	Sophie Neugebauer	Critical care medicine	Putrescine	Blood Concentration	Case Control	Adults	Systemic Inflammatory Response Syndrome<br>Community-Acquired Pneumonia<br>Urinary Tract Infection<br>Intra-Abdominal Infection<br>Bloodstream Infection	Other	406
Metabolite Profiles in Sepsis: Developing Prognostic Tools Based on the Type of Infection.	2016	Sophie Neugebauer	Critical care medicine	Putrescine	Blood Concentration	Case Control	Adults	Systemic Inflammatory Response Syndrome<br>Community-Acquired Pneumonia<br>Urinary Tract Infection<br>Intra-Abdominal Infection<br>Bloodstream Infection	Respiratory Disorders	406
Determination of putrescine, cadaverine, spermidine and spermine in different chemical matrices by high performance liquid chromatography-electrospray ionization-ion trap tandem mass spectrometry (HPLC-ESI-ITMS/MS).	2015	Alan Alexander González Ibarra	Journal of chromatography. B, Analytical technologies in the biomedical and life sciences	Putrescine	Urine Concentration	Case Control	Adults	Diabetes	Diabetes/Impaire d Glucose Metabolism	18
The Differences of Serum Metabolites Between Patients With Early-Stage Alzheimer's Disease and Mild Cognitive Impairment.	2019	Wei-Chieh Weng	Frontiers in neurology	Putrescine	Blood Concentration	Case Control	Adults	Alzheimer’s Disease<br>Mild Cognitive Impairment	Neurological Disorders	80

Plasma oxysterols: biomarkers for diagnosis and treatment in spastic paraplegia type 5.	2018	Cecilia Marelli	Brain : a journal of neurology	DCA	Blood Concentration	Case Control	Adults	Spastic Paraplegia	Neurological Disorders	30
Plasma oxysterols: biomarkers for diagnosis and treatment in spastic paraplegia type 5.	2018	Cecilia Marelli	Brain : a journal of neurology	LCA	Blood Concentration	Case Control	Adults	Spastic Paraplegia	Neurological Disorders	30
A monoclonal antibody-based enzyme-linked immunosorbent assay of glycolithocholic acid sulfate in human urine for liver function test.	2002	Norihiro Kobayashi	Steroids	GLCA	Urine Concentration	Case Control	Adults	Hepatitis B/C	Hepatobiliary Disorders	46
Capillary gas chromatographic determination of putrescine and cadaverine in serum of cancer patients using trifluoroacetylacetone as derivatizing reagent.	1999	M Y Khuhawar	Journal of chromatography. B, Biomedical sciences and applications	Putrescine	Blood Concentration	Case Control	Adults	Urinary Bladder Cancer<br>Head And Neck Cancer<br>Left Breast Cancer<br>Lung Cancer	Cancer (Excluding Colorectal and Hepatobiliary Cancer)	6
Analysis of bile acids in colon residual liquid or fecal material in patients with colorectal neoplasia and control subjects.	1997	T Kishida	Journal of gastroenterology	LCA	Feces Concentration	Case Control	Adults	Colorectal Adenoma<br>Colorectal Cancer	Digestive Disorders	48
Analysis of bile acids in colon residual liquid or fecal material in patients with colorectal neoplasia and control subjects.	1997	T Kishida	Journal of gastroenterology	LCA	Feces Concentration	Case Control	Adults	Colorectal Adenoma<br>Colorectal Cancer	Colorectal Cancer	48
Analysis of bile acids in colon residual liquid or fecal material in patients with colorectal neoplasia and control subjects.	1997	T Kishida	Journal of gastroenterology	DCA	Feces Concentration	Case Control	Adults	Colorectal Adenoma<br>Colorectal Cancer	Digestive Disorders	48
Analysis of bile acids in colon residual liquid or fecal material in patients with colorectal neoplasia and control subjects.	1997	T Kishida	Journal of gastroenterology	DCA	Feces Concentration	Case Control	Adults	Colorectal Adenoma<br>Colorectal Cancer	Colorectal Cancer	48
Portal vein bile acids in patients with severe inflammatory bowel disease.	1980	R T Holzbach	Gut	LCA	Blood Concentration	Case Control	Adults	Inflammatory Bowel Disease<br>Localised Colonic Lesions	Digestive Disorders	27
Portal vein bile acids in patients with severe inflammatory bowel disease.	1980	R T Holzbach	Gut	LCA	Blood Concentration	Case Control	Adults	Inflammatory Bowel Disease<br>Localised Colonic Lesions	Inflammatory Bowel Disease	27
Portal vein bile acids in patients with severe inflammatory bowel disease.	1980	R T Holzbach	Gut	DCA	Blood Concentration	Case Control	Adults	Inflammatory Bowel Disease<br>Localised Colonic Lesions	Inflammatory Bowel Disease	27
Portal vein bile acids in patients with severe inflammatory bowel disease.	1980	R T Holzbach	Gut	DCA	Blood Concentration	Case Control	Adults	Inflammatory Bowel Disease<br>Localised Colonic Lesions	Digestive Disorders	27

Faecal steroids and colorectal cancer: steroid profiles in subjects with adenomatous polyps of the large bowel.	1992	R W Owen	European journal of cancer prevention : the official journal of the European Cancer Prevention Organisation (ECP)	DCA	Feces Concentration	Case Control	Adults	Adenomatous Polyps	Digestive Disorders	92
Faecal steroids and colorectal cancer: steroid profiles in subjects with adenomatous polyps of the large bowel.	1992	R W Owen	European journal of cancer prevention : the official journal of the European Cancer Prevention Organisation (ECP)	LCA	Feces Concentration	Case Control	Adults	Adenomatous Polyps	Digestive Disorders	92
Gut-Microbiota-Metabolite Axis in Early Renal Function Decline.	2015	Clara Barrios	PloS one	PAG	Blood Concentration	Case Control	Adults	Early Renal Function Decline	Renal Disorders	4439
Serum, fecal and urinary bile acids in patients with mild and advanced liver cirrhosis.	1981	Y Amuro	Gastroenterologia Japonica	LCA	Urine Concentration	Case Control	Adults	Liver Cirrhosis	Hepatobiliary Disorders	28
Serum, fecal and urinary bile acids in patients with mild and advanced liver cirrhosis.	1981	Y Amuro	Gastroenterologia Japonica	LCA	Feces Concentration	Case Control	Adults	Liver Cirrhosis	Hepatobiliary Disorders	28
Serum, fecal and urinary bile acids in patients with mild and advanced liver cirrhosis.	1981	Y Amuro	Gastroenterologia Japonica	LCA	Blood Concentration	Case Control	Adults	Liver Cirrhosis	Hepatobiliary Disorders	28
Serum, fecal and urinary bile acids in patients with mild and advanced liver cirrhosis.	1981	Y Amuro	Gastroenterologia Japonica	DCA	Urine Concentration	Case Control	Adults	Liver Cirrhosis	Hepatobiliary Disorders	28
Serum, fecal and urinary bile acids in patients with mild and advanced liver cirrhosis.	1981	Y Amuro	Gastroenterologia Japonica	DCA	Blood Concentration	Case Control	Adults	Liver Cirrhosis	Hepatobiliary Disorders	28
Serum, fecal and urinary bile acids in patients with mild and advanced liver cirrhosis.	1981	Y Amuro	Gastroenterologia Japonica	DCA	Feces Concentration	Case Control	Adults	Liver Cirrhosis	Hepatobiliary Disorders	28
Bile Acid Dysmetabolism in the Gut-microbiota-liver Axis Under Hepatitis C Virus Infection.	2021	Takako Inoue	Liver international : official journal of the International Association for the Study of the Liver	LCA	Feces Concentration	Case Control	Adults	Hepatitis C Virus Infection	Hepatobiliary Disorders	123
Bile Acid Dysmetabolism in the Gut-microbiota-liver Axis Under Hepatitis C Virus Infection.	2021	Takako Inoue	Liver international : official journal of the International Association for the Study of the Liver	DCA	Feces Concentration	Case Control	Adults	Hepatitis C Virus Infection	Hepatobiliary Disorders	123
Faecal weight, constituents, colonic motility, and lactose tolerance in the irritable bowel syndrome.	1984	M A Eastwood	Digestion	DCA	Feces Concentration	Case Control	Adults	Irritable Bowel Syndrome	Digestive Disorders	32
Faecal weight, constituents, colonic motility, and lactose tolerance in the irritable bowel syndrome.	1984	M A Eastwood	Digestion	LCA	Feces Concentration	Case Control	Adults	Irritable Bowel Syndrome	Digestive Disorders	32
Proliferative activity of rectal mucosa and soluble fecal bile acids in patients with normal colons and in patients with colonic polyps or cancer.	1988	J Stadler	Cancer letters	DCA	Feces Concentration	Case Control	Adults	Colon Cancer Polyps	Digestive Disorders	34

Proliferative activity of rectal mucosa and soluble fecal bile acids in patients with normal colons and in patients with colonic polyps or cancer.	1988	J Stadler	Cancer letters	DCA	Feces Concentration	Case Control	Adults	Colon Cancer Polyps	Colorectal Cancer	34
Proliferative activity of rectal mucosa and soluble fecal bile acids in patients with normal colons and in patients with colonic polyps or cancer.	1988	J Stadler	Cancer letters	LCA	Feces Concentration	Case Control	Adults	Colon Cancer Polyps	Digestive Disorders	34
Proliferative activity of rectal mucosa and soluble fecal bile acids in patients with normal colons and in patients with colonic polyps or cancer.	1988	J Stadler	Cancer letters	LCA	Feces Concentration	Case Control	Adults	Colon Cancer Polyps	Colorectal Cancer	34
Quantitative determination of non-sulfated bile acids in the serum of patients with hepatobiliary diseases by mass fragmentography.	1983	H Takikawa	Gastroenterologia Japonica	DCA	Blood Concentration	Case Control	Adults	Obstructive Jaundice Acute Hepatitis Chronic Hepatitis Liver Cirrhosis	Hepatobiliary Disorders	85
Quantitative determination of non-sulfated bile acids in the serum of patients with hepatobiliary diseases by mass fragmentography.	1983	H Takikawa	Gastroenterologia Japonica	LCA	Blood Concentration	Case Control	Adults	Obstructive Jaundice Acute Hepatitis Chronic Hepatitis Liver Cirrhosis	Hepatobiliary Disorders	85
Levels of immunoreactive glycine-conjugated bile acids in health and hepatobiliary disease.	1976	L M Demers	American journal of clinical pathology	GDCA	Blood Concentration	Case Control	Adults	Hepatic Disease	Hepatobiliary Cancer	135
Bile acids of patients with renal failure receiving chronic hemodialysis.		N Yamaga	Steroids	DCA	Blood Concentration	Case Control	Adults	Renal Failure	Renal Disorders	14
Bile acids of patients with renal failure receiving chronic hemodialysis.		N Yamaga	Steroids	LCA	Blood Concentration	Case Control	Adults	Renal Failure	Renal Disorders	14
Faecal bile acid excretion in diverticular disease.	1980	M Flynn	The British journal of surgery	DCA	Feces Concentration	Case Control	Adults	Diverticular Disease	Digestive Disorders	38
Faecal bile acid excretion in diverticular disease.	1980	M Flynn	The British journal of surgery	LCA	Feces Concentration	Case Control	Adults	Diverticular Disease	Digestive Disorders	38
Steroids and cancer: faecal bile acid screening for early detection of cancer risk.	1986	R W Owen	Journal of steroid biochemistry	DCA	Feces Concentration	Case Control	Adults	Colorectal Cancer Breast Cancer	Colorectal Cancer	86
Steroids and cancer: faecal bile acid screening for early detection of cancer risk.	1986	R W Owen	Journal of steroid biochemistry	DCA	Feces Concentration	Case Control	Adults	Colorectal Cancer Breast Cancer	Cancer (Excluding Colorectal and Hepatobiliary Cancer)	86
Steroids and cancer: faecal bile acid screening for early detection of cancer risk.	1986	R W Owen	Journal of steroid biochemistry	LCA	Feces Concentration	Case Control	Adults	Colorectal Cancer Breast Cancer	Colorectal Cancer	86
Steroids and cancer: faecal bile acid screening for early detection of cancer risk.	1986	R W Owen	Journal of steroid biochemistry	LCA	Feces Concentration	Case Control	Adults	Colorectal Cancer Breast Cancer	Cancer (Excluding	86

									Colorectal and Hepatobiliary Cancer)	
Metabolic epidemiology of colon cancer. Fecal bile acids and neutral sterols in colon cancer patients and patients with adenomatous polyps.	1977	B S Reddy	Cancer	DCA	Feces Concentration	Case Control	Adults	Colon Cancer<br>Adenomatous Polyps<br>Other Digestive	Digestive Disorders	87
Metabolic epidemiology of colon cancer. Fecal bile acids and neutral sterols in colon cancer patients and patients with adenomatous polyps.	1977	B S Reddy	Cancer	DCA	Feces Concentration	Case Control	Adults	Colon Cancer<br>Adenomatous Polyps<br>Other Digestive	Colorectal Cancer	87
Metabolic epidemiology of colon cancer. Fecal bile acids and neutral sterols in colon cancer patients and patients with adenomatous polyps.	1977	B S Reddy	Cancer	LCA	Feces Concentration	Case Control	Adults	Colon Cancer<br>Adenomatous Polyps<br>Other Digestive	Digestive Disorders	87
Metabolic epidemiology of colon cancer. Fecal bile acids and neutral sterols in colon cancer patients and patients with adenomatous polyps.	1977	B S Reddy	Cancer	LCA	Feces Concentration	Case Control	Adults	Colon Cancer<br>Adenomatous Polyps<br>Other Digestive	Colorectal Cancer	87
Serum bile acid profiles in cerebrotendinous xanthomatosis.	1982	T Beppu	Clinica chimica acta; international journal of clinical chemistry	DCA	Blood Concentration	Case Control	Adults	Cerebrotendinous Xanthomatosis	Metabolic Disorders	30
Serum bile acid profiles in cerebrotendinous xanthomatosis.	1982	T Beppu	Clinica chimica acta; international journal of clinical chemistry	LCA	Blood Concentration	Case Control	Adults	Cerebrotendinous Xanthomatosis	Metabolic Disorders	30
Bile acid malabsorption in patients with an ileum reservoir with a long efferent leg to an anal anastomosis.	1985	B H Pedersen	Scandinavian journal of gastroenterology	DCA	Feces Concentration	Case Control	Adults	Ileum Reservoirs With Anal Anastomosis<br>A Long Efferent Leg	Digestive Disorders	23
Bile acid malabsorption in patients with an ileum reservoir with a long efferent leg to an anal anastomosis.	1985	B H Pedersen	Scandinavian journal of gastroenterology	LCA	Feces Concentration	Case Control	Adults	Ileum Reservoirs With Anal Anastomosis<br>A Long Efferent Leg	Digestive Disorders	23
Quantification of individual serum bile acids in patients with liver diseases using high-performance liquid chromatography.	1984	H Okuda	Hepato-gastroenterology	GDCA	Blood Concentration	Case Control	Adults	Acute Hepatitis<br>Liver Cirrhosis<br>Extrahepatic Cholestasis<br>Primary Biliary Cirrhosis	Hepatobiliary Disorders	47
Quantification of individual serum bile acids in patients with liver diseases using high-performance liquid chromatography.	1984	H Okuda	Hepato-gastroenterology	LCA	Blood Concentration	Case Control	Adults	Acute Hepatitis<br>Liver Cirrhosis<br>Extrahepatic Cholestasis	Hepatobiliary Disorders	47

								Primary Biliary Cirrhosis		
Quantification of individual serum bile acids in patients with liver diseases using high-performance liquid chromatography.	1984	H Okuda	Hepato-gastroenterology	DCA	Blood Concentration	Case Control	Adults	Acute Hepatitis<br>Liver Cirrhosis<br>Extrahepatic Cholestasis<br>Primary Biliary Cirrhosis	Hepatobiliary Disorders	47
Quantification of individual serum bile acids in patients with liver diseases using high-performance liquid chromatography.	1984	H Okuda	Hepato-gastroenterology	GLCA	Blood Concentration	Case Control	Adults	Acute Hepatitis<br>Liver Cirrhosis<br>Extrahepatic Cholestasis<br>Primary Biliary Cirrhosis	Hepatobiliary Disorders	47
Hepatic levels of bile acids in end-stage chronic cholestatic liver disease.	1996	S Fischer	Clinica chimica acta; international journal of clinical chemistry	DCA	Blood Concentration	Case Control	Adults	End-Stage Chronic Cholestatic Liver Disease<br>End-Stage Liver Cirrhosis Of Alcoholic Chronic Hepatitic Origin Who Underwent Liver Transplantation	Hepatobiliary Disorders	18
Hepatic levels of bile acids in end-stage chronic cholestatic liver disease.	1996	S Fischer	Clinica chimica acta; international journal of clinical chemistry	LCA	Blood Concentration	Case Control	Adults	End-Stage Chronic Cholestatic Liver Disease<br>End-Stage Liver Cirrhosis Of Alcoholic Chronic Hepatitic Origin Who Underwent Liver Transplantation	Hepatobiliary Disorders	18
Abnormal intestinal bile acid distribution in azotaemic man: a possible role in the pathogenesis of uraemic diarrhoea.	1976	S J Gordon	Gut	DCA	Feces Concentration	Case Control	Adults	Azotaemia	Renal Disorders	23
Abnormal intestinal bile acid distribution in azotaemic man: a possible role in the pathogenesis of uraemic diarrhoea.	1976	S J Gordon	Gut	LCA	Feces Concentration	Case Control	Adults	Azotaemia	Renal Disorders	23
Decreased concentrations of deoxycholic acid in serum of uraemic patients with diarrhoea.	1990	P Stenvinkel	Scandinavian journal of clinical and laboratory investigation	DCA	Blood Concentration	Case Control	Adults	Renal Failure With Diarrhea	Renal Disorders	42
Decreased concentrations of deoxycholic acid in serum of uraemic patients with diarrhoea.	1990	P Stenvinkel	Scandinavian journal of clinical and laboratory investigation	DCA	Blood Concentration	Case Control	Adults	Renal Failure With Diarrhea	Digestive Disorders	42

Cholic acid conjugation test and quantitative liver function in acute liver failure.	1983	A Bremmelgaard	Scandinavian journal of gastroenterology	GLCA	Blood Concentration	Case Control	Adults	Acute Liver Failure	Hepatobiliary Disorders	31
Biochemical epidemiology of gallbladder cancer.	1996	B L Strom	Hepatology (Baltimore, Md.)	GDCA	Blood Concentration	Case Control	Adults	Gallbladder Cancer Cholelithiasis Choledocholithiasis	Hepatobiliary Disorders	47
Biochemical epidemiology of gallbladder cancer.	1996	B L Strom	Hepatology (Baltimore, Md.)	GDCA	Blood Concentration	Case Control	Adults	Gallbladder Cancer Cholelithiasis Choledocholithiasis	Hepatobiliary Cancer	47
Biochemical epidemiology of gallbladder cancer.	1996	B L Strom	Hepatology (Baltimore, Md.)	LCA	Blood Concentration	Case Control	Adults	Gallbladder Cancer Cholelithiasis Choledocholithiasis	Hepatobiliary Disorders	47
Biochemical epidemiology of gallbladder cancer.	1996	B L Strom	Hepatology (Baltimore, Md.)	LCA	Blood Concentration	Case Control	Adults	Gallbladder Cancer Cholelithiasis Choledocholithiasis	Hepatobiliary Cancer	47
Biochemical epidemiology of gallbladder cancer.	1996	B L Strom	Hepatology (Baltimore, Md.)	DCA	Blood Concentration	Case Control	Adults	Gallbladder Cancer Cholelithiasis Choledocholithiasis	Hepatobiliary Disorders	47
Biochemical epidemiology of gallbladder cancer.	1996	B L Strom	Hepatology (Baltimore, Md.)	DCA	Blood Concentration	Case Control	Adults	Gallbladder Cancer Cholelithiasis Choledocholithiasis	Hepatobiliary Cancer	47
Biochemical epidemiology of gallbladder cancer.	1996	B L Strom	Hepatology (Baltimore, Md.)	GLCA	Blood Concentration	Case Control	Adults	Gallbladder Cancer Cholelithiasis Choledocholithiasis	Hepatobiliary Disorders	47
Biochemical epidemiology of gallbladder cancer.	1996	B L Strom	Hepatology (Baltimore, Md.)	GLCA	Blood Concentration	Case Control	Adults	Gallbladder Cancer Cholelithiasis Choledocholithiasis	Hepatobiliary Cancer	47
Daily determination of individual serum bile acids allows early detection of hepatic allograft dysfunction.	1994	S A Azer	Hepatology (Baltimore, Md.)	DCA	Blood Concentration	Case Control	Adults	Hepatic Allograft Dysfunction	Hepatobiliary Disorders	8
Urinary tryptamine excretion in chronic schizophrenics with low platelet MAO activity.	1980	J L Sullivan	Biological psychiatry	Tryptamine	Urine Concentration	Case Control	Adults	Chronic Schizophrenics	Mental Disorders	60
Increased serum deoxycholic acid levels in men with colorectal adenomas.	1993	E Bayerdörffer	Gastroenterology	LCA	Blood Concentration	Case Control	Adults	Colorectal Adenomas	Digestive Disorders	100
Increased serum deoxycholic acid levels in men with colorectal adenomas.	1993	E Bayerdörffer	Gastroenterology	DCA	Blood Concentration	Case Control	Adults	Colorectal Adenomas	Digestive Disorders	100
Ratio of primary and secondary bile acids in feces: possible marker for colorectal cancer?	1999	T Kamano	Diseases of the colon and rectum	DCA	Feces Concentration	Case Control	Adults	Colorectal Cancer Gastric Cancer Biliary Disorders	Hepatobiliary Disorders	142
Ratio of primary and secondary bile acids in feces: possible marker for colorectal cancer?	1999	T Kamano	Diseases of the colon and rectum	DCA	Feces Concentration	Case Control	Adults	Colorectal Cancer Gastric Cancer Biliary Disorders	Colorectal Cancer	142

Ratio of primary and secondary bile acids in feces: possible marker for colorectal cancer?	1999	T Kamano	Diseases of the colon and rectum	DCA	Feces Concentration	Case Control	Adults	Colorectal Cancer<br>Gastric Cancer<br>Biliary Disorders	Cancer (Excluding Colorectal and Hepatobiliary Cancer)	142
Serum bile acids, programmed cell death and cell proliferation in the mucosa of patients with colorectal adenomas.	2005	M Fracchia	Digestive and liver disease : official journal of the Italian Society of Gastroenterology and the Italian Association for the Study of the Liver	LCA	Blood Concentration	Case Control	Adults	Colorectal Adenomas	Digestive Disorders	10
Serum bile acids, programmed cell death and cell proliferation in the mucosa of patients with colorectal adenomas.	2005	M Fracchia	Digestive and liver disease : official journal of the Italian Society of Gastroenterology and the Italian Association for the Study of the Liver	DCA	Blood Concentration	Case Control	Adults	Colorectal Adenomas	Digestive Disorders	10
Metabolomic Analysis of Gastric Cancer Progression within the Correa's Cascade Using Ultraperformance Liquid Chromatography-Mass Spectrometry.	2016	Julia Kuligowski	Journal of proteome research	PAG	Blood Concentration	Case Control	Adults	H. Pylori Negative With Nonactive Gastritis<br>H. Pylori Positive With Chronic Active Gastritis<br>Precursor Lesions Of Gastric Cancer<br>Gastric Cancer	Digestive Disorders	143
Metabolomic Analysis of Gastric Cancer Progression within the Correa's Cascade Using Ultraperformance Liquid Chromatography-Mass Spectrometry.	2016	Julia Kuligowski	Journal of proteome research	PAG	Blood Concentration	Case Control	Adults	H. Pylori Negative With Nonactive Gastritis<br>H. Pylori Positive With Chronic Active Gastritis<br>Precursor Lesions Of Gastric Cancer<br>Gastric Cancer	Cancer (Excluding Colorectal and Hepatobiliary Cancer)	143
Microbiota-Derived Phenylacetylglutamine Associates with Overall Mortality and Cardiovascular Disease in Patients with CKD.	2016	Ruben Poesen	Journal of the American Society of Nephrology : JASN	PAG	Blood Concentration	Cohort Study	Adults	Overall Mortality And Cardiovascular Disease<br>Chronic Kidney Disease	Renal Disorders	488
Microbiota-Derived Phenylacetylglutamine Associates with Overall Mortality and Cardiovascular Disease in Patients with CKD.	2016	Ruben Poesen	Journal of the American Society of Nephrology : JASN	PAG	Blood Concentration	Cohort Study	Adults	Overall Mortality And Cardiovascular Disease<br>Chronic Kidney Disease	Cardiovascular Disorders	488

Metabolic alterations in plasma from patients with familial and idiopathic Parkinson's disease.	2020	Sokhna M S Yakhine-Diop	Aging	DCA	Blood Concentration	Case Control	Adults	Familial And Idiopathic Parkinson’S Disease	Neurological Disorders	32
Quantification of bile acids: a mass spectrometry platform for studying gut microbe connection to metabolic diseases.	2020	Ibrahim Choucair	Journal of lipid research	GDCA	Blood Concentration	Case Control	Adults	Type 2 Diabetes Mellitus	Diabetes/Impaired Glucose Metabolism	160
Quantification of bile acids: a mass spectrometry platform for studying gut microbe connection to metabolic diseases.	2020	Ibrahim Choucair	Journal of lipid research	LCA	Blood Concentration	Case Control	Adults	Type 2 Diabetes Mellitus	Diabetes/Impaired Glucose Metabolism	160
Quantification of bile acids: a mass spectrometry platform for studying gut microbe connection to metabolic diseases.	2020	Ibrahim Choucair	Journal of lipid research	DCA	Blood Concentration	Case Control	Adults	Type 2 Diabetes Mellitus	Diabetes/Impaired Glucose Metabolism	160
Quantification of bile acids: a mass spectrometry platform for studying gut microbe connection to metabolic diseases.	2020	Ibrahim Choucair	Journal of lipid research	GLCA	Blood Concentration	Case Control	Adults	Type 2 Diabetes Mellitus	Diabetes/Impaired Glucose Metabolism	160
Fecal bile acid excretion profile in gallstone patients.	1999	A Mamianetti	Medicina	DCA	Feces Concentration	Case Control	Adults	Gallstones	Hepatobiliary Disorders	20
Fecal bile acid excretion profile in gallstone patients.	1999	A Mamianetti	Medicina	LCA	Feces Concentration	Case Control	Adults	Gallstones	Hepatobiliary Disorders	20
Metabolomics and Incidence of Atrial Fibrillation in African Americans: The Atherosclerosis Risk in Communities (ARIC) Study.	2015	Alvaro Alonso	PloS one	GLCA	Blood Concentration	Cohort Study	Adults	Atrial Fibrillation	Cardiovascular Disorders	1919
Serum unconjugated primary and secondary bile acids in patients with cholangiocarcinoma and hepatocellular carcinoma.	1990	S Changbumrung	Journal of the Medical Association of Thailand = Chotmaihet thangphaet	LCA	Blood Concentration	Case Control	Adults	Cholangiocarcinoma Hepatocellular Carcinoma	Hepatobiliary Cancer	121
Serum unconjugated primary and secondary bile acids in patients with cholangiocarcinoma and hepatocellular carcinoma.	1990	S Changbumrung	Journal of the Medical Association of Thailand = Chotmaihet thangphaet	DCA	Blood Concentration	Case Control	Adults	Cholangiocarcinoma Hepatocellular Carcinoma	Hepatobiliary Cancer	121
Urinary metabolites of tryptophan, serotonin and norepinephrine in alcoholics.	1978	M I Akhter	Journal of studies on alcohol	Tryptamine	Urine Concentration	Case Control	Adults	Alcoholic Liver Disease Nonalcoholic Liver Disease	Hepatobiliary Disorders	44
Bile acid metabolism in patients with Crohn's disease in terminal ileum.	1986	L Tougaard	Scandinavian journal of gastroenterology	DCA	Feces Concentration	Case Control	Adults	Ileal Crohn's Disease	Inflammatory Bowel Disease	41
Bile acid metabolism in patients with Crohn's disease in terminal ileum.	1986	L Tougaard	Scandinavian journal of gastroenterology	LCA	Feces Concentration	Case Control	Adults	Ileal Crohn's Disease	Inflammatory Bowel Disease	41
The association of bile acid excretion and atherosclerotic coronary artery disease.	2011	Gideon Charach	Therapeutic advances in gastroenterology	DCA	Feces Concentration	Case Control	Adults	Coronary Artery Disease	Cardiovascular Disorders	75
The association of bile acid excretion and atherosclerotic coronary artery disease.	2011	Gideon Charach	Therapeutic advances in gastroenterology	LCA	Feces Concentration	Case Control	Adults	Coronary Artery Disease	Cardiovascular Disorders	75
Fecal steroids and colorectal cancer.	1987	R W Owen	Nutrition and cancer	DCA	Feces Concentration	Case Control	Adults	Colorectal Cancer	Colorectal Cancer	37

Fecal steroids and colorectal cancer.	1987	R W Owen	Nutrition and cancer	LCA	Feces Concentration	Case Control	Adults	Colorectal Cancer	Colorectal Cancer	37
Metabolism of [14C]spermidine and [14C]putrescine in normal volunteers and in cancer patients.	1978	M G Rosenblum	Cancer research	Putrescine	Blood Concentration	Case Control	Adults	Cancer Patients With Advanced Disease	Cancer (Excluding Colorectal and Hepatobiliary Cancer)	10
Polyamine excretion in the urine of cancer patients.	1983	D Depierre	Journal of clinical chemistry and clinical biochemistry. Zeitschrift fur klinische Chemie und klinische Biochemie	Putrescine	Urine Concentration	Case Control	Adults	Cancer	Cancer (Excluding Colorectal and Hepatobiliary Cancer)	56
Effect of deoxycholic acid on lipoprotein and apolipoprotein levels in patients with familial hypercholesterolemia.	1986	J A Leuven	Atherosclerosis	DCA	Oral Intake	Clinical Trial	Adults	Familial Hypercholesterolemia	Hepatobiliary Disorders	17
Deoxycholic Acid, a Metabolite of Circulating Bile Acids, and Coronary Artery Vascular Calcification in CKD.	2018	Anna Jovanovich	American journal of kidney diseases : the official journal of the National Kidney Foundation	DCA	Blood Concentration	Cohort Study	Adults	Coronary Artery Calcification<br>Chronic Kidney Disease	Renal Disorders	112
Deoxycholic Acid, a Metabolite of Circulating Bile Acids, and Coronary Artery Vascular Calcification in CKD.	2018	Anna Jovanovich	American journal of kidney diseases : the official journal of the National Kidney Foundation	DCA	Blood Concentration	Cohort Study	Adults	Coronary Artery Calcification<br>Chronic Kidney Disease	Cardiovascular Disorders	112
Serotonin metabolism in patients with carcinoid tumors: incidence of 5-hydroxytryptophan-secreting tumors.	1978	J M Feldman	Gastroenterology	Tryptamine	Urine Concentration	Case Control	Adults	Metastatic Carcinoid Tumors	Cancer (Excluding Colorectal and Hepatobiliary Cancer)	38
Bile acid indices as biomarkers for liver diseases I: Diagnostic markers.	2021	Jawaher Abdullah Alamoudi	World journal of hepatology	LCA	Urine Concentration	Case Control	Adults	Hepatobiliary Diseases	Hepatobiliary Disorders	403
Bile acid indices as biomarkers for liver diseases I: Diagnostic markers.	2021	Jawaher Abdullah Alamoudi	World journal of hepatology	DCA	Urine Concentration	Case Control	Adults	Hepatobiliary Diseases	Hepatobiliary Disorders	403
A new simple enzymatic assay method for urinary polyamines in humans.	1983	S Kubota	Cancer research	Tryptamine	Urine Concentration	Case Control	Adults	Stomach Cancer<br>Colon Cancer<br>Lung Cancer<br>Esophagus Cancer<br>Gall Bladder Cancer<br>Pancreas Cancer<br>Liver Cancer	Hepatobiliary Cancer	246

								Hematological Cancers<br>Diseases Other Than Cancer		
A new simple enzymatic assay method for urinary polyamines in humans.	1983	S Kubota	Cancer research	Tryptamine	Urine Concentration	Case Control	Adults	Stomach Cancer<br>Colon Cancer<br>Lung Cancer<br>Esophagus Cancer<br>Gall Bladder Cancer<br>Pancreas Cancer<br>Liver Cancer<br>Hematological Cancers<br>Diseases Other Than Cancer	Colorectal Cancer	246
A new simple enzymatic assay method for urinary polyamines in humans.	1983	S Kubota	Cancer research	Tryptamine	Urine Concentration	Case Control	Adults	Stomach Cancer<br>Colon Cancer<br>Lung Cancer<br>Esophagus Cancer<br>Gall Bladder Cancer<br>Pancreas Cancer<br>Liver Cancer<br>Hematological Cancers<br>Diseases Other Than Cancer	Cancer (Excluding Colorectal and Hepatobiliary Cancer)	246
A new simple enzymatic assay method for urinary polyamines in humans.	1983	S Kubota	Cancer research	Putrescine	Urine Concentration	Case Control	Adults	Stomach Cancer<br>Colon Cancer<br>Lung Cancer<br>Esophagus Cancer<br>Gall Bladder Cancer<br>Pancreas Cancer<br>Liver Cancer<br>Hematological Cancers<br>Diseases Other Than Cancer	Hepatobiliary Cancer	246
A new simple enzymatic assay method for urinary polyamines in humans.	1983	S Kubota	Cancer research	Putrescine	Urine Concentration	Case Control	Adults	Stomach Cancer<br>Colon Cancer<br>Lung Cancer<br>Esophagus Cancer<br>Gall Bladder Cancer<br>Pancreas Cancer<br>Liver Cancer<br>Hematological Cancers	Colorectal Cancer	246

								Diseases Other Than Cancer		
A new simple enzymatic assay method for urinary polyamines in humans.	1983	S Kubota	Cancer research	Putrescine	Urine Concentration	Case Control	Adults	Stomach Cancer<br>Colon Cancer<br>Lung Cancer<br>Esophagus Cancer<br>Gall Bladder Cancer<br>Pancreas Cancer<br>Liver Cancer<br>Hematological Cancers<br>Diseases Other Than Cancer	Cancer (Excluding Colorectal and Hepatobiliary Cancer)	246
Bile acids in patients suffering from colorectal carcinoma--a pilot study.	1983	W J Kurtz	The Tokai journal of experimental and clinical medicine	DCA	Blood Concentration	Case Control	Adults	Colorectal Carcinoma	Colorectal Cancer	32
Bile acids in patients suffering from colorectal carcinoma--a pilot study.	1983	W J Kurtz	The Tokai journal of experimental and clinical medicine	LCA	Blood Concentration	Case Control	Adults	Colorectal Carcinoma	Colorectal Cancer	32
Serum and bile lipid levels in patients with and without gallstones.	1996	W H Tang	Journal of gastroenterology	DCA	Blood Concentration	Case Control	Adults	Gallstones	Hepatobiliary Disorders	70
A quantitative and qualitative study of the transglutaminase-mediated insertion of polyamines into plasma proteins from patients with bronchopulmonary cancer.	1984	A M Roch	International journal of cancer	Putrescine	Blood Concentration	Case Control	Adults	Bronchopulmonary Cancer	Cancer (Excluding Colorectal and Hepatobiliary Cancer)	62
The measurement of sulphated and non-sulphated bile acids in serum using gas-liquid chromatography.	1975	C B Campbell	Clinica chimica acta; international journal of clinical chemistry	GDCA	Blood Concentration	Case Control	Adults	Acute Viral Hepatitis	Hepatobiliary Disorders	20
The measurement of sulphated and non-sulphated bile acids in serum using gas-liquid chromatography.	1975	C B Campbell	Clinica chimica acta; international journal of clinical chemistry	DCA	Blood Concentration	Case Control	Adults	Acute Viral Hepatitis	Hepatobiliary Disorders	20
The measurement of sulphated and non-sulphated bile acids in serum using gas-liquid chromatography.	1975	C B Campbell	Clinica chimica acta; international journal of clinical chemistry	LCA	Blood Concentration	Case Control	Adults	Acute Viral Hepatitis	Hepatobiliary Disorders	20
The measurement of sulphated and non-sulphated bile acids in serum using gas-liquid chromatography.	1975	C B Campbell	Clinica chimica acta; international journal of clinical chemistry	GLCA	Blood Concentration	Case Control	Adults	Acute Viral Hepatitis	Hepatobiliary Disorders	20
Polyamine levels and ornithine decarboxylase activity in blood and erythrocytes in human diseases.	2003	G Stabellini	International journal of clinical pharmacology research	Putrescine	Blood Concentration	Case Control	Adults	Breast Cancer<br>Lung Cancer<br>Colon Cancer<br>Familial Polyposis	Colorectal Cancer	117

Polyamine levels and ornithine decarboxylase activity in blood and erythrocytes in human diseases.	2003	G Stabellini	International journal of clinical pharmacology research	Putrescine	Blood Concentration	Case Control	Adults	Breast Cancer<br>Lung Cancer<br>Colon Cancer<br>Familial Polyposis	Cancer (Excluding Colorectal and Hepatobiliary Cancer)	117
Acrolein produced from polyamines as one of the uraemic toxins.	2003	K Sakata	Biochemical Society transactions	Putrescine	Blood Concentration	Case Control	Adults	Chronic Renal Failure	Renal Disorders	41
Effect of metastatic cancer on platelet monoamine oxidase activity and serotonin metabolism.	1979	J M Feldman	Cancer	Tryptamine	Urine Concentration	Case Control	Adults	Small Cell Carcinoma Of The Lung<br>Squamous Cell Carcinoma Of The Lung<br>Adenocarcinoma Of The Lung<br>Large Cell Carcinoma Of The Lung<br>Adenocarcinoma Of The Stomach<br>Hypernephroma<br>Hepatoma<br>Papillary Carcinoma Of The Thyroid<br>Small Cell Carcinoma Of The Thyroid<br>No	Hepatobiliary Cancer	105
Effect of metastatic cancer on platelet monoamine oxidase activity and serotonin metabolism.	1979	J M Feldman	Cancer	Tryptamine	Urine Concentration	Case Control	Adults	Small Cell Carcinoma Of The Lung<br>Squamous Cell Carcinoma Of The Lung<br>Adenocarcinoma Of The Lung<br>Large Cell Carcinoma Of The Lung<br>Adenocarcinoma Of The Stomach<br>Hypernephroma<br>Hepatoma<br>Papillary Carcinoma Of The Thyroid<br>Small Cell Carcinoma	Colorectal Cancer	105

								Of The Thyroid<br>No		
Effect of metastatic cancer on platelet monoamine oxidase activity and serotonin metabolism.	1979	J M Feldman	Cancer	Tryptamine	Urine Concentration	Case Control	Adults	Small Cell Carcinoma Of The Lung<br>Squamous Cell Carcinoma Of The Lung<br>Adenocarcinoma Of The Lung<br>Large Cell Carcinoma Of The Lung<br>Adenocarcinoma Of The Stomach<br>Hypernephroma<br>Hepatoma<br>Papillary Carcinoma Of The Thyroid<br>Small Cell Carcinoma Of The Thyroid<br>No	Cancer (Excluding Colorectal and Hepatobiliary Cancer)	105
Urinary polyamine levels in patients with psoriasis.	1979	S Sakakibara	Archives of dermatological research	Putrescine	Urine Concentration	Case Control	Adults	Psoriasis	Dermotological Disorders	23
Studies of serum and feces bile acids determination by gas chromatography-mass spectrometry.	2006	Tomoaki Tadano	Rinsho byori. The Japanese journal of clinical pathology	LCA	Blood Concentration	Case Control	Adults	Colon Cancer	Colorectal Cancer	42
Studies of serum and feces bile acids determination by gas chromatography-mass spectrometry.	2006	Tomoaki Tadano	Rinsho byori. The Japanese journal of clinical pathology	LCA	Feces Concentration	Case Control	Adults	Colon Cancer	Colorectal Cancer	42
Studies of serum and feces bile acids determination by gas chromatography-mass spectrometry.	2006	Tomoaki Tadano	Rinsho byori. The Japanese journal of clinical pathology	DCA	Blood Concentration	Case Control	Adults	Colon Cancer	Colorectal Cancer	42
Studies of serum and feces bile acids determination by gas chromatography-mass spectrometry.	2006	Tomoaki Tadano	Rinsho byori. The Japanese journal of clinical pathology	DCA	Feces Concentration	Case Control	Adults	Colon Cancer	Colorectal Cancer	42
Plasma Bile Acid Profile in Patients with and without Type 2 Diabetes.	2021	Alessandro Mantovani	Metabolites	GDCA	Blood Concentration	Case Control	Adults	Type 2 Diabetes Mellitus<br>Metabolic Syndrome	Metabolic Disorders	326
Plasma Bile Acid Profile in Patients with and without Type 2 Diabetes.	2021	Alessandro Mantovani	Metabolites	GDCA	Blood Concentration	Case Control	Adults	Type 2 Diabetes Mellitus<br>Metabolic Syndrome	Diabetes/Impaire d Glucose Metabolism	326

Plasma Bile Acid Profile in Patients with and without Type 2 Diabetes.	2021	Alessandro Mantovani	Metabolites	LCA	Blood Concentration	Case Control	Adults	Type 2 Diabetes Mellitus<br>Metabolic Syndrome	Metabolic Disorders	326
Plasma Bile Acid Profile in Patients with and without Type 2 Diabetes.	2021	Alessandro Mantovani	Metabolites	LCA	Blood Concentration	Case Control	Adults	Type 2 Diabetes Mellitus<br>Metabolic Syndrome	Diabetes/Impaired Glucose Metabolism	326
Plasma Bile Acid Profile in Patients with and without Type 2 Diabetes.	2021	Alessandro Mantovani	Metabolites	DCA	Blood Concentration	Case Control	Adults	Type 2 Diabetes Mellitus<br>Metabolic Syndrome	Metabolic Disorders	326
Plasma Bile Acid Profile in Patients with and without Type 2 Diabetes.	2021	Alessandro Mantovani	Metabolites	DCA	Blood Concentration	Case Control	Adults	Type 2 Diabetes Mellitus<br>Metabolic Syndrome	Diabetes/Impaired Glucose Metabolism	326
Bile salts, endotoxin and renal function in obstructive jaundice.	1987	C J Cahill	Surgery, gynecology & obstetrics	DCA	Oral Intake	Clinical Trial	Adults	Obstructive Jaundice	Hepatobiliary Disorders	46
Decreased fecal bile acid output in patients with coronary atherosclerosis.	1998	G Charach	Journal of medicine	DCA	Feces Concentration	Case Control	Adults	Coronary Artery Disease	Cardiovascular Disorders	57
Decreased fecal bile acid output in patients with coronary atherosclerosis.	1998	G Charach	Journal of medicine	LCA	Feces Concentration	Case Control	Adults	Coronary Artery Disease	Cardiovascular Disorders	57
Polyamine levels as biomarkers of injury response in polytrauma victims.	1989	M Jeevanandam	Metabolism: clinical and experimental	Putrescine	Urine Concentration	Case Control	Adults	Multiple Trauma	Other	21
D-glutamate, D-serine, and D-alanine differ in their roles in cognitive decline in patients with Alzheimer's disease or mild cognitive impairment.	2019	Chieh-Hsin Lin	Pharmacology, biochemistry, and behavior	d-Alanine	Blood Concentration	Case Control	Adults	Alzheimer'S Disease<br>Mild Cognitive Impairment	Neurological Disorders	144
Rapid detection of polyamines in the sera of patients with colorectal carcinoma by liquid ion-exchange chromatography.	1980	H Desser	Oncology	Putrescine	Blood Concentration	Case Control	Adults	Colorectal Carcinoma	Colorectal Cancer	58
Serum levels of polyamines in patients with chronic renal failure.	1983	A Saito	Kidney international. Supplement	Putrescine	Blood Concentration	Case Control	Adults	Chronic Renal Failure	Renal Disorders	46
A metabonomic approach identifies human urinary PAG as a novel marker of interstitial cystitis.	2009	Yousuke Fukui	Journal of chromatography. B, Analytical technologies in the biomedical and life sciences	PAG	Urine Concentration	Case Control	Adults	Interstitial Cystitis<br>Bacterial Cystitis	Digestive Disorders	40
Kinetic analysis of bile acids in the feces of colorectal cancer patients by gas chromatography-mass spectrometry (GC-MS).	2007	Tomoaki Tadano	Rinsho byori. The Japanese journal of clinical pathology	LCA	Feces Concentration	Case Control	Adults	Colorectal Cancer	Colorectal Cancer	192
Kinetic analysis of bile acids in the feces of colorectal cancer patients by gas chromatography-mass spectrometry (GC-MS).	2007	Tomoaki Tadano	Rinsho byori. The Japanese journal of clinical pathology	DCA	Feces Concentration	Case Control	Adults	Colorectal Cancer	Colorectal Cancer	192
A prospective study of faecal bile acids and colorectal cancer.	2000	A Haines	European journal of cancer prevention : the official journal	LCA	Feces Concentration	Case Control	Adults	Colorectal Cancer	Colorectal Cancer	238

			of the European Cancer Prevention Organisation (ECP)							
A prospective study of faecal bile acids and colorectal cancer.	2000	A Haines	European journal of cancer prevention : the official journal of the European Cancer Prevention Organisation (ECP)	DCA	Feces Concentration	Case Control	Adults	Colorectal Cancer	Colorectal Cancer	238
Plasma levels of phenylacetic acid, m- and p-hydroxyphenylacetic acid, and platelet monoamine oxidase activity in schizophrenic and other patients.	1982	B A Davis	Psychiatry research	Tryptamine	Blood Concentration	Case Control	Adults	Schizophrenic	Mental Disorders	56
Plasma bile acid levels and liver disease.	1981	I Magyar	Acta medica Academiae Scientiarum Hungaricae	DCA	Blood Concentration	Case Control	Adults	Liver And Billiary Tract Disease	Hepatobiliary Disorders	153
Deoxycholic acid treatment in patients with cholesterol gallstones: failure to detect a suppression of cholesterol 7alpha-hydroxylase activity.	1999	C Hillebrant	Journal of internal medicine	DCA	Oral Intake	Clinical Trial	Adults	Cholesterol Gallstone	Hepatobiliary Disorders	41
Prevention of postoperative renal failure in patients with obstructive jaundice--the role of bile salts.	1983	C J Cahill	The British journal of surgery	DCA	Oral Intake	Clinical Trial	Adults	Renal Failure<br>Obstructive Jaundice	Renal Disorders	58
Prevention of postoperative renal failure in patients with obstructive jaundice--the role of bile salts.	1983	C J Cahill	The British journal of surgery	DCA	Oral Intake	Clinical Trial	Adults	Renal Failure<br>Obstructive Jaundice	Hepatobiliary Disorders	58
Determination of polyamines in hydrolysates of uremic plasma by high-performance cation-exchange column chromatography.	1983	T Takagi	Journal of chromatography	Putrescine	Urine Concentration	Case Control	Adults	Renal Failure	Renal Disorders	18
Urinary polyamine levels in human cancer.	1975	A Lipton	Cancer	Putrescine	Urine Concentration	Case Control	Adults	Cancer, Unclassified	Cancer (Excluding Colorectal and Hepatobiliary Cancer)	98
Diagnostic effectiveness of serum bile acids in liver diseases as evaluated by multivariate statistical methods.		D Festi	Hepatology (Baltimore, Md.)	LCA	Blood Concentration	Case Control	Adults	Acute Hepatitis<br>Liver Fibrosis<br>Liver Steatosis<br>Chronic Persistent Hepatitis<br>Chronic Active Hepatitis<br>Cholestasis Due To Extrahepatic Biliary Obstruction	Hepatobiliary Disorders	415

Variation of serum bile acids in patients with colorectal adenomas during a one-year follow-up.	1994	E Bayerdörffer	Digestion	LCA	Blood Concentration	Case Control	Adults	Colorectal Adenomas	Digestive Disorders	44
Variation of serum bile acids in patients with colorectal adenomas during a one-year follow-up.	1994	E Bayerdörffer	Digestion	DCA	Blood Concentration	Case Control	Adults	Colorectal Adenomas	Digestive Disorders	44
Plasma bile acid concentrations in patients with human immunodeficiency virus infection receiving protease inhibitor therapy: possible implications for hepatotoxicity.	2010	MaryPeace McRae	Pharmacotherapy	DCA	Blood Concentration	Case Control	Adults	HIV	Other	11
Plasma bile acid concentrations in patients with human immunodeficiency virus infection receiving protease inhibitor therapy: possible implications for hepatotoxicity.	2010	MaryPeace McRae	Pharmacotherapy	LCA	Blood Concentration	Case Control	Adults	HIV	Other	11
Fecal bile acid excretion pattern in cholecystectomized patients.	1986	N F Breuer	Digestive diseases and sciences	LCA	Feces Concentration	Case Control	Adults	Arterial Hypertension Cardiac Dysrhythmias Ischemic Heart Disease Thrombophlebitis Psoriasis	Dermotological Disorders	51
Fecal bile acid excretion pattern in cholecystectomized patients.	1986	N F Breuer	Digestive diseases and sciences	LCA	Feces Concentration	Case Control	Adults	Arterial Hypertension Cardiac Dysrhythmias Ischemic Heart Disease Thrombophlebitis Psoriasis	Cardiovascular Disorders	51
Fecal bile acid excretion pattern in cholecystectomized patients.	1986	N F Breuer	Digestive diseases and sciences	DCA	Feces Concentration	Case Control	Adults	Arterial Hypertension Cardiac Dysrhythmias Ischemic Heart Disease Thrombophlebitis Psoriasis	Dermotological Disorders	51
Fecal bile acid excretion pattern in cholecystectomized patients.	1986	N F Breuer	Digestive diseases and sciences	DCA	Feces Concentration	Case Control	Adults	Arterial Hypertension Cardiac Dysrhythmias Ischemic Heart Disease Thrombophlebitis Psoriasis	Cardiovascular Disorders	51
Pathogenesis of chronic cluster headache and bouts: role of tryptamine, arginine metabolism and α.	2017	G D'Andrea	Neurological sciences : official journal of the Italian Neurological Society and of the	Tryptamine	Blood Concentration	Case Control	Adults	Chronic Cluster Headache	Neurological Disorders	51

			Italian Society of Clinical Neurophysiology							
Urinary polyamine levels in patients with localized malignancy.	1976	A Lipton	Cancer	Putrescine	Urine Concentration	Case Control	Adults	Genitourinary 14 Breast 1 Gastrointestinal 7 Lung 10 Gynecologic 16 Pituitary 1	Cancer (Excluding Colorectal and Hepatobiliary Cancer)	49
Elevated levels of peripheral-blood, naturally occurring aliphatic polyamines in bronchial asthmatic patients with active symptoms.	1992	M Kurosawa	Allergy	Putrescine	Blood Concentration	Case Control	Adults	Bronchial Asthma	Respiratory Disorders	77
Polyamine excretion in the urine of cancer patients.	1975	F Dreyfuss	Israel journal of medical sciences	Putrescine	Urine Concentration	Case Control	Adults	Gastrointestinal Cancer<br>Kidney And Genitourinary Tract Cancer<br>Respiratory Tract Cancer<br>Multiple Myeoma Cancer<br>Malignant Lymphomas Cancer<br>Miscellaneous	Cancer (Excluding Colorectal and Hepatobiliary Cancer)	96
Urinary excretion of polyamines in the adult respiratory distress syndrome.		J E Heffner	Experimental lung research	Putrescine	Urine Concentration	Case Control	Adults	Respiratory Distress Syndrome	Respiratory Disorders	32
Effect of hyper- and hypothyroidism on platelet monoamine oxidase activity and serotonin metabolism.	1977	J M Feldman	Metabolism: clinical and experimental	Tryptamine	Urine Concentration	Case Control	Adults	Hyperthyroid<br>Hypothyroid	Other	64
Increase in putrescine, amine oxidase, and acrolein in plasma of renal failure patients.	2003	Kaori Sakata	Biochemical and biophysical research communications	Putrescine	Blood Concentration	Case Control	Adults	Chronic Renal Failure	Renal Disorders	40
Urinary polyamines in colorectal cancer.	1986	J S Thompson	Diseases of the colon and rectum	Putrescine	Urine Concentration	Case Control	Adults	Colorectal Cancer<br>Inflammatory Bowel Disease<br>Benigh Colorectal Disease	Inflammatory Bowel Disease	30
Urinary polyamines in colorectal cancer.	1986	J S Thompson	Diseases of the colon and rectum	Putrescine	Urine Concentration	Case Control	Adults	Colorectal Cancer<br>Inflammatory Bowel Disease<br>Benigh Colorectal Disease	Colorectal Cancer	30
The diagnostic and prognostic value of urinary polyamine measurement in bladder cancer.	1981	P Pastorini	Urological research	Putrescine	Urine Concentration	Case Control	Adults	Bladder Cancer	Cancer (Excluding	54

									Colorectal and Hepatobiliary Cancer)	
Putrescine, spermidine, N-acetylspermidine and spermine in the urine of patients with leukaemias and tumors.	1975	M Tsuji	Clinica chimica acta; international journal of clinical chemistry	Putrescine	Urine Concentration	Case Control	Adults	Chronic Leukaemia<br>Reticulocyte Sarcoma<br>Reticulum Ceil Sarcoma<br>Multiple Myeloma<br>Crania-Pharyngioma<br>Breast Cancer<br>Maxillary Cancer<br>Osteosarcoma<br>Stomach Cancer	Cancer (Excluding Colorectal and Hepatobiliary Cancer)	38
Results of the HEMO Study suggest that p-cresol sulfate and indoxyl sulfate are not associated with cardiovascular outcomes.	2017	Tariq Shafi	Kidney international	PAG	Blood Concentration	Cohort Study	Adults	Cardiac Death, Sudden Cardiac Death, And First Cardiovascular Event<br>Hemodialysis	Renal Disorders	1273
Results of the HEMO Study suggest that p-cresol sulfate and indoxyl sulfate are not associated with cardiovascular outcomes.	2017	Tariq Shafi	Kidney international	PAG	Blood Concentration	Cohort Study	Adults	Cardiac Death, Sudden Cardiac Death, And First Cardiovascular Event<br>Hemodialysis	Cardiovascular Disorders	1273
Urinary polyamines for evaluating the course of disease for patients with small cell carcinoma of the lung.	1983	K B Woo	Cancer	Putrescine	Urine Concentration	Case Control	Adults	Small Cell Carcinoma Of The Lung	Cancer (Excluding Colorectal and Hepatobiliary Cancer)	64
Significance of fecal deoxycholic acid concentration for colorectal tumor enlargement.	2010	Atsuko Kawano	Asian Pacific journal of cancer prevention : APJCP	DCA	Feces Concentration	Cohort Study	Adults	Colorectal Cancer	Colorectal Cancer	390
Significance of fecal deoxycholic acid concentration for colorectal tumor enlargement.	2010	Atsuko Kawano	Asian Pacific journal of cancer prevention : APJCP	DCA	Feces Concentration	Case Control	Adults	Colorectal Cancer	Colorectal Cancer	390
Plasma deoxycholic acid concentration is elevated in postmenopausal women with newly diagnosed breast cancer.	2002	V Costarelli	European journal of clinical nutrition	DCA	Blood Concentration	Case Control	Adults	Breast Cancer	Cancer (Excluding Colorectal and Hepatobiliary Cancer)	40
Polyamines as markers of response and disease activity in cancer chemotherapy.	1977	B G Durie	Cancer research	Putrescine	Urine Concentration	Case Control	Adults	Hematological Neoplasms<br>Solid Neoplasms	Cancer (Excluding Colorectal and	139

									Hepatobiliary Cancer)	
Fecal bile acids in two Japanese populations with different colon cancer risks.	1979	H F Mower	Cancer research	LCA	Feces Concentration	Case Control	Adults	Colon Cancer Risk	Colorectal Cancer	235
Fecal bile acids in two Japanese populations with different colon cancer risks.	1979	H F Mower	Cancer research	DCA	Feces Concentration	Case Control	Adults	Colon Cancer Risk	Colorectal Cancer	235
Serum bile acid concentrations in mild liver cirrhosis.	1993	A V Greco	Clinica chimica acta; international journal of clinical chemistry	GDCA	Blood Concentration	Case Control	Adults	Liver Cirrhosis	Hepatobiliary Disorders	40
Serum bile acid concentrations in mild liver cirrhosis.	1993	A V Greco	Clinica chimica acta; international journal of clinical chemistry	GLCA	Blood Concentration	Case Control	Adults	Liver Cirrhosis	Hepatobiliary Disorders	40
Fecal bile acids and neutral sterols in patients with familial polyposis.	1976	B S Reddy	Cancer	DCA	Feces Concentration	Case Control	Adults	Familial Polyposis	Digestive Disorders	35
Fecal bile acids and neutral sterols in patients with familial polyposis.	1976	B S Reddy	Cancer	LCA	Feces Concentration	Case Control	Adults	Familial Polyposis	Digestive Disorders	35
PAG and hippuric acid in uremic and healthy subjects.	1990	L Zimmerman	Nephron	PAG	Urine Concentration	Case Control	Adults	Uremic	Renal Disorders	27
PAG and hippuric acid in uremic and healthy subjects.	1990	L Zimmerman	Nephron	PAG	Blood Concentration	Case Control	Adults	Uremic	Renal Disorders	27
Prevention of postoperative renal dysfunction in patients with obstructive jaundice: a multicentre study of bile salts and lactulose.	1991	J A Pain	The British journal of surgery	DCA	Oral Intake	Clinical Trial	Adults	Postoperative Renal Dysfunction Obstructive Jaundice	Hepatobiliary Disorders	92
Prevention of postoperative renal dysfunction in patients with obstructive jaundice: a multicentre study of bile salts and lactulose.	1991	J A Pain	The British journal of surgery	DCA	Oral Intake	Clinical Trial	Adults	Postoperative Renal Dysfunction Obstructive Jaundice	Renal Disorders	92
Metabolic Profiling of Impaired Cognitive Function in Patients Receiving Dialysis.	2016	Manjula Kurella Tamura	Journal of the American Society of Nephrology : JASN	PAG	Blood Concentration	Cross-Sectional	Adults	Dialysis	Renal Disorders	321
The content of unbound polyamines in blood plasma and leukocytes of patients with polycythemia vera.	1975	H Desser	Clinica chimica acta; international journal of clinical chemistry	Putrescine	Blood Concentration	Case Control	Adults	Polycythemia	Other	14
Faecal bile acids and the irritable colon syndrome.	1981	M Flynn	Digestion	DCA	Feces Concentration	Case Control	Adults	Irritable Colon Syndrome	Digestive Disorders	34
Faecal bile acids and the irritable colon syndrome.	1981	M Flynn	Digestion	LCA	Feces Concentration	Case Control	Adults	Irritable Colon Syndrome	Digestive Disorders	34
Serum bile acids in patients with hyperlipidaemia.	1978	C R Pennington	Journal of clinical pathology	LCA	Blood Concentration	Case Control	Adults	Hyperlipidaemia	Metabolic Disorders	26
Serum bile acids in patients with hyperlipidaemia.	1978	C R Pennington	Journal of clinical pathology	DCA	Blood Concentration	Case Control	Adults	Hyperlipidaemia	Metabolic Disorders	26
Biologic markers in breast carcinoma: clinical correlations with urinary polyamines.	1980	D C Tormey	Cancer	Putrescine	Urine Concentration	Case Control	Adults	Breast Carcinoma	Cancer (Excluding	142

									Colorectal and Hepatobiliary Cancer)	
Serum metabolome and targeted bile acid profiling reveals potential novel biomarkers for drug-induced liver injury.	2019	Zhenhua Ma	Medicine	DCA	Blood Concentration	Case Control	Adults	Drug-Induced Liver Injury	Hepatobiliary Disorders	68
Serum metabolome and targeted bile acid profiling reveals potential novel biomarkers for drug-induced liver injury.	2019	Zhenhua Ma	Medicine	LCA	Blood Concentration	Case Control	Adults	Drug-Induced Liver Injury	Hepatobiliary Disorders	68
Changes in serum bile acid concentrations during normal pregnancy, in patients with intrahepatic cholestasis of pregnancy and in pregnant women with itching.	1981	J Heikkinen	British journal of obstetrics and gynaecology	DCA	Blood Concentration	Case Control	Pregnant women	Intrahepatic Cholestasis Itching	Hepatobiliary Disorders	124
Changes in serum bile acid concentrations during normal pregnancy, in patients with intrahepatic cholestasis of pregnancy and in pregnant women with itching.	1981	J Heikkinen	British journal of obstetrics and gynaecology	DCA	Blood Concentration	Case Control	Pregnant women	Intrahepatic Cholestasis Itching	Dermotological Disorders	124
Relevance of serum bile acid profile in the diagnosis of intrahepatic cholestasis of pregnancy in an high incidence area: Portugal.	1998	D Brites	European journal of obstetrics, gynecology, and reproductive biology	LCA	Blood Concentration	Case Control	Pregnant women	Intrahepatic Cholestasis	Hepatobiliary Disorders	97
Relevance of serum bile acid profile in the diagnosis of intrahepatic cholestasis of pregnancy in an high incidence area: Portugal.	1998	D Brites	European journal of obstetrics, gynecology, and reproductive biology	DCA	Blood Concentration	Case Control	Pregnant women	Intrahepatic Cholestasis	Hepatobiliary Disorders	97
Relevance of serum bile acid profile in the diagnosis of intrahepatic cholestasis of pregnancy in an high incidence area: Portugal.	1998	D Brites	European journal of obstetrics, gynecology, and reproductive biology	GLCA	Blood Concentration	Case Control	Pregnant women	Intrahepatic Cholestasis	Hepatobiliary Disorders	97
Relevance of serum bile acid profile in the diagnosis of intrahepatic cholestasis of pregnancy in an high incidence area: Portugal.	1998	D Brites	European journal of obstetrics, gynecology, and reproductive biology	GDCA	Blood Concentration	Case Control	Pregnant women	Intrahepatic Cholestasis	Hepatobiliary Disorders	97
Reduced glycodeoxycholic acid levels are associated with negative clinical outcomes of gestational diabetes mellitus.	2021	Bo Zhu	Journal of Zhejiang University. Science. B	GDCA	Blood Concentration	Case Control	Pregnant women	Gestational Diabetes Mellitus	Diabetes/Impaire d Glucose Metabolism	115
Reduced glycodeoxycholic acid levels are associated with negative clinical outcomes of gestational diabetes mellitus.	2021	Bo Zhu	Journal of Zhejiang University. Science. B	LCA	Blood Concentration	Case Control	Pregnant women	Gestational Diabetes Mellitus	Diabetes/Impaire d Glucose Metabolism	115
Reduced glycodeoxycholic acid levels are associated with negative clinical outcomes of gestational diabetes mellitus.	2021	Bo Zhu	Journal of Zhejiang University. Science. B	GLCA	Blood Concentration	Case Control	Pregnant women	Gestational Diabetes Mellitus	Diabetes/Impaire d Glucose Metabolism	115
Reduced glycodeoxycholic acid levels are associated with negative clinical outcomes of gestational diabetes mellitus.	2021	Bo Zhu	Journal of Zhejiang University. Science. B	DCA	Blood Concentration	Case Control	Pregnant women	Gestational Diabetes Mellitus	Diabetes/Impaire d Glucose Metabolism	115

Circulating lysophosphatidylcholines in early pregnancy and risk of gestational diabetes in Chinese women.	2020	Liu, J.	Journal of Clinical Endocrinology and Metabolism	DCA	Blood Concentration	Case Control	Pregnant women	Gestational Diabetes	Diabetes/Impaired Glucose Metabolism	486
Coenzyme Q in pregnant women and rats with intrahepatic cholestasis.	2014	Manuela R Martinefski	Liver international : official journal of the International Association for the Study of the Liver	DCA	Blood Concentration	Case Control	Pregnant women	Intrahepatic Cholestasis	Hepatobiliary Disorders	56
Coenzyme Q in pregnant women and rats with intrahepatic cholestasis.	2014	Manuela R Martinefski	Liver international : official journal of the International Association for the Study of the Liver	LCA	Blood Concentration	Case Control	Pregnant women	Intrahepatic Cholestasis	Hepatobiliary Disorders	56
Relationship between asymptomatic hypercholanaemia of pregnancy and progesterone metabolism.	2002	Pascual, M. J.	Clinical Science	LCA	Blood Concentration	Case Control	Pregnant women	Asymptomatic Hypercholanaemia	Metabolic Disorders	411
Relationship between asymptomatic hypercholanaemia of pregnancy and progesterone metabolism.	2002	Pascual, M. J.	Clinical Science	DCA	Blood Concentration	Case Control	Pregnant women	Asymptomatic Hypercholanaemia	Metabolic Disorders	411
Serum bile acids in the early diagnosis of intrahepatic cholestasis of pregnancy.	1983	Heikkinen, J.	Obstet Gynecol	DCA	Blood Concentration	Case Control	Pregnant women	Intrahepatic Cholestasis	Hepatobiliary Disorders	75
Bile acids in maternal serum, umbilical cord serum and amniotic fluid of healthy women, women with pruritis and patients with intrahepatic cholestasis of pregnancy.	1983	Heikkinen, J.	Journal of Obstetrics and Gynaecology	DCA	Blood Concentration	Case Control	Pregnant women	Intrahepatic Cholestasis Pruritus	Dermotological Disorders	97
Bile acids in maternal serum, umbilical cord serum and amniotic fluid of healthy women, women with pruritis and patients with intrahepatic cholestasis of pregnancy.	1983	Heikkinen, J.	Journal of Obstetrics and Gynaecology	DCA	Blood Concentration	Case Control	Pregnant women	Intrahepatic Cholestasis Pruritus	Hepatobiliary Disorders	97
Promoter DNA methylation of farnesoid X receptor and pregnane X receptor modulates the intrahepatic cholestasis of pregnancy phenotype.	2014	Romina Cabrerizo	PloS one	GDCA	Blood Concentration	Case Control	Pregnant women	Intrahepatic Cholestasis	Hepatobiliary Disorders	261
Promoter DNA methylation of farnesoid X receptor and pregnane X receptor modulates the intrahepatic cholestasis of pregnancy phenotype.	2014	Romina Cabrerizo	PloS one	DCA	Blood Concentration	Case Control	Pregnant women	Intrahepatic Cholestasis	Hepatobiliary Disorders	261
Bile acid metabolites in early pregnancy and risk of gestational diabetes in Chinese women: A nested case-control study.	2018	Jing Li	EBioMedicine	GDCA	Blood Concentration	Case Control	Pregnant women	Gestational Diabetes	Diabetes/Impaired Glucose Metabolism	486
Bile acid metabolites in early pregnancy and risk of gestational diabetes in Chinese women: A nested case-control study.	2018	Jing Li	EBioMedicine	DCA	Blood Concentration	Case Control	Pregnant women	Gestational Diabetes	Diabetes/Impaired Glucose Metabolism	486

Integrated metabolome analysis reveals novel connections between maternal fecal metabolome and the neonatal blood metabolome in women with gestational diabetes mellitus.	2020	Chunchao Zhao	Scientific reports	Putrescine	Feces Concentration	Case Control	Pregnant women	Gestational Diabetes Mellitus	Diabetes/Impaired Glucose Metabolism	40

Abbreviations

DCA: Deoxycholate (Deoxycholic Acid); GDCA: Glycodeoxycholate (Glycodeoxycholic Acid); LCA: Lithocholate (Lithocholic Acid); GLCA: Glycolithocholate (Glycolithocholic Acid); PAG: Phenylacetylglutamine.

*A study may have multiple rows. Each row represents a combination of one metabolite and one health outcome classification included in this study.
